# Genome-wide identification and evolutionary view of ALOG gene family in Solanaceae

**DOI:** 10.1590/1415-4757-GMB-2023-0142

**Published:** 2023-12-01

**Authors:** Caroline Turchetto, Ariadne de Castro Silvério, Edgar Luis Waschburger, Maria Eduarda Gonçalves Lacerda, Isadora Vieira Quintana, Andreia Carina Turchetto-Zolet

**Affiliations:** 1 Universidade Federal do Rio Grande do Sul, Instituto de Biociências, Programa de Pós-Graduação em Botânica (PPGBOT), Departamento de Botânica, Porto Alegre, RS, Brazil; 2 Universidade Federal do Rio Grande do Sul, Instituto de Biociências, Programa de Pós-Graduação em Genética e Biologia Molecular (PPGBM), Departamento de Genética, Porto Alegre, RS, Brazil.

**Keywords:** ALOG genes, transcription factor, plant development, floral architecture

## Abstract

The ALOG gene family, which was named after its earliest identified members ( *Arabidopsis* LSH1 and *Oryza* G1), encodes a class of transcription factors (TF) characterized by the presence of a highly conserved ALOG domain. These proteins are found in various plant species playing regulatory roles in plant growth, development, and morphological diversification of inflorescence. The functional characterization of these genes in some plant species has demonstrated their involvement in floral architecture. In this study, we used a genome-wide and phylogenetic approach to gain insights into plants’ origin, diversification, and functional aspects of the ALOG gene family. In total, 648 ALOG homologous genes were identified in 77 Viridiplantae species, and their evolutionary relationships were inferred using maximum likelihood phylogenetic analyses. Our results suggested that the ALOG gene family underwent several rounds of gene duplication and diversification during angiosperm evolution. Furthermore, we found three functional orthologous groups in Solanaceae species. The study provides insights into the evolutionary history and functional diversification of the ALOG gene family, which could aid in understanding the mechanisms underlying floral architecture in angiosperms.

## Introduction

The ALOG ( *Arabidopsis thaliana* LSH1 and *Oryza sativa* G1) gene family ( Zhao et al., 2004; Yoshida et al., 2009) is a class of transcription factors present in the viridiplantae species. The ALOG protein contains a highly conserved domain across phylogenetic distant plant lineages ~125 amino acids; (Pfam: PF04852; InterPro IPR006936) ( Xiao et al., 2018; Chen et al., 2019; Naramoto et al., 2020). There are reports of ALOG genes in the genomes of several land plants, such as mosses, liverworts, monocots, eudicots, and charophyte algae closely related to land plants. These findings suggest that the emergence of the ALOG gene family occurred before or during the plant terrestrialization process, exhibiting functional conservation and diversification during the evolution of land plants ( Naramoto *et al*., 2020). In fact, according to Xiao *et al*. (2018), multiple independent gene duplication events of ALOG genes occurred in different plant lineages.

Although their function is not fully characterized ( Chen et al., 2019; Naramoto et al., 2020), studies suggest that ALOG proteins play regulatory roles in various aspects of plant growth and development in different lineages of land plants ( Malcomber and Kellogg, 2004; Chen *et al.*, 2019; Naramoto *et al.*, 2019, 2020). In rice, the specification of sterile lemma identity is governed by the gene *LONG STERILE LEMMA1* (G1), and the inflorescence architecture is regulated by *TAWAWA1* (TAW1), which promotes inflorescence meristem activity and suppresses the phase change to spikelet meristem identity ( Yoshida et al., 2009, 2013). The ALOG gene of *Arabidopsis thaliana LIGHT-DEPENDENT SHORT HYPOCOTYLS 1* (LSH1) displays a dominant short hypocotyl phenotype in response to light (red, blue, and far-red) ( Cho and Zambryski, 2011). Meanwhile, LSH4 and LSH3 *A. thaliana* genes are known to suppress organ differentiation in the boundary region of the shoot apical meristem ( Takeda et al., 2011). The *Marchantia polymorpha LATERAL ORGAN SUPRESSOR 1* (MpLOS1) gene is essential for meristem maintenance and acts in the liverwort by repressing lateral organ growth ( Naramoto *et al*., 2019, 2020). It has been shown that ALOG domains are also present in specific plant defense proteins in *Arabidopsis*, *Brassica*, and *Sorghum* ( Iyer and Aravind, 2012).

Studies suggested that these transcription factors regulate reproductive growth in angiosperms. For example, in *Torenia fournieri*, the expression of the TfALOG3 gene occurs in the corolla tube and is linked to its differentiation and development ( Xiao et al., 2018, 2019). In rice, the functional analyses of OsG1L1 and OsG1L2 provide evidence of the role of these genes in inflorescence development ( Beretta et al., 2023). Overexpression of the LSH4 and LSH3 ALOG genes in *Arabidopsis*, induces extra flower differentiation within a flower ( Takeda et al., 2011). In tomato ( *Solanum lycopersicum)*, the ALOG family includes twelve members, which are named TMF FAMILY MEMBERs (TFAMs) ( Xu et al., 2016) that influence inflorescence organization ( MacAlister et al., 2012; Huang et al., 2018, 2022). The ALOG genes were also found in different tissues of *Petunia*, suggesting a spatial pattern of expression and different functions in regulating and developing various organs ( Chen et al., 2019).

Flowers and inﬂorescences of angiosperms show a wealth of distinct architectures, evidencing their importance for reproductive success, primarily related to pollinators ( Harder and Prusinkiewicz, 2013). The observations of functional characterization of ALOG family of proteins gave some insight into its role in floral diversification. Thus, these proteins are compelling candidates for research in groups of plants with great floral diversity associated with the emergence of new species, such as Solanaceae family. Solanaceae is a well-known angiosperm family because it includes many crops, ornamental plants, and species considered biological model systems, such as *Petunia* spp., *Solanum* spp., *Capsicum* spp., *Nicotiana* spp., and *Datura* spp. ( Olmstead et al., 2008; Särkinen et al., 2013). Solanaceae is an interesting family to study the diversification and relationship with its pollinator because it presents a diversity of reproductive structures related to a shift in pollinators ( Knapp, 2010). For example, in the genus *Nicotiana* a diversity of floral shapes can be found impacting the relationship with different pollinators and consequently the genus evolution ( Kaczorowski et al., 2012; Teixeira et al., 2022). 

In this study, we conduct a genome-wide identification and evolutionary analysis of the ALOG family of proteins across Streptophyta species to investigate the origin and diversification of ALOG gene family. Focusing on the Solanaceae family, we explored the evolution and function of ALOG genes within some genera within this family to gain insights into the role of ALOGs in the floral diversity in these taxa.

## Material and Methods

### Database search and sequences retrieval

The ALOG genes were identified through BLASTx and BLASTp searches against protein sequences derived from fully sequenced genomes of target species available in public databases ( NCBI, MarpolBase, PhycoCosm, Phytozome 13, Ensembl Plants, FernBase, TreeGenes (Congenie database), *Klebsormidium* Genome Project, Sol Genomics Network, Hornworts) ( Table S1). To conduct BLAST searches, we used the AtLSH6 CDS and protein sequences from *A. thaliana* (AT1G07090.1; Yoshida et al., 2009) as a query. This gene was selected as a query after a preliminary BLAST test with all *A. thaliana* ALOG (AtLSH1-10) sequences against the genome of seven representative and phylogenetically distant species. The results indicated that each ALOG protein from *A. thaliana* recovered the same sequences for all species; thus, the statistical scores and e-values were considered to select the AtLSH6 to use as a query ( Table S2). Only hits obtained with an e-value below 1.00E-10 were considered in further analyses. We chose the ALOG sequence of *A. thaliana* as a query because this is a species in which ALOG genes were first identified and characterized. 

To investigate the origin and evolution of the ALOG gene family of protein, we used a genome-wide identification of ALOG genes in Viridiplantae and one Rodophyta species. The sampling strategies included species representing most evolutionary lineages within the Streptophyte clade to identify the origin and diversification of ALOG genes. The species selected for BLAST searches are shown in Table S1 as follows: one Rhodophyta algae, ten species of green algae); 12 of six distinct major evolutionary lineages of Charophyte green algae (Mesostigmatophyceae, Chlorokybophyceae, Klebsormidiophyceae, Zygnematophyceae, Charophyceae, and Coleochaetophyceae; McCourt et al., 2004); three early diverging extant land plant lineages (seven species; liverworts, mosses, hornworts); two lycophyte species; three ferns species; three species from “gymnosperms”, and species belong to different clades of angiosperms (two species from ANA-grade, two Magnoliids, eight monocots, four basal eudicots,26 Eudicots Asterids, and 11 Eudicots Rosids; APG IV, 2016). These 91 species were randomly selected mainly because the complete genome was sequenced and annotated. To avoid redundancy, we select species from distinct taxonomic groups to balance the number of species in each group. Within the Eudicot Asterid, 20 species belonging to the Solanaceae family were included because we want to deepen the study of ALOG genes in this family. Details of species, the genomic database searched, BLAST statistics, and the number of genes retrieved for each species can be found in Tables S1 and S3.

After Blast searching against these 91 species ( Table S3), the sequences were filtered by the ALOG domain (Pfam: PF04852) presence using HMMER ( Eddy, 2011). In this step, we removed sequences containing two or more instances of the ALOG domain and those with incomplete ALOG domains. After filtering, we have 648 ALOG sequences from 77 species ( Table S1), which were separated into two datasets for phylogenetic analysis. Details of sequence retrieval and filtering steps are shown in a scheme in Figure S1). 

### Alignment and phylogenetic analyses 

**Figure 1 -:**
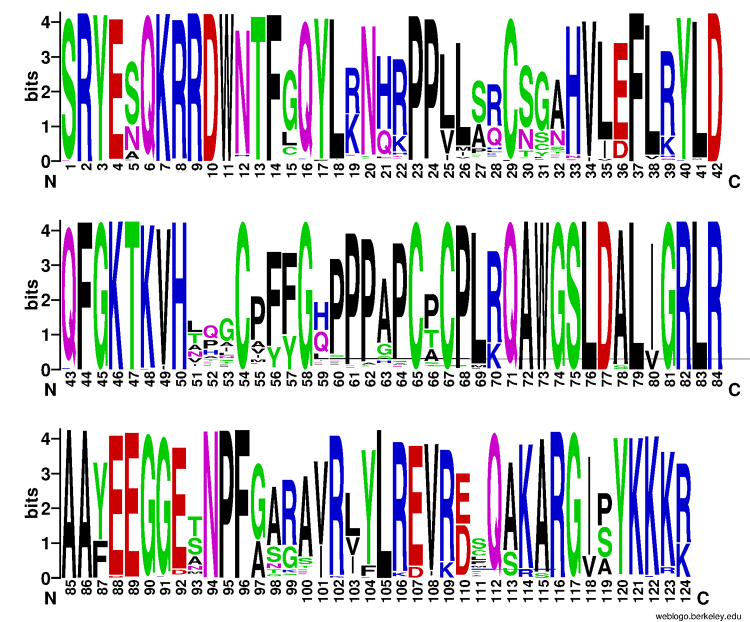
Amino acid sequence logo of ALOG alignments from Streptophyte species (458 sequences). The vertical axis shows the information content of a sequence position. The height of the y-axis is the maximum entropy for a given sequence type. The horizontal axis indicates the residue number. The blue line indicates the SQS_PSY domain.

We used two datasets to perform the phylogenetic analysis of the ALOG gene family. The first dataset (Dataset 1) included 458 sequences from 61 species of the Streptophyte clade to gain insights into the origin and diversification of these genes on land plants. This dataset includes 12 sequences from Charophycean alga, 18 from “Bryophytes” (liverworts, hornworts, and mosses), 23 from lycophytes and Ferns, five from Conifers, 26 from basal angiosperms, 16 from early diverged angiosperms, 11 from Caryophyllales, 73 from monocots, and 274 from eudicots (Rosids and Asterids clades) ( Table S1). The second one (Dataset 2) includes 268 sequences (256 from 20 Solanaceae species, ten sequences from *A. thaliana,* and two from Charophycean algae) to understand better the evolution and function of ALOG genes within the Solanaceae family ( Table S1). The amino acid sequences from both datasets were aligned using MAFFT ( Katoh et al., 2019) with default parameters. The alignments were manually curated using Gblocks 0.91.1 ( https://ngphylogeny.fr/) ( Lemoine et al., 2019). We used the T-coffee package ( Notredame et al., 2000) to check the local reliability of the alignments. Gaps were converted to missing data, and phylogenetic analysis was performed based on the Maximum likelihood method implemented in the IQTree ( Minh et al., 2020) package, with the Parameters -pers 0.2 and -nstop 500, recommended for a dataset with small alignment (Minh *et al.*, 2020). We used 10,0000 ultrafast bootstraps ( Hoang et al., 2018). The ModelFinder ( Kalyaanamoorthy et al., 2017) software implemented in IQTree was used to predict the best-fit model for protein evolution, and the best selected, by BIC criterion, was the Q.plant+R6 for Dataset 1 and Q.plant+G4 for Dataset 2. We generated three independent runs and selected the tree with the better Log-likelihood value. The sequence logo ( Figure 1) was constructed in WebLogo ( Crooks et al., 2004) using the alignment of Dataset 1.

## Results

### Genome-wide identification of ALOG homologs

The BLAST searches against the genomes of 90 Viridiplantae species and one Rhodophyta algae resulted in 701 putative ALOG homologs ( Table S3). As we expected, we did not find ALOG gene sequence in the green algae Chlorophyta (ten species searched) or the Rhodophyta algae (one species searched). After filtering, 648 ALOG sequences from 77 species were considered for the subsequent analyses.

Interestingly, the ALOG genes were not detected in the genome of some Charophycean algae lineages representative of, Mesostigmatophyceae, Chlorokybophyceae, and Klebsormidiophyceae. However, ALOGs were found in others, including Coleochaetophyceae, Charophyceae, Zygnematophyceae (each with one ALOG gene), and Zygnematophyceae (containing three genes in *Spirogloea muscicola* and two in *Zygnema* cf. *cylindricum*). 

Most land plant species have more than one gene for ALOG proteins, except for hornworts, which, together with Charophyceae algae, presented only one ALOG gene ( *Anthoceros agrestis* and *Anthoceros punctatus*). The Liverwort species *Marchantia polymorpha* has two ALOG genes, whereas the mosses species presented two ( *Ceratodon purpureus*), four ( *Physcomitrella patens*), and five ( *Sphagnum fallax* and *Sphagnum magellanicum*) ALOG genes. The Lycophytes species presented two ( *Selaginella moellendorffii*) and five ( *Diphasiastrum complanatum*), and ferns presented three ( *Adiantum capillus*), five ( *Ceratopteris richardii*) and six ( *Alsophila spinulosa*) genes for ALOG proteins, whereas, in the genome of “gymnosperms’’ species, there were found three ( *Thuja plicata*) and one ( *Picea abiens* and *Pinus taeda*) ALOG genes. All angiosperms’ species presented more than four ALOG genes: ANA-grade species with four and five ALOG genes, Magnoliids species with seven and 10 ALOG genes, monocots species with five to 12 ALOG genes, basal eudicots with five to eight ALOG genes, and both eudicots Asterids and eudicots Rosids with eight to 23 ALOG genes. 

Solanaceae ALOG genes were identified by searching with *Arabidopsis* ALOG gene against the genome of 20 species belonging to five genera. A total of 275 genes were identiﬁed in 20 species of Solanaceae, of which 256 remained after ﬁltering steps ( Table S1 and S3). As a result, 11 to 13 ALOG genes were identified in the genome of eight *Solanum* species, 12 to 23 ALOG genes were identified in the genomes of five *Nicotiana* species, seven to 12 ALOG genes were identified in the genome of three *Capsicum* species, 11 and 13 ALOG genes were identified in the genomes of three *Petunia* species (the probably parental species *P. axillaris* and *P. inflata*, and *P. hybrida*), and 10 ALOG genes sequences were identified in the genome of *Datura stramonium*. There were 18 ALOG gene sequences in the genome of allotetraploid *Nicotiana tabacum,* whereas their parental species *N. sylvestris* and *N. tomentosiformis* presented 12 and 14 ALOG genes, respectively. In the same manner, the allopolyploid *N. benthamiana* presented a higher number of ALOG genes (23). 

### Phylogenetic analyses of the ALOG gene family in Streptophyte

**Figure 2 -:**
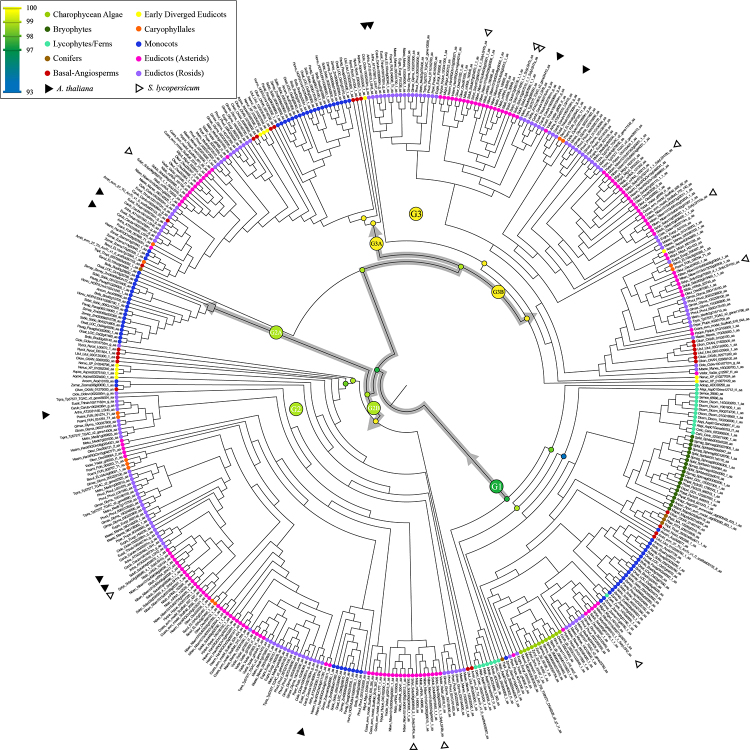
Phylogenetic tree of ALOG gene family in Streptophyte. Maximum likelihood phylogeny of ALOG proteins of representative species of Charophyte algae, Bryophytes, Lycophytes, Ferns, Conifers, basal-angiosperms, Monocots, and eudicots. Node circles represent a branch Ultrafast Bootstrap support value over or equal to 93. Colour circles in the tips of the branches represent the main taxonomic clades represented. Group names, codes, and respective species members are referred to in Table S1.

To better understand the origin and diversification of the ALOG gene family, a phylogenetic tree was inferred with 458 ALOG amino acid sequences spanning 61 species. The alignment used for phylogenetic inference contains 124 sites, representing almost the entirety of the ALOG domain sequence. We observed that the ALOG domain is highly conserved among all species ( Figure 1). The tree topology of ALOG genes was well-supported in most branches with three main groups (G1, G2, and G3) ( Figure 2; Figure S2). G1 represents the ancestral group of ALOG genes, including ALOG homologs from all the taxonomic groups studied (Charophyte algae, “Bryophytes”, lycophytes, ferns, Conifers, basal angiosperms, and monocots with an exception for early diverged angiosperms and Caryophyllales). G2 includes basal angiosperms, early diverged angiosperms, Caryophyllales, monocots, and eudicots. G3 includes one sequence from Conifer, basal angiosperms, early diverged angiosperms, Caryophyllales, monocots, and eudicots. G2 and G3 groups are subdivided into two subgroups each, with a pattern of diversification following the species diversification. The basal angiosperm species are present in the three main clades (G1, G2, and G3), suggesting that the duplication events that promoted the diversification of ALOG started in the ancestral group of angiosperms. Subclade G2A includes only monocot species, while subclade G2B includes basal angiosperms, monocots, and eudicots. The subclade G3A includes all angiosperms studied, while the subclade G3B lacks monocot species ( Figure 2).

### Phylogenetic analyses of ALOG proteins in Solanaceae

**Figure 3 -:**
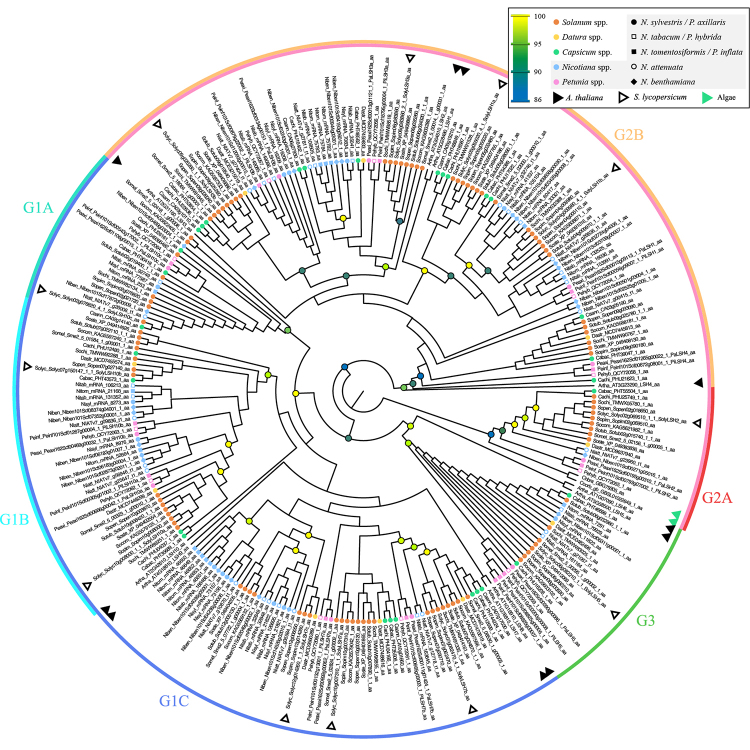
Phylogenetic tree of ALOG gene family in Solanaceae. Maximum likelihood phylogeny of ALOG proteins of Solanaceae species. Node circles represent a branch Ultrafast Bootstrap support value over or equal to 86. Colour circles in the tips of the branches represent the main taxonomic clades represented. Group names, codes, and respective species members are referred to in Table S1.

After filtering, a total of 256 sequences of Solanaceae species were recovered for phylogenetic analyses. The total size of the alignment included 125 sites, covering almost all the ALOG domains. This dataset included ALOG sequences from two charophyte algae and *A. thaliana*. The tree topology generated by Maximum likelihood statistics ( Figure 3; Figure S3) returned three main groups (SG1, SG2, and SG3) with high branch support and representativeness of sequences from different ALOG homologs of Solanaceae species. In general, the ALOG protein followed the genus classification within groups. Among the recovered groups, SG1 was subdivided into three groups. The SG1A group represented SolyLSH10c homologous ALOG sequences from all Solanaceae species, except *Datura stramonium*, and no *A. thaliana* sequences were found in this group; whereas SG1B recovered homologous sequences of SolyLSH10a and SolyLSH10b, closely related to this group was found the *Arabidopsis* AtLSH10 protein. The SG1C group includes ALOG protein homologous of SolyLSH7 (a, b, and c), and closely related to this group, the Arabidopsis AtLSH7, AtLSH8, and AtLSH9 proteins are found. The SG2 group can be further divided into two main groups: SG2A and SG2B. The SG2A recovered the homologous ALOG proteins of Charophycean algae ( *Coleochaete orbicularis* and *Chara braunii*). We did not find Arabidopsis homologous proteins here, but it recovered the Solanum SolyLSH2 protein. Most species were represented in this group, except for one *Capsicum* species ( *C. annuum*) and three *Nicotiana* species (the tobacco crop *N. tabacum* and its parental species *N. sylvestris* and *N. tomentosiformis*). The SG2B included homologous ALOG proteins of *Solanum* SolyLSH1 (a and b) and SolyLSH3 (a and b). This group also contains the *Arabidopsis* AtLSH1, AtLSH2, and AtLSH3 proteins. Moreover, the earliest diverging ALOG protein to SG2B was the *Arabidopsis* AtLSH4 protein. Only *Solanum* and *Capsicum* homologous proteins are present in the group of *Solanum* SolyLSH1a. The remaining SG3 group, even though paraphyletic in its internal relationships, recovered with high support for the homologous protein of *Solanum* SolyLSH5, closely related to *Arabidopsis* AtLSH5 and AtLSH6. These findings regarding the relationships of the ALOG proteins from Solanaceae species and *A. thaliana* could reflect a redundancy of their functions. According to their distribution among species, their duplication events seem to have occurred mainly before the Solanaceae species diversification, but also some paralogous protein sequences were observed showing species-specific duplication events.

Our dataset included three Solanaceae species with a hybrid origin, the diploid *P. hybrida,* the polyploids *N. tabacum* and *N. benthamiana*, and their parental species: *P. axillaris* and *P. inflata* ( *P. hybrida*), and *N. sylvestris* and *N. tomentosiformis* ( *N. tabacum*). A phylogenetic relationship constructed with the proteins of all identified *Petunia* LSH genes shows that 11 of the 13 PhLSH proteins, including PhLSH1, PhLSH2, PhLSH3a, PhLSH3b, PhLSH4, PhLSH5, PhLSH7a, PhLSH7b, PhLSH10a, PhLSH10b, and PhLSH10c, were present in the genome of the three *Petunia* species. *Petunia inflata* included three paralogous protein sequences of PeinfLSH5, not present in *P. axillaris* and *P. hybrid*, showing species-specific duplication events in this species. The phylogenetic relationships revealed that some ALOG proteins of *P. hybrid* came from *P. axillaris* (e.g., PhLSH3a, PhLSH35, PhLSH10a, and PhLSH10b). However, a close relationship between *P. hybrida* and *P. inflata* ALOG protein was not observed (e.g., PLSH1, LSH2, PLSH3b, PLSH4, PLSH7a, PLSH7b, PLSH10c). For five *Nicotiana* species, 12 of 23 LSH proteins were identified, including NLSH1a, NLSH1b, NLSH2, NLSH3a, NLSH3b, NLSH4, NLSH5, NLSH7a, NLSH7b, NLSH7c, NLSH10a, NLSH10b, NLSH10c. The allotetraploid species *N. tabacum* presented 18 ALOG genes, whereas its parental species presented 12 and 14 ALOG genes, respectively, for *N. sylvestris* and *N. tomentosiformis*. Some *N. tabacum* genes of ALOG proteins came from both parental species, however, NLSH3b can only be found in *N. sylvestres* and *N. tabacum,* and the clade of *Solanum* SolyLSH7b homologous proteins, there were only found in *N. tabacum*. An important point to note is that *N. tabacum* did not present all ALOG proteins from its parental species, this is evidenced by its homology to SolyLSH7c. Lastly, the polyploid *N. benthamiana* generally presented more than one gene of ALOG protein related to each homologous *Solanum* gene of ALOG protein.

## Discussion

**Figure 4 -:**
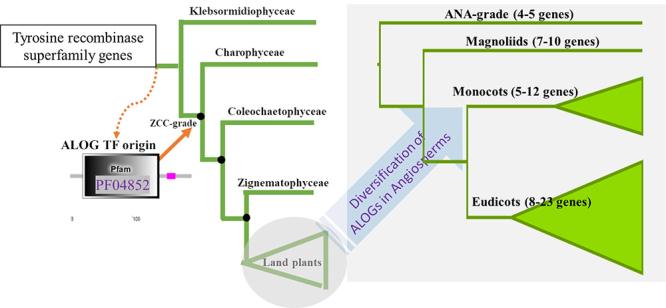
Proposed model of ALOG gene family origin and diversification. The origin of ALOG genes in ZCC-grade and diversification in angiosperm group.

Land plants (embryophytes) evolved from Charophyte algae, and its terrestrialization event was fostered by several evolutionary novelties, including the development of roots and leaves, and the ability to conserve water, among other characteristics ( de Vries *et al*., 2016, de Vries and Archibald, 2017). Additionally, land plants evolved efficient mechanisms of dispersal and reproduction, such as the evolution of seeds and fruits and the extraordinary diversity of floral architecture ( Pires and Dolan, 2012; de Vries and Archibald, 2017). The successful conquest of terrestrial habitats required molecular adaptations, raising new gene families that have played an important role in the diversification of plant species ( Rensing, 2020). Moreover, whole-genome duplication (WGD) events that occurred during plant evolution increased genetic variability, and diversiﬁcation plays an important role in phenotypic innovation ( Qiao et al., 2022). Comparative genomics studies have revealed that many plant genes are conserved across different plant species, indicating that they are functionally important, as, for example, the ALOG gene family presents a conserved domain across land plants ( Chen et al., 2019). Here, we demonstrated that ALOG genes originated in the higher-branching ZCC grade (Zygnematophyceae, Coleochaetophyceae, and Charophyceae) from a Tyrosine recombinase superfamily member ( Figure 4). The origin of ALOG from the N-terminal DNA-binding domains of integrases belonging to the tyrosine recombinase superfamily has already been suggested in a previous study ( Iyer and Aravind, 2012). These proteins are encoded by a distinct type of DIRS1-like LTR retrotransposon and are found in several eukaryotes ( Iyer and Aravind, 2012). 

Previous functional gene characterization studies have shown that the ALOG protein family serves as key developmental regulators in land plants ( Chen et al., 2019; Naramoto et al., 2020), showing their importance as a key innovation for land plant evolution. Until now, the origin of ALOG protein was discussed, and previous studies have claimed that ALOG protein may be exclusive of land plants or rise in green algae ( Zhao et al., 2004; Becker and Marin, 2009; Cho and Zambryski, 2011; Chen *et al.,* 2019; Naramoto *et al*., 2020). Here, we extensively searched for ALOG proteins in the genomes of several green algae (22 green algae species) and one Rhodophyta algae. We did not find any ALOG proteins in Rhodophyta and Chlorophyta algae genomes. However, we found ALOG homologs in species belonging to the ZCC-grade (Zygnematophyceae, Coleochaetophyceae, and Charophyceae) ( Table S1), showing evidence for the origin of ALOG gene family in the ZCC-grade ancestral species, emerging as a transcription factor in the streptophyte lineage of plants.

In general, one ALOG protein was detected in the Charophyceae algae, except for three species from the Zygnematophyceae algae lineage, the closest living relative to land plants ( Hess et al., 2022), presenting three ( *Spirogloea muscicola*) and two ( *Zygnema cf. cylindricum*) ALOG proteins. We suggest that the duplication event that originated the ALOG paralogs in these algae genomes resulted from WGD (whole-genome duplication) since these sequences are grouped in the phylogenetic tree ( Figure 2). The whole-genome triplication (WGT) event has also been suggested for the Zygnematophyceae algae *S. muscicola* with subaerial habitat ( Cheng et al., 2019), which present three ALOG proteins. This same pattern could be inferred for representing early land plants, bryophytes (including hornworts, liverworts, and mosses) that emerged about 460-506 Mya ( Su et al., 2021). Only one ALOG protein was observed in the hornwort genome, whereas mosses showed more than one ALOG protein, where ancient WGD have been unraveled ( Qiao et al., 2022). 

At least in part, the success of angiosperm has been attributed to innovations associated with gene or whole-genome duplications ( de Bodt *et al*., 2005; Soltis et al., 2008; Jiao et al., 2011). Ancient gene duplication events in the common ancestral of extant angiosperm have been suggested (Jiao *et al.*, 2011), resulting in the diversification of important genes to flower development, suggesting their involvement in innovations that contributed to the dominance of angiosperms. Here, we showed that the ALOG genes family has diversified in the basal angiosperms and probably also contributed to the diversity of floral architecture ( Figure 4, MacAlister et al., 2012; Žádníková and Simon, 2014; Zhang and Yuan, 2014; Xiao et al., 2019; He *et al*., 2020). Our analysis showed that several rounds of duplication marked the evolution of ALOG genes family, with duplication events occurring after angiosperm emergence. The phylogenetic tree of ALOG genes in streptophyte species revealed both ancient and more recent duplication events. Most of these duplication events are related to WGD. 

Our phylogenetic results showed that the highest diversification of ALOG family of proteins was in the flowering plants ( Figure 4). The Solanaceae ALOG phylogeny also suggests that ancestral duplication events could occur in the evolution of ALOG sequences in this family, as events of WGD have been suggested in the ancestral of this family ( The Tomato Genome Consortium, 2012). These duplication events may be related to differential spatial patterns of gene expression as observed for homologous PhLSH3a and PhLSH3b, which showed the highest expression in inflorescences and flower buds, and PhLSH10a and PhLSH10b that showed higher expression patterns in buds followed by stem (PhLSH10a), and mainly expressed in roots, stems, seedling, and fruits (PhLSH10b) ( Chen et al., 2019). Interestingly, the paralogous PhLSH10b was only expressed in roots (Chen *et al.*, 2019). We observed for the homologous NLSH3a in the diploid parental species of tobacco crop, *N. tomentosiformis*, two paralogous ALOG sequences that were closely related to *N. tabacum* (Nitab NLSH3a), and only one homologous NLSH3a in the *N. sylvestris*, also grouped with *N. tabacum*. Interestingly for the LSH3b homologs, our results showed a group well-supported containing only *Capsicum*, *Solanum,* and *Datura* species and a paraphyletic group containing *Petunia* and *Nicotiana* species, representing the similarity of these sequences suggesting their similar function. For homologous LSH10a and LSH10b, the sequences are grouped by genus.

Our Solanaceae phylogeny also showed evidence for species-specific duplication events. For example, the homologous sequence for LSH5 in *Petunia inflata* with three paralogs with high similarity in their protein sequences is absent in the *P. axillaris* and *P. hybrid.* These sequences were grouped with high support with homologous of AtLSH5 and AtLSH6. This group presents a well-supported subgroup of *Petunia* protein sequences and another paraphyletic group with *Solanum*, *Nicotiana*, *Capsicum,* and *Datura* protein sequences. Chen et al. (2019) showed that this gene was higher expressed in the stem and root of *P. hybrida*. *Petunia* genus encompasses ~15 recently diverged diploid species, and the first split on molecular species phylogeny supports two main clades related to flower phenotype, purple and short-tube length vs. a variety of colour and long-tube of corolla ( Reck-Kortmann et al., 2014). These traits were related to the main functional pollinator contributing to species diversity ( Fregonezi et al., 2013). Moreover, these two *Petunia* species grow in different environmental conditions, such as altitude, climate, and soil traits ( Lorenz-Lemke et al., 2010; Barros et al., 2015; Pezzi et al., 2022). If this duplication event on homologs of the LSH5 gene in *P. inflata* is a species-specific event or at the base of the ancestral of this *Petunia* clade needs to be investigated.

Interestingly, the diversification of homologous SoliLSH7c in *Nicotiana* genus, even in diploid species such as *N. sylvestris* (four paralogs) and *N. tomentosiformis* (five paralogs). This ALOG protein was not present in *Petunia* but belongs to a clade that included PhLSH7a and PhLSH7a and is closely related to AtLSH7/8. Previous studies have shown that LSH7 homologous play significant roles in plant growth and development, as in root and fruit development in *Petunia* and vegetative growth and fertility in *Arabidopsis* ( Chen et al., 2019). The presence of multiple copies of a gene can allow for different regulatory elements to evolve, resulting in changes in the timing, location, or level of gene expression. This finding raises the hypothesis of functional diversification of these ALOG genes in *Nicotiana* that needs to be tested.

Our results showed that the *P. hybrida* ALOG protein came especially from *P. axillaris*, as previously identified by Chen et al. (2019). These authors also suggest that some ALOG protein of *P. hybrida* was most similar to *P. exserta* ALOG sequences (e.g., PhLSH3a and PhLSH5). *Petunia hybrida* originates from experimental crosses between the wild white-ﬂowered *Petunia axillaris* and individuals of purple-ﬂowered species, like *P. inflata* ( Bombarely et al., 2016), but Segatto et al. (2013) have suggested the *P. interior*, closely related species to *P. inflata*, as the purple-flowered wild parental of *P. hybrida*. Polyploidy influences the genome structure and has played an important role in the evolution of gene families, allowing the expansion and diversification of gene functions ( Soltis et al., 2008; Jiao et al., 2011). The hybrid origin, especially with genome duplication as in allopolyploids, influences the genome structure and gene retention or loss. This is the case for almost half of *Nicotiana* species, originating from ancient hybridization events and, subsequently, diversification and loss of chromosome number, as in *N. benthaminana* ( Knapp, 2020).

In summary, our results bring evidence for the origin of ALOG proteins in closely related to land plants algae lineages, and the ancestral genome duplication events increase the ALOG genes in land plants lineages, and most diversification occurred in flowering plants, suggesting their potential role on floral development and phenotypic diversification. In Solanaceae family, ALOG proteins relationship most follow the taxonomic classification of the genus within each clade. We also could observe probable species-specific duplication events in Solanaceae, suggestion or the redundant function or spatial-temporal gene expression, as observed for *Petunia*. Adding more representative species of Solanaceae and functional studies could give more in-depth knowledge of this gene family’s evolution and function.

## Supplementary material

The following online material is available for this article:

Table S1 - Information regarding plant species genomes searched for ALOG genes, database searched, and total number of genes found for each species.

Table S2 - BLAST test with all *Arabisopsis thaliana* ALOG (AtLSH1-10) protein sequences against the genome of *Chara brawnii* algae, the “bryophytes” *Marchantia polymorpha* and *Physcomitrella patens*, the “gymnosperm” *Picea abiens*, the Eudicot Rosids *Arabidopsis thaliana*, the Eudicot Asterids *Solanum lycopersicum* and the monocot *Oryza sativa*.

Table S3 - Information regarding BLAST statistics for each target species selected for this study.

Figure S1 - Schematic view of the filtering steps and analyses of ALOG genes in this study.

Figure S2 - Phylogenetic tree of ALOG gene family in Streptophyte showing the branches support.

Figure S3 - Phylogenetic tree of ALOG gene family in Solanaceae showing the branches support.
