# Supplementary material for: Genome-wide identification and evolutionary view of ALOG gene family in Solanaceae
Source: Genet Mol Biol. 2023 Dec 1;46(3 Suppl 1):e20230142. doi: 10.1590/1415-4757-GMB-2023-0142 (PMC10695626; doi:10.1590/1415-4757-GMB-2023-0142)
Supplement: Table S1 - [file 1415-4757-GMB-46-3-s1-e20230142-s1.pdf]

Genetics and Molecular Biology

https://doi.org/10.1590/1678-4685-GMB-2023-0142

Supplementary Material to “Genome-wide identification and evolutionary view of ALOG gene family in Solanaceae”

Table S1 – Information regarding plant species genomes searched for ALOG genes, database searched, and total number of genes found for each species. * Solanaceae species used in Dataset 1.

Acronym	Species	Clade/Division or Lineages	Order/Family	Reference	N hits
Poumb	*Porphyra umbilicalis*	Rhodophyta	Bangiaceae	Brawley et al. 2017	No hits found
Osluc	*Ostreococcus lucimarinus*	Chlorophyta	Bathycoccaceae	Palenik et al. 2007	No hits found
Ostau	*Ostreococcus tauri*	Chlorophyta	Bathycoccaceae	Blanc-Mathieu et al. 2014	No hits found
Bobra	*Botryococcus braunii*	Chlorophyta	Botryococcaceae	Browne et al. 2017	No hits found
Chrei	*Chlamydomonas reinhardtii*	Chlorophyta	Chlamydomonadaceae	Shrager et al. 2003	No hits found
Chzof	*Chromochloris zofingiensis*	Chlorophyta	Chromochloridaceae	Roth et al. 2017	No hits found
Dusal	*Dunaliella salina*	Chlorophyta	Dunaliellaceae	Polle et al. 2017	No hits found

**Acronym**	**Species**	**Clade/Division or Lineages**	**Order/Family**	**Reference**	**N hits**
Mipus	*Micromonas pusilla*	Chlorophyta	Mamiellaceae	van Baren et al. 2016	No hits found
Misp.	*Micromonas sp.*	Chlorophyta	Mamiellaceae		No hits found
Cosub	*Coccomyxa subellipsoidea*	Chlorophyta	Coccomyxaceae	Blanc et al. 2012	No hits found
Vocar	*Volvox carteri*	Chlorophyta	Volvocaceae	Prochnik et al. 2010	No hits found
Mevir	*Mesostigma viride*	Streptophyta/Charophyta/Mesostigmatophyceae	Mesostigmales/Mesostigmataceae	Wang et al. 2020	No hits found
Chatm	*Chlorokybus atmophyticus*	Streptophyta/Charophyta/Chlorokybophyceae	Chlorokybales/Chlorokybophyceae	Wang et al. 2020	No hits found
Klnit	*Klebsormidium nitens*	Streptophyta/Charophyta/Klebsormidiophyceae	Klebsormidiales/Klebsormidiaceae	Hori et al. 2014	No hits found
**Coorb**	*Coleochaete orbicularis*	Streptophyta/Charophyta/Coleochaetophyceae	Coleochaetales/Coleochaetaceae	Sablok et al. 2019	1
**Nimir**	*Nitella mirabilis*	Streptophyta/Charophyta/Charophyceae	Charales/Characeae		1
**Chbra**	*Chara braunii*	Streptophyta/Charophyta/ Charophyceae	Charales/Characeae	Nishiyama et al. 2018	1
**Sppra**	*Spirogyra pratensis*	Streptophyta/Charophyta/ Zygnematophyceae	Zygnematales/Zygnemataceae		1

Acronym	Species	Clade/Division or Lineages	Order/Family	Reference	N hits
**Spmus**	*Spirogloea muscicola*	Streptophyta/Charophyta/ Zygnematophyceae	Spirogloeales/Spirogloeaceae		3
**Meend**	*Mesotaenium endlicherianum SAG 12.97*	Streptophyta/Charophyta/ Zygnematophyceae	Zygnematales/Mesotaeniacea	Cheng et al. 2019	1
**Mekra**	*Mesotaenium kramstae Lemmermann*	Streptophyta/Charophyta/ Zygnematophyceae	Zygnematales/Mesotaeniacea		1
**Zycyl**	*Zygnema cf. cylindricum SAG 698-1a*	Streptophyta/Charophyta/ Zygnematophyceae	Zygnematales/Zygnemataceae		2
**Zycir SAG698**	*Zygnema circumcarinatum SAG 698-1b*	Streptophyta/Charophyta/ Zygnematophyceae	Zygnematales/Zygnemataceae		1
**Mapol**	*Marchantia polymorpha*	Embriophyta/Marchantiophyta	Marchantiales/Marchantiaceae	Bowman et al. 2017	2
**Cepur**	*Ceratodon purpureus*	Embriophyta/Bryophyta	Dicranales/Ditrichaceae		2
**Phpat**	*Physcomitrella patens*	Embriophyta/Bryophyta	Funariales/Funariaceae	Lang et al. 2018	4
**Spfal**	*Sphagnum fallax*	Embriophyta/Bryophyta	Sphagnales/Sphagnaceae		5
**Spmag**	*Sphagnum magellanicum*	Embriophyta/Bryophyta	Sphagnales/Sphagnaceae		5

Acronym	Species	Clade/Division or Lineages	Order/Family	Reference	N hits
**Anagr**	*Anthoceros agrestis*	Embriophyta/Anthocerophyta	Anthocerotales/Anthocerotaceae	Li et al. 2020	1
**Anpun**	*Anthoceros punctatus*	Embriophyta/Antocerophyta	Anthocerotales/Anthocerotaceae	Li et al. 2020	1
**Semoe**	*Selaginella moellendorffii*	Tracheophyta/Lycopodiophyta	Selaginellales/Selaginellaceae	Banks et al. 2011	2
**Dicom**	*Diphasiastrum complanatum*	Tracheophyta/Lycopodiophyta	Lycopodiales/Lycopodiaceae		5
**Ceric**	*Ceratopteris richardii*	Tracheophyta/Monilophyta	Polypodiales/Pteridaceae	Marchant et al. 2019	5
**Adcap**	*Adiantum capillus*	Tracheophyta/Monilophyta	Polypodiales/Pteridaceae	Fang et al. 2022	3
**Alspi**	*Alsophila spinulosa*	Tracheophyta/Monilophyta	Cyatheales/Cyatheaceae	Huang et al. 2022	6
**Thpli**	*Thuja plicata*	Spermatophyta/Gymnosperm	Pinales/Cupressaceae	See Wan et al. 2023	3
**Piabi**	*Picea abiens*	Spermatophyta/Gymnosperm	Pinales/Pinaceae	See Wan et al. 2024	1
**Pitae**	*Pinus taeda*	Spermatophyta/Gymnosperm	Pinales/Pinaceae	See Wan et al. 2025	1
**Amtri**	*Amborella trichopoda*	Spermatophyta/Angiosperm (ANA-grade)	Amborellales/Amborellaceae	Amborella Genome Project 2013	4

Acronym	Species	Clade/Division or Lineages	Order/Family	Reference	N hits
**Nycol**	*Nymphaea colorata*	Spermatophyta/Angiosperm (ANA-grade)	Nymphaeales/Nymphaeaceae	Zhang et al. 2019	5
**Litul**	*Liriodendron tulipifera*	Spermatophyta/Angiosperm (Magnoliids)	Magnoliales/Magnoliaceae	Chen et al. 2019	7
**Cikan**	*Cinnamomum kanehirae*	Spermatophyta/Angiosperm (Magnoliids)	Laurales/Lauraceae	Chaw et al. 2019	10
**Nenuc**	*Nelumbo nucifera*	Spermatophyta/Angiosperm (Basal Eudicot)	Proteales/ Nelumbonaceae	Ming et al. 2013	6
**Aqcoe**	*Aquilegia coerulea*	Spermatophyta/Angiosperm (Basal Eudicot)	Ranunculales/Ranunculaceae	Filiault et al. 2018	5
**Poami**	*Portulaca amilis*	Spermatophyta/Angiosperm (Eudicot)	Caryophyllales/Portulacaceae		8
**Bevul**	*Beta vulgaris*	Spermatophyta/Angiosperm (Eudicot)	Caryophyllales/Amaranthaceae	Dhom et al. 2013	8
**Zomar**	*Zostera marina*	Spermatophyta/Angiosperm (Monocot)	Alismatales/Zosteraceae	Olsen et al. 2016	9
**Ancom**	*Ananas comosus*	Spermatophyta/Angiosperm (Monocot)	Poales/Bromeliaceae	Ming et al. 2015	5
**Brdis**	*Brachypodium distachyon*	Spermatophyta/Angiosperm (Monocot)	Poales/Poaceae	The International Brachypodium Initiative 2010	10
**Hovul**	*Hordeum vulgare*	Spermatophyta/Angiosperm (Monocot)	Poales/Poaceae	Beier et al. 2017	6

Acronym	Species	Clade/Division or Lineages	Order/Family	Reference	N hits
**Orsat**	*Oryza sativa*	Spermatophyta/Angiosperm (Monocot)	Poales/Poaceae		10
**Sobic**	*Sorghum bicolor*	Spermatophyta/Angiosperm (Monocot)	Poales/Poaceae	Cooper et al. 2019	12
**Zemay**	*Zea mays*	Spermatophyta/Angiosperm (Monocot)	Poales/Poaceae	Sun et al. 2022	11
**Pavag**	*Paspalum vaginatum*	Spermatophyta/Angiosperm (Monocot)	Poales/Poaceae		10
**Hyque**	*Hydrangea quercifolia*	Spermatophyta/Angiosperm (Eudicot/Asterid)	Cornales/Hydrangeaceae		8
**Vadar**	*Vaccinium darrowii*	Spermatophyta/Angiosperm (Eudicot/Asterid)	Ericales/Ericaceae	Yu et al. 2021	13
**Heann**	*Helianthus annuus*	Spermatophyta/Angiosperm (Eudicot/Asterid)	Asterales/Asteraceae	Badouin et al. 2017	13
**Migut**	*Mimulus guttatus*	Spermatophyta/Angiosperm (Eudicot/Asterid)	Lamiales/Phrymaceae		8
**Oleur**	*Olea europaea*	Spermatophyta/Angiosperm (Eudicot/Asterid)	Lamiales/Oleaceae	Rao et al. 2021	14
**Coara**	*Coffea arabica*	Spermatophyta/Angiosperm (Eudicot/Asterid)	Gentianales/Rubiaceae	Mekbib et al. 2022	12
**Caann**	*Capsicum annuum*	Spermatophyta/Angiosperm (Eudicot/Asterid)	Solanales/Solanaceae	Kim et al. 2014	7

Acronym	Species	Clade/Division or Lineages	Order/Family	Reference	N hits
**Cabac**	*Capsicum baccatum*	Spermatophyta/Angiosperm (Eudicot/Asterid)	Solanales/Solanaceae	Ahn et al. 2018	12
**Cachi**	*Capsicum chinense*	Spermatophyta/Angiosperm (Eudicot/Asterid)	Solanales/Solanaceae	Kim et al. 2014	12
**Dastr**	*Datura stramonium*	Spermatophyta/Angiosperm (Eudicot/Asterid)	Solanales/Solanaceae	Rajewski et al. 2021	10
**Niatt**	*Nicotiana attenuata*	Spermatophyta/Angiosperm (Eudicot/Asterid)	Solanales/Solanaceae	Xu et al. 2017	14
**Niben***	*Nicotiana benthamiana*	Spermatophyta/Angiosperm (Eudicot/Asterid)	Solanales/Solanaceae	Kurotani et al. 2023	23
**Nisyl**	*Nicotiana sylvestris*	Spermatophyta/Angiosperm (Eudicot/Asterid)	Solanales/Solanaceae	Sierro et al. 2013	12
**Nitab***	*Nicotiana tabacum*	Spermatophyta/Angiosperm (Eudicot/Asterid)	Solanales/Solanaceae	Edwards et al. 2017	18
**Nitom**	*Nicotiana tomentosiformis*	Spermatophyta/Angiosperm (Eudicot/Asterid)	Solanales/Solanaceae	Sierro et al. 2013	14
**Peaxi**	*Petunia axillaris*	Spermatophyta/Angiosperm (Eudicot/Asterid)	Solanales/Solanaceae	Bombarely et al. 2016	11
**Pehyb**	*Petunia hybrida*	Spermatophyta/Angiosperm (Eudicot/Asterid)	Solanales/Solanaceae		11
**Peinf**	*Petunia inflata*	Spermatophyta/Angiosperm (Eudicot/Asterid)	Solanales/Solanaceae	Bombarely et al. 2016	13

Acronym	Species	Clade/Division or Lineages	Order/Family	Reference	N hits
**Sochi**	*Solanum chilense*	Spermatophyta/Angiosperm (Eudicot/Asterid)	Solanales/Solanaceae	Stam et al. 2019	12
**Socom**	*Solanum commersonii*	Spermatophyta/Angiosperm (Eudicot/Asterid)	Solanales/Solanaceae	Aversano et al. 2015	13
**Solyc***	*Solanum lycopersicum*	Spermatophyta/Angiosperm (Eudicot/Asterid)	Solanales/Solanaceae	The Tomato Genome Consortium	12
**Somel**	*Solanum melongena*	Spermatophyta/Angiosperm (Eudicot/Asterid)	Solanales/Solanaceae	Wei et al. 2020	11
**Sopen**	*Solanum pennellii*	Spermatophyta/Angiosperm (Eudicot/Asterid)	Solanales/Solanaceae	Bolger et al. 2014	13
**Sopim**	*Solanum pimpinellifollium*	Spermatophyta/Angiosperm (Eudicot/Asterid)	Solanales/Solanaceae	Wang et al 2020	12
**Soste**	*Solanum stenotomum*	Spermatophyta/Angiosperm (Eudicot/Asterid)	Solanales/Solanaceae	Yan et al. 2021	13
**Sotub***	*Solanum tuberosum*	Spermatophyta/Angiosperm (Eudicot/Asterid)	Solanales/Solanaceae	The Potato Genome Sequencing Consortium	13
**Artha**	*Arabidopsis thaliana*	Spermatophyta/Angiosperm (Eudicot/Rosid)	Brassicales/Brassicaceae		10
**Carub**	*Capsella rubella*	Spermatophyta/Angiosperm (Eudicot/Rosid)	Brassicales/Brassicaceae		11
**Eusal**	*Eutrema salsugineum*	Spermatophyta/Angiosperm (Eudicot/Rosid)	Brassicales/Brassicaceae		10

Acronym	Species	Clade/Division or Lineages	Order/Family	Reference	N hits
**Glmax**	*Glycine max*	Spermatophyta/Angiosperm (Eudicot/Rosid)	Fabales/Fabaceae/Papilionoideae	Schmutz et al. 2010	23
**Metru**	*Medicago truncatula*	Spermatophyta/Angiosperm (Eudicot/Rosid)	Fabales/Fabaceae/Papilionoideae	Pecrix et al. 2018	14
**Phvul**	*Phaseolus vulgaris*	Spermatophyta/Angiosperm (Eudicot/Rosid)	Fabales/Fabaceae/Papilionoideae	Schmutz et al. 2014	16
**Trpra**	*Trifolium pratense*	Spermatophyta/Angiosperm (Eudicot/Rosid)	Fabales/Fabaceae/Papilionoideae	de Veja et al. 2015	12
**Eugra**	*Eucalyptus grandis*	Spermatophyta/Angiosperm (Eudicot/Rosid)	Myrtales/Myrtaceae	Myburg et al. 2014	11
**Prper**	*Prunus persica*	Spermatophyta/Angiosperm (Eudicot/Rosid)	Rosales/Rosaceae	Verde et al. 2013	8
**Cicle**	*Citrus clementina*	Spermatophyta/Angiosperm (Eudicot/Rosid)	Sapindales/Rutaceae	Wu et al. 2014	9
**Maesc**	*Manihot esculenta*	Spermatophyta/Angiosperm (Eudicot/Rosid)	Malpighiales/Euphorbiaceae	Bredeson et al. 2016	16
