# Supplementary material for: Genome-wide identification and evolutionary view of ALOG gene family in Solanaceae
Source: Genet Mol Biol. 2023 Dec 1;46(3 Suppl 1):e20230142. doi: 10.1590/1415-4757-GMB-2023-0142 (PMC10695626; doi:10.1590/1415-4757-GMB-2023-0142)
Supplement: Table S2 - [file 1415-4757-GMB-46-3-s1-e20230142-s2.pdf]

Genetics and Molecular Biology

https://doi.org/10.1590/1678-4685-GMB-2023-0142

Supplementary Material to "Genome-wide identification and evolutionary view of ALOG gene family in Solanaceae"

Table S2 – BLAST test with all *Arabisopsis thaliana* ALOG (AtLSH1-10) protein sequences against the genome of *Chara brawnii* algae, the "bryophytes" *Marchantia polymorpha* and *Physcomitrella patens*, the "gymnosperm" *Picea abiens*, the Eudicot Rosids *Arabidopsis thaliana*, the Eudicot Asterids *Solanum lycopersicum* and the monocot *Oryza sativa*.

QUERY (Access Code)	Target Species	Gene Name	Access Code	Score	E-Value	Identity %
LSH1 (AT5G28490.1)	*Chara brawnii*		GBG79305	228	9.30E-24	67
	Marchantia polymorpha	MpLOS1	Mapoly0028s0118.1	210	2.37E-69	81
	Marchantia polymorpha	MpLOS2	Mapoly0221s0004.1	193	9.82E-63	69
	Physcomitrella patens		Pp3c8_6310V3.1	208	8.30E-66	71
	Physcomitrella patens		Pp3c20_17990V3.1	208	1.40E-65	80
	Physcomitrella patens		Pp3c24_8490V3.1	206	7.50E-65	80
	Physcomitrella patens		Pp3c23_9660V3.1	203	1.00E-63	79
	Picea abiens		MA_12926g0010	210	2.37E-69	81

QUERY (Access Code)	Target Species	Gene Name	Access Code	Score	E-Value	Identity %
	Picea abiens		MA_211369g0010	193	9.82E-63	69
	Picea abiens		MA_139833g0010	135	6.99E-40	55
	Arabidopsis thaliana	LSH1	AT5G28490.1	320	4.75E-113	100
	Arabidopsis thaliana	LSH4	AT3G23290.2	210	1.12E-69	87
	Arabidopsis thaliana	LSH3	AT2G31160.1	215	3.31E-71	85
	Arabidopsis thaliana	LSH2	AT3G04510.1	250	2.68E-85	84
	Arabidopsis thaliana	LSH5	AT5G58500.1	192	9.72E-63	73
	Arabidopsis thaliana	LSH10	AT2G42610.1	189	2.10E-61	72
	Arabidopsis thaliana	LSH6	AT1G07090.1	205	2.63E-67	70
	Arabidopsis thaliana	LSH7	AT1G78815.1	183	5.43E-59	70
	Arabidopsis thaliana	LSH9	AT4G18610.1	169	1.30E-53	70
	Arabidopsis thaliana	LSH8	AT1G16910.1	176	1.98E-56	65
	Oryza sativa	OsG1L9	LOC_Os05g28040.1 (284: 53-180)	214	3.00E-68	82
	Oryza sativa	OsG1L7	LOC_Os01g61310.1	211	4.80E-68	80
	Oryza sativa	OsG1L3	LOC_Os02g41460.1	209	2.70E-67	81
	Oryza sativa	OsG1L6 (TH1/BH1/AFD1)	LOC_Os02g56610.1	209	6.90E-67	82

QUERY (Access Code)	Target Species	Gene Name	Access Code	Score	E-Value	Identity %
	Oryza sativa	OsG1L1	LOC_Os02g07030.1	208	2.70E-66	71
	Oryza sativa	OsG1L2	LOC_Os06g46030.1	208	3.40E-66	81
	Oryza sativa	OsG1L5 (TAWAWA1)	LOC_Os10g33780.1	204	1.40E-65	86
	Oryza sativa	OsG1L4	LOC_Os04g43580.2	204	2.30E-65	82
	Oryza sativa	OsG1L8	LOC_Os05g39500.1	204	3.80E-65	78
	Oryza sativa	OsG1	LOC_Os07g04670.1	144	1.30E-41	55
	Solanum lycopersicum	SolyLSH1a	Solyc05g055020.4.1	259	4.00E-88	83
	Solanum lycopersicum	TMF	Solyc09g090180.1.1	221	6.00E-73	86
	Solanum lycopersicum	SolyLSH3b	Solyc09g025280.1.1	215	2.00E-71	85
	Solanum lycopersicum	SolyLSH5	Solyc06g082210.1.1	211	6.00E-69	74
	Solanum lycopersicum	SolyLSH1b	Solyc04g009980.4.1	239	8.00E-81	62
	Solanum lycopersicum	SolyLSH3a	Solyc06g083860.2.1	207	2.00E-67	86
	Solanum lycopersicum	SolyLSH2	Solyc02g069510.1.1	225	3.00E-75	76
	Solanum lycopersicum	SolyLSH7c	Solyc12g014260.1.1	212	1.00E-69	76
	Solanum lycopersicum	SolyLSH10b	Solyc07g150147.1.1	212	1.00E-69	75
	Solanum lycopersicum	SolyLSH7a	Solyc10g007310.1.1	210	7.00E-69	75
	Solanum lycopersicum	SolyLSH10a	Solyc10g008000.1.1	210	4.00E-69	72

**QUERY (Access Code)**	**Target Species**	**Gene Name**	**Access Code**	**Score**	**E-Value**	**Identity %**
	Solanum lycopersicum	SolyLSH10c	Solyc02g076820.4.1	207	1.00E-67	75
	Solanum lycopersicum	SolyLSH7b	Solyc07g062470.4.1	186	1.00E-59	64
LSH2 (AT3G04510.1)	*Chara brawnii*		GBG79305	227	8.70E-24	65
	Marchantia polymorpha	MpLOS1	Mapoly0028s0118.1.p	205	1.58E-53	78
	Marchantia polymorpha	MpLOS2	Mapoly0221s0004.1.p	174	1.36E-65	66
	Physcomitrella patens		Pp3c8_6310V3.1.p	203	1.93E-64	77
	Physcomitrella patens		Pp3c20_17990V3.1.p	203	9.12E-65	78
	Physcomitrella patens		Pp3c24_8490V3.1.p	200	1.18E-63	77
	Physcomitrella patens		Pp3c23_9660V3.1.p	199	5.53E-63	76
	Picea abiens		MA_12926g0010	203	1.04E-66	76
	Picea abiens		MA_211369g0010	183	7.21E-59	65
	Picea abiens		MA_139833g0010	131	2.06E-38	74
	Arabidopsis thaliana	LSH1	AT5G28490.1	247	5.94E-84	81
	Arabidopsis thaliana	LSH4	AT3G23290.2	204	7.40E-67	83
	Arabidopsis thaliana	LSH3	AT2G31160.1	209	1.80E-68	82
	Arabidopsis thaliana	LSH2	AT3G04510.1	305	7.40E-107	100

QUERY (Access Code)	Target Species	Gene Name	Access Code	Score	E-Value	Identity %
	Arabidopsis thaliana	LSH5	AT5G58500.1	190	1.91E-61	71
	Arabidopsis thaliana	LSH10	AT2G42610.1	184	1.33E-59	73
	Arabidopsis thaliana	LSH6	AT1G07090.1	198	1.20E-64	72
	Arabidopsis thaliana	LSH7	AT1G78815.1	182	1.82E-58	69
	Arabidopsis thaliana	LSH9	AT4G18610.1	172	2.49E-54	70
	Arabidopsis thaliana	LSH8	AT1G16910.1	170	4.11E-54	66
	Oryza sativa	OsG1L9	LOC_Os05g28040.1	206	4.30E-65	78
	Oryza sativa	OsG1L7	LOC_Os01g61310.1	207	3.10E-66	78
	Oryza sativa	OsG1L3	LOC_Os02g41460.1	199	2.20E-63	78
	Oryza sativa	OsG1L6 (TH1/BH1/AFD1)	LOC_Os02g56610.1	202	7.10E-64	78
	Oryza sativa	OsG1L1	LOC_Os02g07030.1	196	2.30E-61	75
	Oryza sativa	OsG1L2	LOC_Os06g46030.1	197	8.90E-62	77
	Oryza sativa	OsG1L5 (TAWAWA1)	LOC_Os10g33780.1	198	3.80E-63	82
	Oryza sativa	OsG1L4	LOC_Os04g43580.2	194	1.60E-61	80
	Oryza sativa	OsG1L8	LOC_Os05g39500.1	202	6.10E-64	76
	Oryza sativa	OsG1	LOC_Os07g04670.1	140	1.30E-39	52

QUERY (Access Code)	Target Species	Gene Name	Access Code	Score	E-Value	Identity %
	Solanum lycopersicum	SolyLSH1a	Solyc05g055020.4.1	256	1.00E-86	85
	Solanum lycopersicum	TMF	Solyc09g090180.1.1	237	1.00E-79	72
	Solanum lycopersicum	SolyLSH3b	Solyc09g025280.1.1	223	3.00E-74	72
	Solanum lycopersicum	SolyLSH5	Solyc06g082210.1.1	216	4.00E-71	81
	Solanum lycopersicum	SolyLSH1b	Solyc04g009980.4.1	212	2.00E-69	73
	Solanum lycopersicum	SolyLSH3a	Solyc06g083860.2.1	211	5.00E-69	73
	Solanum lycopersicum	SolyLSH2	Solyc02g069510.1.1	208	2.00E-68	81
	Solanum lycopersicum	SolyLSH7c	Solyc12g014260.1.1	209	3.00E-68	73
	Solanum lycopersicum	SolyLSH10b	Solyc07g150147.1.1	209	4.00E-68	59
	Solanum lycopersicum	SolyLSH7a	Solyc10g007310.1.1	208	9.00E-68	72
	Solanum lycopersicum	SolyLSH10a	Solyc10g008000.1.1	206	2.00E-67	69
	Solanum lycopersicum	SolyLSH10c	Solyc02g076820.4.1	202	3.00E-65	82
	Solanum lycopersicum	SolyLSH7b	Solyc07g062470.4.1	185	4.00E-59	64
LSH3 (AT2G31160.1)	*Chara brawnii*		GBG79305	240	2.50E-25	73
	Marchantia polymorpha	MpLOS1	Mapoly0028s0118.1.p	232	1.28E-75	87
	Marchantia polymorpha	MpLOS2	Mapoly0221s0004.1.p	188	3.81E-58	73

**QUERY (Access Code)**	**Target Species**	**Gene Name**	**Access Code**	**Score**	**E-Value**	**Identity %**
	Physcomitrella patens		Pp3c8_6310V3.1.p	221	2.13E-71	84
	Physcomitrella patens		Pp3c20_17990V3.1.p	222	1.09E-71	85
	Physcomitrella patens		Pp3c24_8490V3.1.p	221	1.38E-71	84
	Physcomitrella patens		Pp3c23_9660V3.1.p	219	1.55E-70	84
	Picea abiens		MA_12926g0010	227	7.32E-76	86
	Picea abiens		MA_211369g0010	207	9.26E-68	76
	Picea abiens		MA_139833g0010	146	1.10E-43	84
	Arabidopsis thaliana	LSH1	AT5G28490.1	214	8.39E-71	85
	Arabidopsis thaliana	LSH4	AT3G23290.2	229	2.12E-76	93
	Arabidopsis thaliana	LSH3	AT2G31160.1	353	3.77E-125	100
	Arabidopsis thaliana	LSH2	AT3G04510.1	209	1.40E-68	82
	Arabidopsis thaliana	LSH5	AT5G58500.1	208	1.71E-68	78
	Arabidopsis thaliana	LSH10	AT2G42610.1	195	2.18E-63	75
	Arabidopsis thaliana	LSH6	AT1G07090.1	220	4.39E-73	78
	Arabidopsis thaliana	LSH7	AT1G78815.1	178	1.21E-56	69
	Arabidopsis thaliana	LSH9	AT4G18610.1	163	9.04E-51	67
	Arabidopsis thaliana	LSH8	AT1G16910.1	159	1.51E-49	63

QUERY (Access Code)	Target Species	Gene Name	Access Code	Score	E-Value	Identity %
	Oryza sativa	OsG1L9	LOC_Os05g28040.1	207	3.20E-65	78
	Oryza sativa	OsG1L7	LOC_Os01g61310.1	218	2.00E-70	83
	Oryza sativa	OsG1L3	LOC_Os02g41460.1	227	1.10E-73	86
	Oryza sativa	OsG1L6 (TH1/BH1/AFD1)	LOC_Os02g56610.1	233	1.40E-75	89
	Oryza sativa	OsG1L1	LOC_Os02g07030.1	223	2.40E-71	85
	Oryza sativa	OsG1L2	LOC_Os06g46030.1	221	1.40E-70	86
	Oryza sativa	OsG1L5 (TAWAWA1)	LOC_Os10g33780.1	216	1.30E-69	87
	Oryza sativa	OsG1L4	LOC_Os04g43580.2	214	5.40E-69	87
	Oryza sativa	OsG1L8	LOC_Os05g39500.1	212	1.20E-67	81
	Oryza sativa	OsG1	LOC_Os07g04670.1	148	1.40E-42	57
	Solanum lycopersicum	SolyLSH1a	Solyc05g055020.4.1	243	2.00E-81	92
	Solanum lycopersicum	TMF	Solyc09g090180.1.1	238	7.00E-80	82
	Solanum lycopersicum	SolyLSH3b	Solyc09g025280.1.1	239	7.00E-80	66
	Solanum lycopersicum	SolyLSH5	Solyc06g082210.1.1	233	5.00E-78	92
	Solanum lycopersicum	SolyLSH1b	Solyc04g009980.4.1	235	5.00E-78	67
	Solanum lycopersicum	SolyLSH3a	Solyc06g083860.2.1	231	2.00E-76	94

QUERY (Access Code)	Target Species	Gene Name	Access Code	Score	E-Value	Identity %
	Solanum lycopersicum	SolyLSH2	Solyc02g069510.1.1	220	2.00E-72	75
	Solanum lycopersicum	SolyLSH7c	Solyc12g014260.1.1	216	2.00E-71	73
	Solanum lycopersicum	SolyLSH10b	Solyc07g150147.1.1	217	1.00E-70	66
	Solanum lycopersicum	SolyLSH7a	Solyc10g007310.1.1	216	1.00E-70	69
	Solanum lycopersicum	SolyLSH10a	Solyc10g008000.1.1	214	7.00E-70	66
	Solanum lycopersicum	SolyLSH10c	Solyc02g076820.4.1	213	1.00E-69	64
	Solanum lycopersicum	SolyLSH7b	Solyc07g062470.4.1	188	4.00E-60	57
LSH4 (AT3G23290.2)	*Chara brawnii*		Mapoly0028s0118.1.p	208	7.85E-67	86
	Marchantia polymorpha	MpLOS1	Mapoly0221s0004.1.p	203	3.53E-64	75
	Marchantia polymorpha	MpLOS2	Pp3c8_6310V3.1.p	208	9.40E-66	85
	Physcomitrella patens		Pp3c20_17990V3.1.p	213	1.80E-67	85
	Physcomitrella patens		Pp3c24_8490V3.1.p	208	1.50E-65	84
	Physcomitrella patens		Pp3c23_9660V3.1.p	209	5.70E-66	84
	Physcomitrella patens		MA_12926g0010	213	1.08E-70	87
	Picea abiens		MA_211369g0010	194	3.23E-63	73
	Picea abiens		MA_139833g0010	136	4.06E-40	88

QUERY (Access Code)	Target Species	Gene Name	Access Code	Score	E-Value	Identity %
	Picea abiens		AT5G28490.1	208	1.15E-68	87
	Arabidopsis thaliana	LSH1	AT3G23290.2	297	6.78E-104	100
	Arabidopsis thaliana	LSH4	AT2G31160.1	229	1.02E-76	93
	Arabidopsis thaliana	LSH3	AT3G04510.1	204	3.94E-67	83
	Arabidopsis thaliana	LSH2	AT5G58500.1	224	5.07E-75	80
	Arabidopsis thaliana	LSH5	AT2G42610.1	213	5.64E-71	78
	Arabidopsis thaliana	LSH10	AT1G07090.1	232	3.81E-78	81
	Arabidopsis thaliana	LSH6	AT1G78815.1	201	4.05E-66	71
	Arabidopsis thaliana	LSH7	AT4G18610.1	196	7.41E-64	71
	Arabidopsis thaliana	LSH9	AT1G16910.1	178	3.47E-57	64
	Arabidopsis thaliana	LSH8	LOC_Os05g28040.1	204	2.70E-64	83
	Oryza sativa	OsG1L9	LOC_Os01g61310.1	213	7.40E-69	84
	Oryza sativa	OsG1L7	LOC_Os02g41460.1	215	2.10E-69	87
	Oryza sativa	OsG1L3	LOC_Os02g56610.1	226	1.80E-73	90
	Oryza sativa	OsG1L6 (TH1/BH1/AFD1)	LOC_Os02g07030.1	218	3.20E-70	87
	Oryza sativa	OsG1L1	LOC_Os06g46030.1	217	1.60E-69	90

**QUERY (Access Code)**	**Target Species**	**Gene Name**	**Access Code**	**Score**	**E-Value**	**Identity %**
	Oryza sativa	OsG1L2	LOC_Os10g33780.1	218	4.80E-71	88
	Oryza sativa	OsG1L5 (TAWAWA1)	LOC_Os04g43580.2	217	2.10E-70	88
	Oryza sativa	OsG1L4	LOC_Os05g39500.1	206	8.80E-66	80
	Oryza sativa	OsG1L8	LOC_Os07g04670.1	157	3.70E-46	59
	Oryza sativa	OsG1	Solyc09g090180.1.1	253	1.00E-85	95
	Solanum lycopersicum	SolyLSH1a	Solyc02g069510.1.1	244	2.00E-82	84
	Solanum lycopersicum	TMF	Solyc06g082210.1.1	238	1.00E-79	74
	Solanum lycopersicum	SolyLSH3b	Solyc05g055020.4.1	236	5.00E-79	69
	Solanum lycopersicum	SolyLSH5	Solyc09g025280.1.1	232	6.00E-78	89
	Solanum lycopersicum	SolyLSH1b	Solyc06g083860.2.1	228	1.00E-75	90
	Solanum lycopersicum	SolyLSH3a	Solyc02g076820.4.1	227	3.00E-75	70
	Solanum lycopersicum	SolyLSH2	Solyc04g009980.4.1	223	2.00E-74	74
	Solanum lycopersicum	SolyLSH7c	Solyc12g014260.1.1	224	4.00E-74	80
	Solanum lycopersicum	SolyLSH10b	Solyc10g007310.1.1	222	2.00E-73	80
	Solanum lycopersicum	SolyLSH7a	Solyc07g150147.1.1	219	1.00E-72	79
	Solanum lycopersicum	SolyLSH10a	Solyc10g008000.1.1	212	5.00E-70	73
	Solanum lycopersicum	SolyLSH10c	Solyc07g062470.4.1	189	8.00E-61	71

QUERY (Access Code)	Target Species	Gene Name	Access Code	Score	E-Value	Identity %
	Solanum lycopersicum	SolyLSH7b	Mapoly0028s0118.1.p	208	7.85E-67	86
LSH5 (AT5G58500.1)	*Chara brawnii*		Mapoly0028s0118.1.p	205	1.48E-65	77
	Marchantia polymorpha	MpLOS1	Mapoly0221s0004.1.p	190	3.15E-59	67
	Marchantia polymorpha	MpLOS2	Pp3c8_6310V3.1.p	208	8.28E-67	78
	Physcomitrella patens		Pp3c20_17990V3.1.p	208	1.05E-66	78
	Physcomitrella patens		Pp3c24_8490V3.1.p	204	2.93E-65	77
	Physcomitrella patens		Pp3c23_9660V3.1.p	206	3.60E-66	78
	Physcomitrella patens		MA_12926g0010	213	4.65E-71	81
	Picea abiens		MA_211369g0010	183	3.97E-59	69
	Picea abiens		MA_139833g0010	136	3.10E-40	59
	Picea abiens		AT5G28490.1	186	1.86E-60	66
	Arabidopsis thaliana	LSH1	AT3G23290.2	202	1.93E-66	80
	Arabidopsis thaliana	LSH4	AT2G31160.1	208	1.52E-68	78
	Arabidopsis thaliana	LSH3	AT3G04510.1	189	2.02E-61	71
	Arabidopsis thaliana	LSH2	AT5G58500.1	309	8.43E-109	100
	Arabidopsis thaliana	LSH5	AT2G42610.1	206	2.75E-68	73

QUERY (Access Code)	Target Species	Gene Name	Access Code	Score	E-Value	Identity %
	Arabidopsis thaliana	LSH10	AT1G07090.1	246	6.78E-84	82
	Arabidopsis thaliana	LSH6	AT1G78815.1	204	2.27E-67	72
	Arabidopsis thaliana	LSH7	AT4G18610.1	206	2.48E-68	73
	Arabidopsis thaliana	LSH9	AT1G16910.1	186	1.86E-60	66
	Arabidopsis thaliana	LSH8	LOC_Os05g28040.1	214	3.34E-69	82
	Oryza sativa	OsG1L9	LOC_Os01g61310.1	220	7.50E-73	83
	Oryza sativa	OsG1L7	LOC_Os02g41460.1	211	4.17E-69	77
	Oryza sativa	OsG1L3	LOC_Os02g56610.1	213	2.42E-69	78
	Oryza sativa	OsG1L6 (TH1/BH1/AFD1)	LOC_Os02g07030.1	210	4.70E-68	79
	Oryza sativa	OsG1L1	LOC_Os06g46030.1	211	4.34E-68	80
	Oryza sativa	OsG1L2	LOC_Os10g33780.1	202	7.42E-66	78
	Oryza sativa	OsG1L5 (TAWAWA1)	LOC_Os04g43580.2	201	2.37E-65	78
	Oryza sativa	OsG1L4	LOC_Os05g39500.1	215	1.97E-70	81
	Oryza sativa	OsG1L8	LOC_Os07g04670.1	161	1.39E-48	56
	Oryza sativa	OsG1	Solyc06g082210.1.1	243	1.00E-81	74
	Solanum lycopersicum	SolyLSH1a	Solyc02g069510.1.1	225	2.00E-75	75

QUERY (Access Code)	Target Species	Gene Name	Access Code	Score	E-Value	Identity %
	Solanum lycopersicum	TMF	Solyc07g150147.1.1	212	5.00E-70	72
	Solanum lycopersicum	SolyLSH3b	Solyc09g090180.1.1	212	1.00E-69	79
	Solanum lycopersicum	SolyLSH5	Solyc10g008000.1.1	207	3.00E-68	59
	Solanum lycopersicum	SolyLSH1b	Solyc10g007310.1.1	208	3.00E-68	70
	Solanum lycopersicum	SolyLSH3a	Solyc12g014260.1.1	207	1.00E-67	76
	Solanum lycopersicum	SolyLSH2	Solyc05g055020.4.1	206	2.00E-67	68
	Solanum lycopersicum	SolyLSH7c	Solyc02g076820.4.1	206	3.00E-67	68
	Solanum lycopersicum	SolyLSH10b	Solyc04g009980.4.1	204	4.00E-67	65
	Solanum lycopersicum	SolyLSH7a	Solyc09g025280.1.1	201	5.00E-66	76
	Solanum lycopersicum	SolyLSH10a	Solyc06g083860.2.1	196	2.00E-63	77
	Solanum lycopersicum	SolyLSH10c	Solyc07g062470.4.1	178	1.00E-56	72
	Solanum lycopersicum	SolyLSH7b	Mapoly0028s0118.1.p	205	1.48E-65	77
LSH6 (AT1G07090.1)	*Chara brawnii*		GBG79305	255	5.40E-27	64
	Marchantia polymorpha	MpLOS1	Mapoly0028s0118.1.p	207	2.58E-66	80
	Marchantia polymorpha	MpLOS2	Mapoly0221s0004.1.p	196	8.62E-62	73
	Physcomitrella patens		Pp3c8_6310V3.1.p	207	1.86E-66	78

QUERY (Access Code)	Target Species	Gene Name	Access Code	Score	E-Value	Identity %
	Physcomitrella patens		Pp3c20_17990V3.1.p	210	1.40E-67	79
	Physcomitrella patens		Pp3c24_8490V3.1.p	209	1.40E-67	79
	Physcomitrella patens		Pp3c23_9660V3.1.p	209	4.90E-67	79
	Picea abiens		MA_12926g0010	216	7.89E-72	83
	Picea abiens		MA_211369g0010	188	1.05E-60	71
	Picea abiens		MA_139833g0010	140	1.03E-41	59
	Arabidopsis thaliana	LSH1	AT5G28490.1	198	5.92E-65	71
	Arabidopsis thaliana	LSH4	AT3G23290.2	215	3.73E-71	81
	Arabidopsis thaliana	LSH3	AT2G31160.1	208	4.11E-68	77
	Arabidopsis thaliana	LSH2	AT3G04510.1	193	1.47E-62	73
	Arabidopsis thaliana	LSH5	AT5G58500.1	233	1.11E-78	84
	Arabidopsis thaliana	LSH10	AT2G42610.1	220	9.23E-74	75
	Arabidopsis thaliana	LSH6	AT1G07090.1	369	2.49E-132	100
	Arabidopsis thaliana	LSH7	AT1G78815.1	204	4.34E-67	69
	Arabidopsis thaliana	LSH9	AT4G18610.1	203	1.59E-66	74
	Arabidopsis thaliana	LSH8	AT1G16910.1	195	1.06E-63	62
	Oryza sativa	OsG1L9	LOC_Os05g28040.1	222	3.21E-72	87

QUERY (Access Code)	Target Species	Gene Name	Access Code	Score	E-Value	Identity %
	Oryza sativa	OsG1L7	LOC_Os01g61310.1	217	4.96E-71	83
	Oryza sativa	OsG1L3	LOC_Os02g41460.1	219	6.04E-72	80
	Oryza sativa	OsG1L6 (TH1/BH1/AFD1)	LOC_Os02g56610.1	213	2.93E-69	81
	Oryza sativa	OsG1L1	LOC_Os02g07030.1	210	1.07E-67	78
	Oryza sativa	OsG1L2	LOC_Os06g46030.1	212	1.91E-68	81
	Oryza sativa	OsG1L5 (TAWAWA1)	LOC_Os10g33780.1	217	3.79E-71	81
	Oryza sativa	OsG1L4	LOC_Os04g43580.2	219	4.76E-72	80
	Oryza sativa	OsG1L8	LOC_Os05g39500.1	215	2.85E-70	82
	Oryza sativa	OsG1	LOC_Os07g04670.1	158	2.44E-47	57
	Solanum lycopersicum	SolyLSH1a	Solyc06g082210.1.1	243	1.00E-81	74
	Solanum lycopersicum	TMF	Solyc02g069510.1.1	225	2.00E-75	75
	Solanum lycopersicum	SolyLSH3b	Solyc07g150147.1.1	212	5.00E-70	72
	Solanum lycopersicum	SolyLSH5	Solyc09g090180.1.1	212	1.00E-69	79
	Solanum lycopersicum	SolyLSH1b	Solyc10g008000.1.1	207	3.00E-68	59
	Solanum lycopersicum	SolyLSH3a	Solyc10g007310.1.1	208	3.00E-68	70
	Solanum lycopersicum	SolyLSH2	Solyc12g014260.1.1	207	1.00E-67	76

QUERY (Access Code)	Target Species	Gene Name	Access Code	Score	E-Value	Identity %
	Solanum lycopersicum	SolyLSH7c	Solyc05g055020.4.1	206	2.00E-67	68
	Solanum lycopersicum	SolyLSH10b	Solyc02g076820.4.1	206	3.00E-67	68
	Solanum lycopersicum	SolyLSH7a	Solyc04g009980.4.1	204	4.00E-67	65
	Solanum lycopersicum	SolyLSH10a	Solyc09g025280.1.1	201	5.00E-66	76
	Solanum lycopersicum	SolyLSH10c	Solyc06g083860.2.1	196	2.00E-63	77
	Solanum lycopersicum	SolyLSH7b	Solyc07g062470.4.1	178	1.00E-56	72
LSH7 (AT1G78815.1	*Chara brawnii*		GBG79305	226	1.80E-23	64
	Marchantia polymorpha	MpLOS1	Mapoly0028s0118.1 .p	180	8.31E-56	69
	Marchantia polymorpha	MpLOS2	Mapoly0221s0004.1 .p	178	2.25E-54	65
	Physcomitrella patens		Pp3c8_6310V3.1.p	180	1.71E-55	68
	Physcomitrella patens		Pp3c20_17990V3.1. p	176	4.17E-54	67
	Physcomitrella patens		Pp3c24_8490V3.1.p	175	6.23E-54	68
	Physcomitrella patens		Pp3c23_9660V3.1.p	175	1.16E-53	67
	Picea abiens		MA_12926g0010	175	9.60E-56	68

**QUERY (Access Code)**	**Target Species**	**Gene Name**	**Access Code**	**Score**	**E-Value**	**Identity %**
	Picea abiens		MA_211369g0010	159	2.61E-49	59
	Picea abiens		MA_139833g0010	119	1.21E-33	57
	Arabidopsis thaliana	LSH1	AT5G28490.1	182	1.42E-58	70
	Arabidopsis thaliana	LSH4	AT3G23290.2	188	1.32E-60	71
	Arabidopsis thaliana	LSH3	AT2G31160.1	179	9.06E-57	69
	Arabidopsis thaliana	LSH2	AT3G04510.1	183	1.36E-58	69
	Arabidopsis thaliana	LSH5	AT5G58500.1	204	2.87E-67	72
	Arabidopsis thaliana	LSH10	AT2G42610.1	216	2.55E-72	77
	Arabidopsis thaliana	LSH6	AT1G07090.1	204	6.25E-67	69
	Arabidopsis thaliana	LSH7	AT1G78815.1	314	2.25E-110	100
	Arabidopsis thaliana	LSH9	AT4G18610.1	201	3.93E-66	74
	Arabidopsis thaliana	LSH8	AT1G16910.1	224	1.95E-75	82
	Oryza sativa	OsG1L9	LOC_Os05g28040.1	189	1.90E-59	68
	Oryza sativa	OsG1L7	LOC_Os01g61310.1	193	5.05E-62	71
	Oryza sativa	OsG1L3	LOC_Os02g41460.1	179	1.99E-56	69

QUERY (Access Code)	Target Species	Gene Name	Access Code	Score	E-Value	Identity %
	Oryza sativa	OsG1L6 (TH1/BH1/AFD1)	LOC_Os02g56610.1	180	2.85E-56	67
	Oryza sativa	OsG1L1	LOC_Os02g07030.1	180	5.82E-56	67
	Oryza sativa	OsG1L2	LOC_Os06g46030.1	180	9.12E-56	70
	Oryza sativa	OsG1L5 (TAWAWA1)	LOC_Os10g33780.1	178	8.19E-56	67
	Oryza sativa	OsG1L4	LOC_Os04g43580.2	180	1.26E-56	69
	Oryza sativa	OsG1L8	LOC_Os05g39500.1	187	3.10E-59	67
	Oryza sativa	OsG1	LOC_Os07g04670.1	131	4.93E-37	46
	Solanum lycopersicum	SolyLSH1a	Solyc10g007310.1.1	232	2.00E-77	74
	Solanum lycopersicum	TMF	Solyc12g014260.1.1	231	3.00E-77	78
	Solanum lycopersicum	SolyLSH3b	Solyc07g150147.1.1	221	4.00E-73	66
	Solanum lycopersicum	SolyLSH5	Solyc10g008000.1.1	217	5.00E-72	59

QUERY (Access Code)	Target Species	Gene Name	Access Code	Score	E-Value	Identity %
	Solanum lycopersicum	SolyLSH1b	Solyc02g076820.4.1	218	1.00E-71	75
	Solanum lycopersicum	SolyLSH3a	Solyc05g055020.4.1	208	5.00E-68	70
	Solanum lycopersicum	SolyLSH2	Solyc07g062470.4.1	205	6.00E-67	63
	Solanum lycopersicum	SolyLSH7c	Solyc04g009980.4.1	201	1.00E-65	65
	Solanum lycopersicum	SolyLSH10b	Solyc06g082210.1.1	201	8.00E-65	67
	Solanum lycopersicum	SolyLSH7a	Solyc02g069510.1.1	196	2.00E-63	64
	Solanum lycopersicum	SolyLSH10a	Solyc09g090180.1.1	195	9.00E-63	69
	Solanum lycopersicum	SolyLSH10c	Solyc09g025280.1.1	177	2.00E-56	64
	Solanum lycopersicum	SolyLSH7b	Solyc06g083860.2.1	177	1.00E-55	67
LSH8 (AT1G16910.1)	*Chara brawnii*		GBG79305	193	5.80E-19	53
	Marchantia polymorpha	MpLOS1	Mapoly0028s0118.1.p	153	1.50E-45	65

QUERY (Access Code)	Target Species	Gene Name	Access Code	Score	E-Value	Identity %
	Marchantia polymorpha	MpLOS2	Mapoly0221s0004.1.p	151	1.06E-44	59
	Physcomitrella patens		Pp3c8_6310V3.1.p	149	2.83E-44	63
	Physcomitrella patens		Pp3c20_17990V3.1.p	148	1.02E-43	63
	Physcomitrella patens		Pp3c24_8490V3.1.p	146	3.92E-43	63
	Physcomitrella patens		Pp3c23_9660V3.1.p	145	1.14E-42	63
	Picea abiens		MA_12926g0010	149	6.58E-46	63
	Picea abiens		MA_211369g0010	130	1.98E-38	54
	Picea abiens		MA_139833g0010	105	8.68E-29	47
	Arabidopsis thaliana	LSH1	AT5G28490.1	162	4.15E-51	66
	Arabidopsis thaliana	LSH4	AT3G23290.2	153	2.60E-47	64
	Arabidopsis thaliana	LSH3	AT2G31160.1	145	3.20E-44	61
	Arabidopsis thaliana	LSH2	AT3G04510.1	157	4.00E-49	66
	Arabidopsis thaliana	LSH5	AT5G58500.1	172	3.15E-55	66
	Arabidopsis thaliana	LSH10	AT2G42610.1	196	1.19E-64	65
	Arabidopsis thaliana	LSH6	AT1G07090.1	187	7.93E-61	63
	Arabidopsis thaliana	LSH7	AT1G78815.1	217	1.14E-72	82

**QUERY (Access Code)**	**Target Species**	**Gene Name**	**Access Code**	**Score**	**E-Value**	**Identity %**
	Arabidopsis thaliana	LSH9	AT4G18610.1	179	9.66E-58	69
	Arabidopsis thaliana	LSH8	AT1G16910.1	300	4.82E-106	100
	Oryza sativa	OsG1L9	LOC_Os05g28040.1	159	5.32E-48	65
	Oryza sativa	OsG1L7	LOC_Os01g61310.1	162	3.43E-50	68
	Oryza sativa	OsG1L3	LOC_Os02g41460.1	153	1.81E-46	62
	Oryza sativa	OsG1L6 (TH1/BH1/AFD1)	LOC_Os02g56610.1	152	8.38E-46	64
	Oryza sativa	OsG1L1	LOC_Os02g07030.1	146	2.08E-43	60
	Oryza sativa	OsG1L2	LOC_Os06g46030.1	151	4.17E-45	63
	Oryza sativa	OsG1L5 (TAWAWA1)	LOC_Os10g33780.1	151	6.93E-46	63
	Oryza sativa	OsG1L4	LOC_Os04g43580.2	153	1.12E-46	61
	Oryza sativa	OsG1L8	LOC_Os05g39500.1	159	1.74E-48	60
	Oryza sativa	OsG1	LOC_Os07g04670.1	115	3.59E-31	43

QUERY (Access Code)	Target Species	Gene Name	Access Code	Score	E-Value	Identity %
	Solanum lycopersicum	SolyLSH1a	Solyc10g007310.1.1	210	4.00E-69	73
	Solanum lycopersicum	TMF	Solyc12g014260.1.1	206	1.00E-67	73
	Solanum lycopersicum	SolyLSH3b	Solyc07g150147.1.1	206	1.00E-67	69
	Solanum lycopersicum	SolyLSH5	Solyc10g008000.1.1	198	5.00E-65	60
	Solanum lycopersicum	SolyLSH1b	Solyc02g076820.4.1	198	2.00E-64	62
	Solanum lycopersicum	SolyLSH3a	Solyc05g055020.4.1	192	5.00E-62	59
	Solanum lycopersicum	SolyLSH2	Solyc07g062470.4.1	189	3.00E-61	64
	Solanum lycopersicum	SolyLSH7c	Solyc04g009980.4.1	182	2.00E-58	62
	Solanum lycopersicum	SolyLSH10b	Solyc02g069510.1.1	181	2.00E-58	62
	Solanum lycopersicum	SolyLSH7a	Solyc06g082210.1.1	178	2.00E-56	65
	Solanum lycopersicum	SolyLSH10a	Solyc09g090180.1.1	174	3.00E-55	66

QUERY (Access Code)	Target Species	Gene Name	Access Code	Score	E-Value	Identity %
	Solanum lycopersicum	SolyLSH10c	Solyc09g025280.1.1	156	1.00E-48	63
LSH9 (AT4G18610.1)	*Chara brawnii*		GBG79305	213	1.50E-21	61
	Marchantia polymorpha	MpLOS1	Mapoly0028s0118.1.p	174	2.76E-53	70
	Marchantia polymorpha	MpLOS2	Mapoly0221s0004.1.p	172	2.82E-52	64
	Physcomitrella patens		Pp3c8_6310V3.1.p	170	6.56E-52	68
	Physcomitrella patens		Pp3c20_17990V3.1.p	175	1.32E-53	68
	Physcomitrella patens		Pp3c24_8490V3.1.p	171	4.40E-52	67
	Physcomitrella patens		Pp3c23_9660V3.1.p	169	1.39E-51	66
	Picea abiens		MA_12926g0010	183	9.19E-59	73
	Picea abiens		MA_211369g0010	160	7.25E-50	61
	Picea abiens		MA_139833g0010	121	2.30E-34	55
	Arabidopsis thaliana	LSH1	AT5G28490.1	175	1.02E-55	69
	Arabidopsis thaliana	LSH4	AT3G23290.2	171	4.18E-54	64
	Arabidopsis thaliana	LSH3	AT2G31160.1	171	6.45E-54	67

QUERY (Access Code)	Target Species	Gene Name	Access Code	Score	E-Value	Identity %
	Arabidopsis thaliana	LSH2	AT3G04510.1	179	3.22E-57	65
	Arabidopsis thaliana	LSH5	AT5G58500.1	215	1.28E-71	69
	Arabidopsis thaliana	LSH10	AT2G42610.1	213	4.44E-71	73
	Arabidopsis thaliana	LSH6	AT1G07090.1	217	3.58E-72	70
	Arabidopsis thaliana	LSH7	AT1G78815.1	202	3.03E-66	74
	Arabidopsis thaliana	LSH9	AT4G18610.1	331	1.93E-117	100
	Arabidopsis thaliana	LSH8	AT1G16910.1	193	2.52E-63	69
	Oryza sativa	OsG1L9	LOC_Os05g28040.1	181	2.92E-56	69
	Oryza sativa	OsG1L7	LOC_Os01g61310.1	188	9.40E-60	70
	Oryza sativa	OsG1L3	LOC_Os02g41460.1	176	4.37E-55	69
	Oryza sativa	OsG1L6 (TH1/BH1/AFD1)	LOC_Os02g56610.1	175	3.41E-54	68
	Oryza sativa	OsG1L1	LOC_Os02g07030.1	170	3.53E-52	65
	Oryza sativa	OsG1L2	LOC_Os06g46030.1	173	5.31E-53	68

QUERY (Access Code)	Target Species	Gene Name	Access Code	Score	E-Value	Identity %
	Oryza sativa	OsG1L5 (TAWAWA1)	LOC_Os10g33780.1	166	1.58E-51	69
	Oryza sativa	OsG1L4	LOC_Os04g43580.2	167	8.75E-52	69
	Oryza sativa	OsG1L8	LOC_Os05g39500.1	183	1.65E-57	69
	Oryza sativa	OsG1	LOC_Os07g04670.1	142	3.21E-41	50
	Solanum lycopersicum	SolyLSH1a	Solyc10g007310.1.1	221	5.00E-73	71
	Solanum lycopersicum	TMF	Solyc12g014260.1.1	220	1.00E-72	75
	Solanum lycopersicum	SolyLSH3b	Solyc02g076820.4.1	219	5.00E-72	77
	Solanum lycopersicum	SolyLSH5	Solyc07g150147.1.1	215	5.00E-71	79
	Solanum lycopersicum	SolyLSH1b	Solyc10g008000.1.1	209	6.00E-69	71
	Solanum lycopersicum	SolyLSH3a	Solyc05g055020.4.1	196	2.00E-63	61
	Solanum lycopersicum	SolyLSH2	Solyc02g069510.1.1	194	5.00E-63	64

QUERY (Access Code)	Target Species	Gene Name	Access Code	Score	E-Value	Identity %
	Solanum lycopersicum	SolyLSH7c	Solyc04g009980.4.1	192	3.00E-62	62
	Solanum lycopersicum	SolyLSH10b	Solyc07g062470.4.1	191	2.00E-61	69
	Solanum lycopersicum	SolyLSH7a	Solyc06g082210.1.1	189	4.00E-60	65
	Solanum lycopersicum	SolyLSH10a	Solyc09g090180.1.1	182	7.00E-58	72
	Solanum lycopersicum	SolyLSH10c	Solyc09g025280.1.1	169	2.00E-53	67
	Solanum lycopersicum	SolyLSH7b	Solyc06g083860.2.1	163	2.00E-50	68
LSH10 (AT2G42610.1)	*Chara brawnii*		GBG79305	268	4.40E-29	69
	Marchantia polymorpha	MpLOS1	Mapoly0028s0118.1.p	200	8.64E-64	77
	Marchantia polymorpha	MpLOS2	Mapoly0221s0004.1.p	195	1.93E-61	72
	Physcomitrella patens		Pp3c8_6310V3.1.p	193	3.03E-61	75
	Physcomitrella patens		Pp3c20_17990V3.1.p	191	2.14E-60	65

QUERY (Access Code)	Target Species	Gene Name	Access Code	Score	E-Value	Identity %
	Physcomitrella patens		Pp3c24_8490V3.1.p	192	9.78E-61	71
	Physcomitrella patens		Pp3c23_9660V3.1.p	191	3.75E-60	74
	Picea abiens		MA_12926g0010	200	5.81E-66	77
	Picea abiens		MA_211369g0010	174	1.74E-55	66
	Picea abiens		MA_139833g0010	126	1.06E-36	81
	Arabidopsis thaliana	LSH1	AT5G28490.1	189	1.26E-61	72
	Arabidopsis thaliana	LSH4	AT3G23290.2	193.36	4.03E-63	78
	Arabidopsis thaliana	LSH3	AT2G31160.1	196	7.36E-64	75
	Arabidopsis thaliana	LSH2	AT3G04510.1	186	4.77E-60	73
	Arabidopsis thaliana	LSH5	AT5G58500.1	208	2.57E-69	69
	Arabidopsis thaliana	LSH10	AT2G42610.1	328	1.56E-116	100
	Arabidopsis thaliana	LSH6	AT1G07090.1	221	4.19E-74	74
	Arabidopsis thaliana	LSH7	AT1G78815.1	218	8.11E-73	77
	Arabidopsis thaliana	LSH9	AT4G18610.1	213	8.94E-71	79
	Arabidopsis thaliana	LSH8	AT1G16910.1	204	1.01E-67	65
	Oryza sativa	OsG1L9	LOC_Os05g28040.1	192	7.11E-61	76

QUERY (Access Code)	Target Species	Gene Name	Access Code	Score	E-Value	Identity %
	Oryza sativa	OsG1L7	LOC_Os01g61310.1	200	8.10E-65	78
	Oryza sativa	OsG1L3	LOC_Os02g41460.1	196	2.92E-63	75
	Oryza sativa	OsG1L6 (TH1/BH1/AFD1)	LOC_Os02g56610.1	196	6.18E-63	75
	Oryza sativa	OsG1L1	LOC_Os02g07030.1	190	5.17E-60	71
	Oryza sativa	OsG1L2	LOC_Os06g46030.1	190	6.27E-60	76
	Oryza sativa	OsG1L5 (TAWAWA1)	LOC_Os10g33780.1	195	3.31E-63	77
	Oryza sativa	OsG1L4	LOC_Os04g43580.2	195	4.92E-63	73
	Oryza sativa	OsG1L8	LOC_Os05g39500.1	196	4.97E-63	75
	Oryza sativa	OsG1	LOC_Os07g04670.1	142	2.94E-41	53
	Solanum lycopersicum	SolyLSH1a	Solyc07g150147.1.1	261	3.00E-89	85
	Solanum lycopersicum	TMF	Solyc10g008000.1.1	256	2.00E-87	74

QUERY (Access Code)	Target Species	Gene Name	Access Code	Score	E-Value	Identity %
	Solanum lycopersicum	SolyLSH3b	Solyc02g076820.4.1	248	2.00E-83	79
	Solanum lycopersicum	SolyLSH5	Solyc10g007310.1.1	243	4.00E-82	74
	Solanum lycopersicum	SolyLSH1b	Solyc12g014260.1.1	238	4.00E-80	86
	Solanum lycopersicum	SolyLSH3a	Solyc09g090180.1.1	211	2.00E-69	76
	Solanum lycopersicum	SolyLSH2	Solyc06g082210.1.1	211	3.00E-69	75
	Solanum lycopersicum	SolyLSH7c	Solyc07g062470.4.1	210	4.00E-69	80
	Solanum lycopersicum	SolyLSH10b	Solyc02g069510.1.1	207	2.00E-68	70
	Solanum lycopersicum	SolyLSH7a	Solyc05g055020.4.1	204	1.00E-66	65
	Solanum lycopersicum	SolyLSH10a	Solyc09g025280.1.1	198	9.00E-65	76
	Solanum lycopersicum	SolyLSH10c	Solyc04g009980.4.1	196	4.00E-64	67
	Solanum lycopersicum	SolyLSH7b	Solyc06g083860.2.1	194	1.00E-62	76
