# Supplementary material for: Genome-wide identification and evolutionary view of ALOG gene family in Solanaceae
Source: Genet Mol Biol. 2023 Dec 1;46(3 Suppl 1):e20230142. doi: 10.1590/1415-4757-GMB-2023-0142 (PMC10695626; doi:10.1590/1415-4757-GMB-2023-0142)
Supplement: Table S3 - [file 1415-4757-GMB-46-3-s1-e20230142-s3.pdf]

Genetics and Molecular Biology

https://doi.org/10.1590/1678-4685-GMB-2023-0142

Supplementary Material to "Genome-wide identification and evolutionary view of ALOG gene family in Solanaceae"

Table S3 – Information regarding BLAST statistics for each target species selected for this study.

Database	Acr	Species	Subject ID	Gene name	Id%	E-value	Score	Description
FernBase	Adcap	*Adiantum capillus*	**ADC24086**		72.61	1,00E-28	126	Length=963
FernBase	Adcap	*Adiantum capillus*	**ADC30290**		71.72	1,00E-23	110	Length=873
FernBase	Adcap	*Adiantum capillus*	**ADC29831**		74.86	4,00E-14	78.7	Length=732
FernBase	Alspi	*Alsophila spinulosa*	**Aspi01Gene12712.t1**		72.68	7,00E-30	132	Length=753
FernBase	Alspi	*Alsophila spinulosa*	**Aspi01Gene29667.t1**		72.06	2,00E-25	117	Length=786
FernBase	Alspi	*Alsophila spinulosa*	**Aspi01Gene46302.t1**		71.88	9,00E-24	111	Length=747
FernBase	Alspi	*Alsophila spinulosa*	**Aspi01Gene32958.t1**		71.18	9,00E-19	95.3	Length=783
FernBase	Alspi	*Alsophila spinulosa*	**Aspi01Gene66763.t1**		70.79	3,00E-18	93.5	Length=783
FernBase	Alspi	*Alsophila spinulosa*	**Aspi01Gene25077.t1**		76.28	7,00E-15	82.4	Length=780
Phytozome	Amtri	*Amborella trichopoda*	**evm_27.model.AmTr_v1.0_scaffold00067.31**		92.6	1.6E-72	221.1	(1 of 4) PF04852 - Protein of unknown function (DUF640) (DUF640)

Database	Acr	Species	Subject ID	Gene name	Id%	E-value	Score	Description
Phytozome	Amtri	*Amborella trichopoda*	**evm_27.model.AmTr_v1.0_scaffold00155.8**		81.7	6.5E-67	207.2	(1 of 4) PF04852 - Protein of unknown function (DUF640) (DUF640)
Phytozome	Amtri	*Amborella trichopoda*	**evm_27.model.AmTr_v1.0_scaffold00153.20**		76.3	3.7E-64	199.5	(1 of 4) PF04852 - Protein of unknown function (DUF640) (DUF640)
Phytozome	Amtri	*Amborella trichopoda*	**evm_27.model.AmTr_v1.0_scaffold00153.22**		59.7	1.2E-48	163.7	(1 of 1) PTHR31165:SF6 - PROTEIN LIGHT-DEPENDENT SHORT HYPOCOTYLS 6
Phytozome	Ancom	*Ananas comosus*	**Aco010109.1**		92.3	6.7E-67	208.8	Protein LIGHT-DEPENDENT SHORT HYPOCOTYLS
Phytozome	Ancom	*Ananas comosus*	**Aco031856.1**		200.7	6.8E-63	200.7	Protein LIGHT-DEPENDENT SHORT HYPOCOTYLS
Phytozome	Ancom	*Ananas comosus*	**Aco007739.1**		82.4	1.1E-62	197.6	Protein LIGHT-DEPENDENT SHORT HYPOCOTYLS
Phytozome	Ancom	*Ananas comosus*	**Aco008960.1**		78.9	3.5E-60	191.0	Protein LIGHT-DEPENDENT SHORT HYPOCOTYLS
Phytozome	Ancom	*Ananas comosus*	**Aco008677.1**		65	2.6E-53	172.9	Protein LIGHT-DEPENDENT SHORT HYPOCOTYLS
Phytozome	Ancom	*Ananas comosus*	Aco011089.1		78.6	3.5E-63	206.5	Protein LIGHT-DEPENDENT SHORT HYPOCOTYLS
Phytozome	Ancom	*Ananas comosus*	Aco008403.1		55.9	2.2E-58	187.6	Protein LIGHT-DEPENDENT SHORT HYPOCOTYLS
https://www.hornworts.uzh.ch/en/Blast.html	Anpun	*Anthoceros punctatus*	**Apun_evm.model.utg000090l.263.1**		75	1.E-69	220	Apun_evm.model.utg000090l.263.1 . Apun_evm.TU.utg000090l.263 utg000090l:1219214-1220764(-) Length=517

Database	Acr	Species	Subject ID	Gene name	Id%	E-value	Score	Description
Phytozome	Aqcoe	*Aquilegia coerulea*	**Aqcoe2G087000.1.p**		81	5.34E-78	234.572	(1 of 2) PTHR31165:SF6 - PROTEIN LIGHT-DEPENDENT SHORT HYPOCOTYLS 6
Phytozome	Aqcoe	*Aquilegia coerulea*	**Aqcoe1G225500.1.p**		73	8.49E-73	218.009	(1 of 6) PF04852 - Protein of unknown function (DUF640) (DUF640)
Phytozome	Aqcoe	*Aquilegia coerulea*	**Aqcoe2G375100.1.p**		75	5.07E-71	214.157	(1 of 1) PTHR31165:SF13 - PROTEIN LIGHT-DEPENDENT SHORT HYPOCOTYLS 1-RELATED
Phytozome	Aqcoe	*Aquilegia coerulea*	**Aqcoe5G024600.1.p**		80	1.36E-69	210.305	(1 of 6) PF04852 - Protein of unknown function (DUF640) (DUF640)
Phytozome	Aqcoe	*Aquilegia coerulea*	**Aqcoe4G043800.1.p**		70	3.64E-67	203.371	(1 of 1) PTHR31165:SF10 - PROTEIN LIGHT-DEPENDENT SHORT HYPOCOTYLS 10-RELATED
Phytozome	Aqcoe	*Aquilegia coerulea*	Aqcoe2G265400.1.p		61	6.50E-55	187.963	(1 of 2) PTHR31165:SF6 - PROTEIN LIGHT-DEPENDENT SHORT HYPOCOTYLS 6
Phytozome	Artha	*Arabidopsis thaliana*	**AT2G31160.1**	LSH3	85.4	9.4E-70	214.9	Protein of unknown function (DUF640)
Phytozome	Artha	*Arabidopsis thaliana*	**AT2G42610.1**	LSH10	72.1	5.2E-60	188.7	Protein of unknown function (DUF640)
Phytozome	Artha	*Arabidopsis thaliana*	**AT4G18610.1**	LSH9	69.8	2.6E-52	169.5	Protein of unknown function (DUF640)
Phytozome	Artha	*Arabidopsis thaliana*	**AT1G78815.1**	LSH7	70.4	1.2E-57	183.3	Protein of unknown function (DUF640)
Phytozome	Artha	*Arabidopsis thaliana*	**AT1G07090.1**	LSH6	69.7	7.1E-66	204.5	Protein of unknown function (DUF640)

Database	Acr	Species	Subject ID	Gene name	Id%	E-value	Score	Description
Phytozome	Artha	*Arabidopsis thaliana*	**AT1G16910.1**	LSH8	65.4	2.2E-55	176.4	Protein of unknown function (DUF640)
Phytozome	Artha	*Arabidopsis thaliana*	**AT3G23290.2**	LSH4	87.3	3.2E-68	210.3	Protein of unknown function (DUF640)
Phytozome	Artha	*Arabidopsis thaliana*	**AT3G04510.1**	LSH2	83.5	9.7E-84	250.4	Protein of unknown function (DUF640)
Phytozome	Artha	*Arabidopsis thaliana*	**AT5G28490.1**	LSH1	100	2.4E-111	320.1	Protein of unknown function (DUF640)
Phytozome	Artha	*Arabidopsis thaliana*	**AT5G58500.1**	LSH5	73.5	2.4E-61	192.2	Protein of unknown function (DUF640)
Phytozome	Artha	*Arabidopsis thaliana*	AT4G19500.1	-	33.3	2.2E-12	65.9	nucleoside-triphosphatases;transmembrane receptors;nucleotide binding;ATP binding
FernBase	Azfil	*Azolla filiculoides*	**Azfi_s0048.g030438**		79.08	2,00E-22	106	Protein LIGHT-DEPENDENT SHORT HYPOCOTYLS 6 [0.610] Length=1113
FernBase	Azfil	*Azolla filiculoides*	**Azfi_s0093.g043261**		78.21	1,00E-19	97.1	Protein LIGHT-DEPENDENT SHORT HYPOCOTYLS 6 [0.610] Length=1575
FernBase	Azfil	*Azolla filiculoides*	Azfi_s0639.g080525		73.57	1,00E-24	113	Protein LIGHT-DEPENDENT SHORT HYPOCOTYLS 6 [0.610] Length=1359
FernBase	Azfil	*Azolla filiculoides*	Azfi_s0030.g024231		78.48	3,00E-21	102	Protein LIGHT-DEPENDENT SHORT HYPOCOTYLS 6Length=1308
FernBase	Azfil	*Azolla filiculoides*	Azfi_s0048.g030439		77.78	7,00E-18	91.6	Length=663
Phytozome	Bevul	*Beta vulgaris*	**EL10Ac7g15894.1**		92	2.42e-86	254.988	(1 of 1) PTHR31165//PTHR31165:SF6 - FAMILY NOT NAMED // PROTEIN LIGHT-DEPENDENT

Database	Acr	Species	Subject ID	Gene name	Id%	E-value	Score	Description
								SHORT HYPOCOTYLS 6
Phytozome	Bevul	*Beta vulgaris*	**EL10Ac3g04996.1**		82	9.12e-70	211.075	(1 of 3) PTHR31165:SF13 - PROTEIN LIGHT-DEPENDENT SHORT HYPOCOTYLS 1-RELATED
Phytozome	Bevul	*Beta vulgaris*	**EL10As5g23486.1**		79	1.16e-69	209.92	(1 of 3) PTHR31165//PTHR31165:SF13 - FAMILY NOT NAMED // PROTEIN LIGHT-DEPENDENT SHORT HYPOCOTYLS 1-RELATED
Phytozome	Bevul	*Beta vulgaris*	**EL10Ac4g08340.1**		72	6.08e-69	209.534	(1 of 1) PTHR31165:SF9 - PROTEIN LIGHT-DEPENDENT SHORT HYPOCOTYLS 7-RELATED
Phytozome	Bevul	*Beta vulgaris*	**EL10Ac8g20596.1**		70	1.88e-68	207.223	(1 of 8) PF04852 - Protein of unknown function (DUF640) (DUF640)
Phytozome	Bevul	*Beta vulgaris*	**EL10As5g23487.1**		79	1.19e-67	206.453	(1 of 3) PTHR31165:SF13 - PROTEIN LIGHT-DEPENDENT SHORT HYPOCOTYLS 1-RELATED
Phytozome	Bevul	*Beta vulgaris*	**EL10Ac4g09001.1**		74	1.64e-66	202.986	(1 of 8) PF04852 - Protein of unknown function (DUF640) (DUF640)
Phytozome	Bevul	*Beta vulgaris*	**EL10Ac9g20943.1**		71	6.39e-63	193.356	(1 of 8) PF04852 - Protein of unknown function (DUF640) (DUF640)
Phytozome	Brdis	*Brachypodium distachyon*	**Bradi3g28800.1**		85.7	1.5E-69	214.5	(1 of 10) PF04852 - Protein of unknown function (DUF640)

Database	Acr	Species	Subject ID	Gene name	Id%	E-value	Score	Description
Phytozome	Brdis	*Brachypodium distachyon*	**Bradi2g53880.3**		78.5	1.8E-68	212.2	(1 of 10) PF04852 - Protein of unknown function (DUF640)
Phytozome	Brdis	*Brachypodium distachyon*	**Bradi3g49140.3**		80.3	8.5E-68	209.9	(1 of 10) PF04852 - Protein of unknown function (DUF640)
Phytozome	Brdis	*Brachypodium distachyon*	**Bradi1g32445.1**		78.6	2.3E-67	211.5	(1 of 10) PF04852 - Protein of unknown function (DUF640)
Phytozome	Brdis	*Brachypodium distachyon*	**Bradi5g15700.1**		84	6.4E-67	208.0	(1 of 10) PF04852 - Protein of unknown function (DUF640)
Phytozome	Brdis	*Brachypodium distachyon*	**Bradi3g54800.1**		82.3	1.2E-65	206.1	(1 of 10) PF04852 - Protein of unknown function (DUF640)
Phytozome	Brdis	*Brachypodium distachyon*	**Bradi3g04960.1**		77.9	6.7E-65	204.9	(1 of 10) PF04852 - Protein of unknown function (DUF640)
Phytozome	Brdis	*Brachypodium distachyon*	**Bradi2g29110.2**		76.5	1.8E-64	206.5	(1 of 10) PF04852 - Protein of unknown function (DUF640)
Phytozome	Brdis	*Brachypodium distachyon*	**Bradi2g22561.2**		81	1.4E-62	198.7	(1 of 10) PF04852 - Protein of unknown function (DUF640)
Phytozome	Brdis	*Brachypodium distachyon*	**Bradi1g58070.1**	BdG1	56.6	9.1E-45	152.5	(1 of 10) PF04852 - Protein of unknown function (DUF640)
Phytozome	Carub	*Capsella rubella*	**Carub_Carubv10010340/Carub.0001s0638.1.p**		95	2.44e-120	338.961	
Phytozome	Carub	*Capsella rubella*	**Carub_Carubv10024080/Carub.0004s1126.1.p**		79	2.92e-80	237.654	
Phytozome	Carub	*Capsella rubella*	**Carub_Carubv10027208/Carub.0008s1909.1.p**		82	2.09e-76	227.254	
Phytozome	Carub	*Capsella rubella*	**Carub_Carubv10024159/Carub.0004s2506.1.p**		75	2.22e-74	221.861	
Phytozome	Carub	*Capsella rubella*	**Carub_Carubv10014690/Carub.0003s2352.1.p**		81	1.16e-70	213.001	

Database	Acr	Species	Subject ID	Gene name	Id%	E-value	Score	Description
Phytozome	Carub	*Capsella rubella*	**Carub_Carubv10024036/Carub.0004s1244.1.p**		77	7.17e-68	206.838	
Phytozome	Carub	*Capsella rubella*	**Carub_Carubv10007704/Carub.0007s2261.1.p**		74	6.93e-67	203.371	
Phytozome	Carub	*Capsella rubella*	**Carub_Carubv10020990/Carub.0002s2564.1.p**		69	1.93e-66	202.216	
Phytozome	Carub	*Capsella rubella*	**Carub_Carubv10002037/Carub.0006s2573.1.p**		72	4.79e-66	201.06	
Phytozome	Carub	*Capsella rubella*	**Carub_Carubv10028368/Carub.0008s0208.1.p**		71	2.27e-63	193.741	
Phytozome	Carub	*Capsella rubella*	**Carub_Carubv10014673/Carub.0003s0367.1.p**		73	2.81e-62	191.815	
SOL Genomics	Caann	*Capsicum annuum*	**CA05g19210**		79.17	3,00E-75	224	Detected protein of unknown functionLength=193
SOL Genomics	Caann	*Capsicum annuum*	**CA03g05190**		78.48	1,00E-75	226	Detected protein of unknown functionLength=209
SOL Genomics	Caann	*Capsicum annuum*	**CA06g27930**		83.08	4,00E-70	211	Detected protein of unknown functionLength=194
SOL Genomics	Caann	*Capsicum annuum*	**CA03g10110**		86.29	4,00E-68	206	At2g31160/T16B12.3Length=195
SOL Genomics	Caann	*Capsicum annuum*	**CA02g14140**		74.24	5,00E-70	210	Unknown proteinLength=190
SOL Genomics	Caann	*Capsicum annuum*	**CA10g01660**		69.93	1,00E-69	210	Detected protein of unknown functionLength=199
SOL Genomics	Caann	*Capsicum annuum*	**CA07g17030**		68.42	3,00E-64	196	Detected protein of unknown functionLength=182
SOL Genomics	Caann	*Capsicum annuum*	CA02g00760		72.86	2,00E-65	200	Detected protein of unknown functionLength=215
SOL Genomics	Caann	*Capsicum annuum*	CA00g75150		65.85	3,00E-74	223	Detected protein of unknown functionLength=235

Database	Acr	Species	Subject ID	Gene name	Id%	E-value	Score	Description
SOL Genomics	Caann	*Capsicum annuum*	CA06g25620		73.94	2,00E-69	210	Detected protein of unknown functionLength=237
SOL Genomics	Caann	*Capsicum annuum*	CA00g81480		74.36	5,00E-38	127	Unknown proteinLength=130
NCBI	Cabac	*Capsicum baccatum*	**PHT54399.1**		74.48%	7,00E-80	238	Protein LIGHT-DEPENDENT SHORT HYPOCOTYLS 2 [Capsicum baccatum]
NCBI	Cabac	*Capsicum baccatum*	**PHT39047.1**		78.48%	2,00E-74	226	Protein LIGHT-DEPENDENT SHORT HYPOCOTYLS 6 [Capsicum baccatum]
NCBI	Cabac	*Capsicum baccatum*	**PHT48849.1**		79.17%	4,00E-74	224	Protein LIGHT-DEPENDENT SHORT HYPOCOTYLS 5 [Capsicum baccatum]
NCBI	Cabac	*Capsicum baccatum*	**PHT43573.1**		73.72%	6,00E-70	213	Protein LIGHT-DEPENDENT SHORT HYPOCOTYLS 10 [Capsicum baccatum]
NCBI	Cabac	*Capsicum baccatum*	**PHT35689.1**		71.97%	2,00E-69	211	Protein LIGHT-DEPENDENT SHORT HYPOCOTYLS 6 [Capsicum baccatum]
NCBI	Cabac	*Capsicum baccatum*	**PHT46562.1**		83.85%	4,00E-69	212	Protein G1-like6 [Capsicum baccatum]
NCBI	Cabac	*Capsicum baccatum*	**PHT46699.1**		73.24%	4,00E-69	210	Protein G1-like7 [Capsicum baccatum]
NCBI	Cabac	*Capsicum baccatum*	**PHT30418.1**		73.48%	5,00E-69	208	Protein LIGHT-DEPENDENT SHORT HYPOCOTYLS 6 [Capsicum baccatum]
NCBI	Cabac	*Capsicum baccatum*	**PHT35506.1**		68.97%	4,00E-68	206	Protein LIGHT-DEPENDENT SHORT HYPOCOTYLS 10 [Capsicum baccatum]

**Database**	**Acr**	**Species**	**Subject ID**	**Gene name**	**Id%**	**E-value**	**Score**	**Description**
NCBI	Cabac	*Capsicum baccatum*	**PHT52854.1**		86.29%	5,00E-68	206	Protein G1-like6 [Capsicum baccatum]
NCBI	Cabac	*Capsicum baccatum*	**PHT55504.1**		73.19%	2,00E-65	198	Protein LIGHT-DEPENDENT SHORT HYPOCOTYLS 3 [Capsicum baccatum]
NCBI	Cabac	*Capsicum baccatum*	**PHT44224.1**		68.42%	4,00E-64	195	Protein LIGHT-DEPENDENT SHORT HYPOCOTYLS 6 [Capsicum baccatum]
NCBI	Cachi	*Capsicum chinense*	**PHU17022.1**		74.48%	8,00E-80	239	Protein LIGHT-DEPENDENT SHORT HYPOCOTYLS 6 [Capsicum chinense]
NCBI	Cachi	*Capsicum chinense*	**PHU21623.1**		77.85%	8,00E-74	224	Protein LIGHT-DEPENDENT SHORT HYPOCOTYLS 6 [Capsicum chinense]
NCBI	Cachi	*Capsicum chinense*	**PHU18587.1**		79.17%	8,00E-74	223	Protein LIGHT-DEPENDENT SHORT HYPOCOTYLS 5 [Capsicum chinense]
NCBI	Cachi	*Capsicum chinense*	**PHU04037.1**		72.73%	2,00E-71	213	Protein LIGHT-DEPENDENT SHORT HYPOCOTYLS 6 [Capsicum chinense]
NCBI	Cachi	*Capsicum chinense*	**PHU12480.1**		73.72%	4,00E-71	213	Protein LIGHT-DEPENDENT SHORT HYPOCOTYLS 10 [Capsicum chinense]
NCBI	Cachi	*Capsicum chinense*	**PHU16402.1**		83.08%	4,00E-69	211	Protein LIGHT-DEPENDENT SHORT HYPOCOTYLS 3 [Capsicum chinense]
NCBI	Cachi	*Capsicum chinense*	**PHU26161.1**		74.24%	7,00E-69	211	Protein LIGHT-DEPENDENT SHORT HYPOCOTYLS 6 [Capsicum chinense]

Database	Acr	Species	Subject ID	Gene name	Id%	E-value	Score	Description
NCBI	Cachi	*Capsicum chinense*	**PHU04166.1**		69.93%	8,00E-69	211	Protein LIGHT-DEPENDENT SHORT HYPOCOTYLS 10 [Capsicum chinense]
NCBI	Cachi	*Capsicum chinense*	**PHU16228.1**		73.24%	3,00E-69	210	Protein G1-like7 [Capsicum chinense]
NCBI	Cachi	*Capsicum chinense*	**PHU22638.1**		86.29%	4,00E-68	206	Protein G1-like6 [Capsicum chinense]
NCBI	Cachi	*Capsicum chinense*	**PHU25749.1**		73.72%	4,00E-65	197	Protein LIGHT-DEPENDENT SHORT HYPOCOTYLS 6 [Capsicum chinense]
NCBI	Cachi	*Capsicum chinense*	**PHU12699.1**		67.21%	5,00E-57	176	Protein LIGHT-DEPENDENT SHORT HYPOCOTYLS 6 [Capsicum chinense]
Phytozome	Cepur	*Ceratodon purpureus*	**CepurGG1.12G057300.1.p**		79	5.56E-68	209.92	-
Phytozome	Cepur	*Ceratodon purpureus*	**CepurGG1.12G057600.1.p**		78	7.66E-67	206.838	-
Phytozome	Ceric	*Ceratopteris richardii*	**Ceric.14G090800.1.p**		77	7.43e-82	246.128	(1 of 5) PF04852 - Protein of unknown function (DUF640) (DUF640)
Phytozome	Ceric	*Ceratopteris richardii*	**Ceric.29G008000.1.p**		81	3.99e-74	224.942	(1 of 5) PF04852 - Protein of unknown function (DUF640) (DUF640)
Phytozome	Ceric	*Ceratopteris richardii*	**Ceric.22G071900.1.p**		67	1.77e-71	220.705	(1 of 5) PF04852 - Protein of unknown function (DUF640) (DUF640)
Phytozome	Ceric	*Ceratopteris richardii*	**Ceric.02G086500.1.p**		70	3.39e-71	221.09	(1 of 5) PF04852 - Protein of unknown function (DUF640) (DUF640)
Phytozome	Ceric	*Ceratopteris richardii*	**Ceric.18G075200.1.p**		69	5.43e-60	191.43	(1 of 5) PF04852 - Protein of unknown function (DUF640) (DUF640)
Esembls Plants	Chbra	*Chara braunii*	**GBG79305**		66.1	1.9e-49	228	pep supercontig:Cbr_1.0:BFE

Database	Acr	Species	Subject ID	Gene name	Id%	E-value	Score	Description
								A01000316:20236:21053:1 gene:CBR_g29454 transcript:GBG79305 gene_biotype:protein_coding transcript_biotype:protein_coding description:hypothetical protein
Esembls Plants	Chbra	*Chara braunii*	GBG87458		58.3	0.072	73	pep supercontig:Cbr_1.0:BFEA01000614:436615:440529:1 gene:CBR_g45516 transcript:GBG87458 gene_biotype:protein_coding transcript_biotype:protein_coding description:hypothetical protein
Phytozome	Cikan	*Cinnamomum kanehirae*	**CKAN_00999800**		77	1.97e-85	252.292	(1 of 3) PTHR31165//PTHR31165:SF6 - FAMILY NOT NAMED // PROTEIN LIGHT-DEPENDENT SHORT HYPOCOTYLS 6
Phytozome	Cikan	*Cinnamomum kanehirae*	**CKAN_02768800**		77	2.03e-85	252.292	(1 of 3) PTHR31165//PTHR31165:SF6 - FAMILY NOT NAMED // PROTEIN LIGHT-DEPENDENT SHORT HYPOCOTYLS 6
Phytozome	Cikan	*Cinnamomum kanehirae*	**CKAN_00746700**		85	7.92e-77	229.565	(1 of 3) PTHR31165//PTHR31165:SF6 - FAMILY NOT NAMED // PROTEIN

Database	Acr	Species	Subject ID	Gene name	Id%	E-value	Score	Description
								LIGHT-DEPENDENT SHORT HYPOCOTYLS 6
Phytozome	Cikan	*Cinnamomum kanehirae*	**CKAN_01538100**		68	3.49e-72	216.083	(1 of 1) PTHR31165//PTHR31165:SF9 - FAMILY NOT NAMED // PROTEIN LIGHT-DEPENDENT SHORT HYPOCOTYLS 7-RELATED
Phytozome	Cikan	*Cinnamomum kanehirae*	**CKAN_01828500**		76	1.63e-71	214.542	(1 of 2) PTHR31165//PTHR31165:SF10 - FAMILY NOT NAMED // PROTEIN LIGHT-DEPENDENT SHORT HYPOCOTYLS 10-RELATED
Phytozome	Cikan	*Cinnamomum kanehirae*	**CKAN_01465100**		83	3.04e-71	215.312	(1 of 10) PF04852 - Protein of unknown function (DUF640) (DUF640)
Phytozome	Cikan	*Cinnamomum kanehirae*	**CKAN_02571500**		65	3.17e-71	213.772	(1 of 2) PTHR31165//PTHR31165:SF10 - FAMILY NOT NAMED // PROTEIN LIGHT-DEPENDENT SHORT HYPOCOTYLS 10-RELATED
Phytozome	Cikan	*Cinnamomum kanehirae*	**CKAN_02689100**		72	7.95e-71	212.616	(1 of 10) PF04852 - Protein of unknown function (DUF640) (DUF640)
Phytozome	Cikan	*Cinnamomum kanehirae*	**CKAN_02603200**		78	1.48e-70	212.231	(1 of 10) PF04852 - Protein of unknown function (DUF640) (DUF640)
Phytozome	Cikan	*Cinnamomum kanehirae*	**CKAN_01276000**		80	5.28e-69	208.764	(1 of 1) PTHR31165//PTHR3116

**Database**	**Acr**	**Species**	**Subject ID**	**Gene name**	**Id%**	**E-value**	**Score**	**Description**
								5:SF13 - FAMILY NOT NAMED // PROTEIN LIGHT-DEPENDENT SHORT HYPOCOTYLS 1-RELATED
Phytozome	Cicle	*Citrus clementina*	**Ciclev10005901m**		87	1.7E-70	216.5	(1 of 2) PTHR31165:SF13 - PROTEIN LIGHT-DEPENDENT SHORT HYPOCOTYLS 1-RELATED
Phytozome	Cicle	*Citrus clementina*	**Ciclev10022288m**		76.8	6,00E-76	230.3	(1 of 2) PTHR31165:SF13 - PROTEIN LIGHT-DEPENDENT SHORT HYPOCOTYLS 1-RELATED
Phytozome	Cicle	*Citrus clementina*	**Ciclev10022456m**		73.9	1.3E-61	193.0	(1 of 1) PTHR31165:SF9 - PROTEIN LIGHT-DEPENDENT SHORT HYPOCOTYLS 7-RELATED
Phytozome	Cicle	*Citrus clementina*	**Ciclev10023710m**		74.6	4.9E-60	193.7	(1 of 2) PTHR31165:SF10 - PROTEIN LIGHT-DEPENDENT SHORT HYPOCOTYLS 10-RELATED
Phytozome	Cicle	*Citrus clementina*	**Ciclev10022295m**		85.6	3.7E-66	205.3	(1 of 9) PF04852 - Protein of unknown function (DUF640)
Phytozome	Cicle	*Citrus clementina*	**Ciclev10029262m**		77.7	1.1E-65	204.5	(1 of 2) PTHR31165:SF10 - PROTEIN LIGHT-DEPENDENT SHORT HYPOCOTYLS 10-RELATED

Database	Acr	Species	Subject ID	Gene name	Id%	E-value	Score	Description
Phytozome	Cicle	*Citrus clementina*	**Ciclev10016755m**		66.9	2.2E-56	180.3	(1 of 9) PF04852 - Protein of unknown function (DUF640)
Phytozome	Cicle	*Citrus clementina*	**Ciclev10016772m**		76.8	1.2E-58	186.0	(1 of 9) PF04852 - Protein of unknown function (DUF640)
Phytozome	Cicle	*Citrus clementina*	**Ciclev10016699m**		75.2	7.2E-60	189.5	(1 of 2) PTHR31165:SF10 - PROTEIN LIGHT-DEPENDENT SHORT HYPOCOTYLS 10-RELATED
Phytozome	Coara	*Coffea arabica*	**evm.model.Scaffold_601.85**		90	1.37e-86	255.373	(1 of 2) PTHR31165:SF6 - PROTEIN LIGHT-DEPENDENT SHORT HYPOCOTYLS 6
Phytozome	Coara	*Coffea arabica*	**evm.model.Scaffold_2016.771**		90	1.42e-86	255.373	(1 of 2) PTHR31165:SF6 - PROTEIN LIGHT-DEPENDENT SHORT HYPOCOTYLS 6
Phytozome	Coara	*Coffea arabica*	**evm.model.Scaffold_315.664**		82	9.40e-75	224.557	(1 of 2) PTHR31165:SF13 - PROTEIN LIGHT-DEPENDENT SHORT HYPOCOTYLS 1-RELATED
Phytozome	Coara	*Coffea arabica*	**evm.model.Scaffold_624.424**		82	9.92e-75	224.557	(1 of 2) PTHR31165:SF13 - PROTEIN LIGHT-DEPENDENT SHORT HYPOCOTYLS 1-RELATED
Phytozome	Coara	*Coffea arabica*	**evm.model.Scaffold_770.140**		75	5.13e-73	218.779	(1 of 3) PTHR31165:SF10 - PROTEIN LIGHT-DEPENDENT SHORT HYPOCOTYLS 10-RELATED

**Database**	**Acr**	**Species**	**Subject ID**	**Gene name**	**Id%**	**E-value**	**Score**	**Description**
Phytozome	Coara	*Coffea arabica*	**evm.model.Scaffold_609.82**		75	1.33e-72	218.009	(1 of 3) PTHR31165:SF10 - PROTEIN LIGHT-DEPENDENT SHORT HYPOCOTYLS 10-RELATED
Phytozome	Coara	*Coffea arabica*	**evm.model.Scaffold_618.644**		74	1.51e-69	210.69	(1 of 12) PF04852 - Protein of unknown function (DUF640) (DUF640)
Phytozome	Coara	*Coffea arabica*	**evm.model.Scaffold_2596.191**		70	3.27e-61	190.274	(1 of 12) PF04852 - Protein of unknown function (DUF640) (DUF640)
Phytozome	Coara	*Coffea arabica*	**evm.model.Scaffold_637.427**		74	4.59e-61	189.889	(1 of 12) PF04852 - Protein of unknown function (DUF640) (DUF640)
Phytozome	Coara	*Coffea arabica*	**evm.model.Scaffold_1777.1**		82	4.79e-61	188.734	(1 of 12) PF04852 - Protein of unknown function (DUF640) (DUF640)
Phytozome	Coara	*Coffea arabica*	**evm.model.Scaffold_635.285**		82	4.79e-61	188.734	(1 of 12) PF04852 - Protein of unknown function (DUF640) (DUF640)
Phytozome	Coara	*Coffea arabica*	**evm.model.Scaffold_2016.367**		82	5.06e-61	188.348	(1 of 12) PF04852 - Protein of unknown function (DUF640) (DUF640)
Phytozome	Coara	*Coffea arabica*	evm.model.Scaffold_618.94		57	4.32e-5	423.578	(1 of 3) PTHR31165:SF10 - PROTEIN LIGHT-DEPENDENT SHORT HYPOCOTYLS 10-RELATED
Marpol	Coorb	*Coleochaete orbicularis*	**gb|GBSL01022649.1**		64.47	1.31 × 10-63	283	TSA: Coleochaete orbicularis

Database	Acr	Species	Subject ID	Gene name	Id%	E-value	Score	Description
								comp25156_c0_seq1 transcribed RNA sequence
NCBI	Dastr	*Datura stramonium*	**MCE0482496.1**		85.51%	6,00E-79	235	hypothetical protein [Datura stramonium]
NCBI	Dastr	*Datura stramonium*	**MCD9637940.1**		79.69%	2,00E-75	225	Protein LIGHT-DEPENDENT SHORT HYPOCOTYLS 4 [Datura stramonium]
NCBI	Dastr	*Datura stramonium*	**MCD7455574.1**		72.87%	2,00E-72	217	Protein LIGHT-DEPENDENT SHORT HYPOCOTYLS 10 [Datura stramonium]
NCBI	Dastr	*Datura stramonium*	**MCD7445813.1**		80.00%	2,00E-71	215	Protein LIGHT-DEPENDENT SHORT HYPOCOTYLS 4 [Datura stramonium]
NCBI	Dastr	*Datura stramonium*	**MCD7470619.1**		72.93%	2,00E-69	209	Protein LIGHT-DEPENDENT SHORT HYPOCOTYLS 7 [Datura stramonium]
NCBI	Dastr	*Datura stramonium*	**MCD9560289.1**		73.60%	3,00E-69	209	Protein LIGHT-DEPENDENT SHORT HYPOCOTYLS 10 [Datura stramonium]
NCBI	Dastr	*Datura stramonium*	**MCD7448299.1**		66.21%	3,00E-69	208	Protein LIGHT-DEPENDENT SHORT HYPOCOTYLS 10 [Datura stramonium]
NCBI	Dastr	*Datura stramonium*	**MCE3215519.1**		79.20%	4,00E-69	209	hypothetical protein [Datura stramonium]
NCBI	Dastr	*Datura stramonium*	**MCD7459833.1**		78.40%	6,00E-69	208	Protein LIGHT-DEPENDENT SHORT HYPOCOTYLS 3 [Datura stramonium]
NCBI	Dastr	*Datura stramonium*	**MCD7468616.1**		73.60%	7,00E-68	206	Protein LIGHT-DEPENDENT SHORT

**Database**	**Acr**	**Species**	**Subject ID**	**Gene name**	**Id%**	**E-value**	**Score**	**Description**
								HYPOCOTYLS 10 [Datura stramonium]
NCBI	Dastr	*Datura stramonium*	MCD9638190.1		58.64%	6,00E-56	175	Protein LIGHT-DEPENDENT SHORT HYPOCOTYLS 1 [Datura stramonium]
NCBI	Dastr	*Datura stramonium*	MCD7446185.1		48.91%	3,00E-35	121	hypothetical protein [Datura stramonium]
NCBI	Dastr	*Datura stramonium*	MCE3049217.1		45.69%	4,00E-26	98.6	hypothetical protein [Datura stramonium]
NCBI	Dastr	*Datura stramonium*	MCD9639141.1		38.46%	1,00E-04	39.7	hypothetical protein [Datura stramonium]
NCBI	Dastr	*Datura stramonium*	MCE3050391.1		71.43%	1,00E-04	40.4	hypothetical protein [Datura stramonium]
Phytozome	Dicom	*Diphasiastrum complanatum*	**Dicom.Y061600.1.p**		79	1.70e-76	229.18	(1 of 5) PF04852 - Protein of unknown function (DUF640) (DUF640)
Phytozome	Dicom	*Diphasiastrum complanatum*	**Dicom.14G033200.1.p**		79	1.70e-76	229.18	(1 of 5) PF04852 - Protein of unknown function (DUF640) (DUF640)
Phytozome	Dicom	*Diphasiastrum complanatum*	**Dicom.10G006000.1.p**		79	2.88e-73	222.631	(1 of 5) PF04852 - Protein of unknown function (DUF640) (DUF640)
Phytozome	Dicom	*Diphasiastrum complanatum*	**Dicom.09G074700.1.p**		75	9.99e-71	214.927	(1 of 5) PF04852 - Protein of unknown function (DUF640) (DUF640)
Phytozome	Dicom	*Diphasiastrum complanatum*	Dicom.01G018900.1.p		78	5.67e-73	221.861	(1 of 5) PF04852 - Protein of unknown function (DUF640) (DUF640)
Phytozome	Eugra	*Eucalyptus grandis*	**Eucgr.H04235.1**		91.5	7.1E-76	230.0	(Egrandis_v1_0.027820m) (1 of 4) PTHR31165:SF13 - PROTEIN LIGHT-DEPENDENT SHORT HYPOCOTYLS 1-RELATED

Database	Acr	Species	Subject ID	Gene name	Id%	E-value	Score	Description
Phytozome	Eugra	*Eucalyptus grandis*	**Eucgr.H03039.1**		93.2	6.2E-72	220.3	(Egrandis_v1_0.027338m) (1 of 4) PTHR31165:SF13 - PROTEIN LIGHT-DEPENDENT SHORT HYPOCOTYLS 1-RELATED
Phytozome	Eugra	*Eucalyptus grandis*	**Eucgr.H00151.1**		86.2	1.1E-70	216.9	(Egrandis_v1_0.054898m) (1 of 4) PTHR31165:SF13 - PROTEIN LIGHT-DEPENDENT SHORT HYPOCOTYLS 1-RELATED
Phytozome	Eugra	*Eucalyptus grandis*	**Eucgr.D01019.1**		85.2	1.2E-65	204.1	(Egrandis_v1_0.044520m) (1 of 12) PF04852 - Protein of unknown function (DUF640) (DUF640)
Phytozome	Eugra	*Eucalyptus grandis*	**Eucgr.B02146.1**		82.3	5.9E-65	201.8	(Egrandis_v1_0.028286m) (1 of 4) PTHR31165:SF13 - PROTEIN LIGHT-DEPENDENT SHORT HYPOCOTYLS 1-RELATED
Phytozome	Eugra	*Eucalyptus grandis*	**Eucgr.K01128.1**		69.1	8.4E-64	198.7	(Egrandis_v1_0.028865m) (1 of 2) PTHR31165:SF10 - PROTEIN LIGHT-DEPENDENT SHORT HYPOCOTYLS 10-RELATED
Phytozome	Eugra	*Eucalyptus grandis*	**Eucgr.F01238.1**		71.7	4.6E-63	197.2	(Egrandis_v1_0.028268m) (1 of 2) PTHR31165:SF9 - PROTEIN LIGHT-DEPENDENT SHORT

**Database**	**Acr**	**Species**	**Subject ID**	**Gene name**	**Id%**	**E-value**	**Score**	**Description**
								HYPOCOTYLS 7-RELATED
Phytozome	Eugra	*Eucalyptus grandis*	**Eucgr.A01916.1**		72.1	6.9E-62	193.7	(Egrandis_v1_0.028721m) (1 of 2) PTHR31165:SF10 - PROTEIN LIGHT-DEPENDENT SHORT HYPOCOTYLS 10-RELATED
Phytozome	Eugra	*Eucalyptus grandis*	**Eucgr.B02224.1**		74.8	4.8E-61	193.4	(Egrandis_v1_0.025740m) (1 of 2) PTHR31165:SF6 - PROTEIN LIGHT-DEPENDENT SHORT HYPOCOTYLS 6
Phytozome	Eugra	*Eucalyptus grandis*	**Eucgr.D00487.1**		71.9	3.2E-59	186.8	(Egrandis_v1_0.028694m) (1 of 12) PF04852 - Protein of unknown function (DUF640) (DUF640)
Phytozome	Eugra	*Eucalyptus grandis*	**Eucgr.F03266.1**		72.5	4.8E-59	186.8	(Egrandis_v1_0.028376m) (1 of 2) PTHR31165:SF9 - PROTEIN LIGHT-DEPENDENT SHORT HYPOCOTYLS 7-RELATED
Phytozome	Eugra	*Eucalyptus grandis*	Eucgr.J01908.1		72.2	4.2E-35	123.6	(Egrandis_v1_0.046958m) (1 of 2) PTHR31165:SF6 - PROTEIN LIGHT-DEPENDENT SHORT HYPOCOTYLS 6
Phytozome	Eusal	*Eutrema salsugineum*	**Thhalv10009726m**		92	2.63e-119	336.265	(1 of 2) PTHR31165:SF6 - PROTEIN LIGHT-DEPENDENT SHORT HYPOCOTYLS 6

Database	Acr	Species	Subject ID	Gene name	Id%	E-value	Score	Description
Phytozome	Eusal	*Eutrema salsugineum*	**Thhalv10017270m**		79	6.30e-83	244.202	(1 of 2) PTHR31165:SF6 - PROTEIN LIGHT-DEPENDENT SHORT HYPOCOTYLS 7
Phytozome	Eusal	*Eutrema salsugineum*	**Thhalv10014789m**		87	9.19e-81	238.424	(1 of 2) PTHR31165:SF6 - PROTEIN LIGHT-DEPENDENT SHORT HYPOCOTYLS 8
Phytozome	Eusal	*Eutrema salsugineum*	**Thhalv10017308m**		74	5.31e-73	218.394	(1 of 2) PTHR31165:SF6 - PROTEIN LIGHT-DEPENDENT SHORT HYPOCOTYLS 9
Phytozome	Eusal	*Eutrema salsugineum*	**Thhalv10021563m**		81	2.50e-70	212.231	(1 of 2) PTHR31165:SF6 - PROTEIN LIGHT-DEPENDENT SHORT HYPOCOTYLS 10
Phytozome	Eusal	*Eutrema salsugineum*	**Thhalv10017162m**		77	1.95e-67	206.453	(1 of 2) PTHR31165:SF6 - PROTEIN LIGHT-DEPENDENT SHORT HYPOCOTYLS 12
Phytozome	Eusal	*Eutrema salsugineum*	**Thhalv10019792m**		70	2.46e-67	204.527	(1 of 2) PTHR31165:SF6 - PROTEIN LIGHT-DEPENDENT SHORT HYPOCOTYLS 13
Phytozome	Eusal	*Eutrema salsugineum*	**Thhalv10004974m**		72	3.57e-67	203.756	(1 of 2) PTHR31165:SF6 - PROTEIN LIGHT-DEPENDENT SHORT HYPOCOTYLS 14
Phytozome	Eusal	*Eutrema salsugineum*	**Thhalv10027091m**		74	2.78e-66	201.83	(1 of 2) PTHR31165:SF6 - PROTEIN LIGHT-DEPENDENT SHORT HYPOCOTYLS 15
Phytozome	Eusal	*Eutrema salsugineum*	**Thhalv10001044m**		73	7.33e-65	197.978	(1 of 2) PTHR31165:SF6 - PROTEIN LIGHT-DEPENDENT SHORT HYPOCOTYLS 16
Phytozome	Eusal	*Eutrema salsugineum*	Thhalv10017220m		77	3.49e-68	207.608	(1 of 2) PTHR31165:SF6 - PROTEIN LIGHT-

Database	Acr	Species	Subject ID	Gene name	Id%	E-value	Score	Description
								DEPENDENT SHORT HYPOCOTYLS 11
Phytozome	Eusal	*Eutrema salsugineum*	Thhalv10027276m		26	2.34e-5	442.838	(1 of 2) PTHR31165:SF6 - PROTEIN LIGHT-DEPENDENT SHORT HYPOCOTYLS 17
Phytozome	Glmax	*Glycine max*	**Glyma.11G165700.1.p**		86	5.09e-83	247.284	(1 of 3) PTHR31165:SF6 - PROTEIN LIGHT-DEPENDENT SHORT HYPOCOTYLS 6
Phytozome	Glmax	*Glycine max*	**Glyma.14G197300.1.p**		88	6.75e-80	238.424	(1 of 1) PTHR31165:SF14 - PROTEIN LIGHT-DEPENDENT SHORT HYPOCOTYLS 5
Phytozome	Glmax	*Glycine max*	**Glyma.02G230200.1.p**		89	1.33e-78	235.343	(1 of 3) PTHR31165:SF6 - PROTEIN LIGHT-DEPENDENT SHORT HYPOCOTYLS 6
Phytozome	Glmax	*Glycine max*	**Glyma.18G058600.1.p**		87	4.59e-77	231.876	(1 of 3) PTHR31165:SF6 - PROTEIN LIGHT-DEPENDENT SHORT HYPOCOTYLS 6
Phytozome	Glmax	*Glycine max*	**Glyma.05G123400.1.p**		79	2.93e-75	227.639	(1 of 25) PF04852 - Protein of unknown function (DUF640) (DUF640)
Phytozome	Glmax	*Glycine max*	**Glyma.12G229500.1.p**		76	1.08e-71	216.468	(1 of 2) PTHR31165:SF9 - PROTEIN LIGHT-DEPENDENT SHORT HYPOCOTYLS 7-RELATED
Phytozome	Glmax	*Glycine max*	**Glyma.13G270300.1.p**		76	3.70e-71	215.698	(1 of 2) PTHR31165:SF9 - PROTEIN LIGHT-DEPENDENT SHORT HYPOCOTYLS 7-RELATED

Database	Acr	Species	Subject ID	Gene name	Id%	E-value	Score	Description
Phytozome	Glmax	*Glycine max*	**Glyma.12G210800.1.p**		72	3.79e-71	214.927	(1 of 4) PTHR31165:SF10 - PROTEIN LIGHT-DEPENDENT SHORT HYPOCOTYLS 10-RELATED
Phytozome	Glmax	*Glycine max*	**Glyma.10G181200.1.p**		78	8.95e-71	214.927	(1 of 2) PTHR31165:SF13 - PROTEIN LIGHT-DEPENDENT SHORT HYPOCOTYLS 1-RELATED
Phytozome	Glmax	*Glycine max*	**Glyma.20G209200.1.p**		81	6.63e-70	212.231	(1 of 2) PTHR31165:SF13 - PROTEIN LIGHT-DEPENDENT SHORT HYPOCOTYLS 1-RELATED
Phytozome	Glmax	*Glycine max*	**Glyma.06G277400.1.p**		72	1.06e-69	211.46	(1 of 4) PTHR31165:SF10 - PROTEIN LIGHT-DEPENDENT SHORT HYPOCOTYLS 10-RELATED
Phytozome	Glmax	*Glycine max*	**Glyma.10G037600.1.p**		80	1.78e-68	209.149	(1 of 25) PF04852 - Protein of unknown function (DUF640) (DUF640)
Phytozome	Glmax	*Glycine max*	**Glyma.13G229700.1.p**		73	2.61e-68	207.994	(1 of 25) PF04852 - Protein of unknown function (DUF640) (DUF640)
Phytozome	Glmax	*Glycine max*	**Glyma.19G165300.1.p**		79	4.72e-68	207.994	(1 of 25) PF04852 - Protein of unknown function (DUF640) (DUF640)
Phytozome	Glmax	*Glycine max*	**Glyma.03G164000.1.p**		79	5.32e-68	207.994	(1 of 25) PF04852 - Protein of unknown

**Database**	**Acr**	**Species**	**Subject ID**	**Gene name**	**Id%**	**E-value**	**Score**	**Description**
								function (DUF640) (DUF640)
Phytozome	Glmax	*Glycine max*	**Glyma.15G082800.1.p**		72	1.12e-67	206.068	(1 of 25) PF04852 - Protein of unknown function (DUF640) (DUF640)
Phytozome	Glmax	*Glycine max*	**Glyma.06G315400.1.p**		79	1.63e-67	206.838	(1 of 25) PF04852 - Protein of unknown function (DUF640) (DUF640)
Phytozome	Glmax	*Glycine max*	**Glyma.20G223100.1.p**		79	2.83e-66	202.986	(1 of 25) PF04852 - Protein of unknown function (DUF640) (DUF640)
Phytozome	Glmax	*Glycine max*	**Glyma.10G166100.1.p**		79	3.86e-66	202.216	(1 of 25) PF04852 - Protein of unknown function (DUF640) (DUF640)
Phytozome	Glmax	*Glycine max*	**Glyma.19G248300.1.p**		76	7.03e-65	199.134	(1 of 25) PF04852 - Protein of unknown function (DUF640) (DUF640)
Phytozome	Glmax	*Glycine max*	**Glyma.03G250900.1.p**		74	2.47e-63	195.282	(1 of 25) PF04852 - Protein of unknown function (DUF640) (DUF640)
Phytozome	Glmax	*Glycine max*	**Glyma.12G128100.1.p**		72	1.20e-62	193.356	(1 of 4) PTHR31165:SF10 - PROTEIN LIGHT-DEPENDENT SHORT HYPOCOTYLS 10-RELATED
Phytozome	Glmax	*Glycine max*	**Glyma.08G116100.1.p**		72	2.80e-59	184.496	(1 of 25) PF04852 - Protein of unknown function (DUF640) (DUF640)
Phytozome	Glmax	*Glycine max*	Glyma.08G078500.1.p		79	2.42e-75	228.024	(1 of 25) PF04852 - Protein of unknown

Database	Acr	Species	Subject ID	Gene name	Id%	E-value	Score	Description
								function (DUF640) (DUF640)
Phytozome	Glmax	*Glycine max*	Glyma.13G290600.1.p		58	1.53e-56	177.178	(1 of 4) PTHR31165:SF10 - PROTEIN LIGHT-DEPENDENT SHORT HYPOCOTYLS 10-RELATED
Phytozome	Heann	*Helianthus annuus*	**HanXRQChr08g0229201**		89	2.98e-82	243.432	Protein of unknown function (DUF640)
Phytozome	Heann	*Helianthus annuus*	**HanXRQChr13g0422001**		81	3.64e-76	227.639	Putative ALOG domain
Phytozome	Heann	*Helianthus annuus*	**HanXRQChr13g0390271**		72	2.29e-71	215.312	Protein of unknown function (DUF640)
Phytozome	Heann	*Helianthus annuus*	**HanXRQChr15g0467771**		71	1.62e-70	213.386	Putative ALOG domain
Phytozome	Heann	*Helianthus annuus*	**HanXRQChr14g0442741**		75	3.76e-70	211.846	Putative ALOG domain
Phytozome	Heann	*Helianthus annuus*	**HanXRQChr03g0091471**		80	2.83e-69	209.92	Protein of unknown function (DUF640)
Phytozome	Heann	*Helianthus annuus*	**HanXRQChr01g0022571**		73	9.56e-69	210.305	Putative ALOG domain
Phytozome	Heann	*Helianthus annuus*	**HanXRQChr04g0111361**		77	5.75e-67	203.756	Probable protein G1-like1
Phytozome	Heann	*Helianthus annuus*	**HanXRQChr09g0254321**		78	7.83e-65	198.749	Putative ALOG domain
Phytozome	Heann	*Helianthus annuus*	**HanXRQChr04g0110511**		74	3.05e-63	194.126	Putative ALOG domain
Phytozome	Heann	*Helianthus annuus*	**HanXRQChr15g0488051**		72	1.24e-60	186.808	Putative ALOG domain
Phytozome	Heann	*Helianthus annuus*	**HanXRQChr04g0105371**		66	1.82e-59	183.726	Putative ALOG domain
Phytozome	Heann	*Helianthus annuus*	**HanXRQChr15g0488041**		71	3.79e-59	184.882	Putative ALOG domain
Phytozome	Heann	*Helianthus annuus*	HanXRQChr17g0537841		83	2.43e-6	473.654	Putative PB1 domain; Zinc finger, Dof-type; ALOG domain

Database	Acr	Species	Subject ID	Gene name	Id%	E-value	Score	Description
Phytozome	Horvu	*Hordeum vulgare*	**HORVU1Hr1G060250.1**		86	1.01e-73	226.098	(1 of 1) PTHR31165:SF6 - PROTEIN LIGHT-DEPENDENT SHORT HYPOCOTYLS 6
Phytozome	Horvu	*Hordeum vulgare*	**HORVU3Hr1G088000.1**		73	2.40e-73	222.631	(1 of 9) PF04852 - Protein of unknown function (DUF640) (DUF640)
Phytozome	Horvu	*Hordeum vulgare*	**HORVU2Hr1G089190.3**		80	7.87e-71	215.698	(1 of 9) PF04852 - Protein of unknown function (DUF640) (DUF640)
Phytozome	Horvu	*Hordeum vulgare*	**HORVU1Hr1G070480.1**		83	2.27e-69	213.386	(1 of 9) PF04852 - Protein of unknown function (DUF640) (DUF640)
Phytozome	Horvu	*Hordeum vulgare*	**HORVU6Hr1G088790.1**		82	2.99e-69	213.772	(1 of 9) PF04852 - Protein of unknown function (DUF640) (DUF640)
Phytozome	Horvu	*Hordeum vulgare*	**HORVU1Hr1G041240.5**		79	2.49e-68	210.305	(1 of 9) PF04852 - Protein of unknown function (DUF640) (DUF640)
Phytozome	Horvu	*Hordeum vulgare*	HORVU6Hr1G058340.3		78	1.97e-70	214.927	(1 of 9) PF04852 - Protein of unknown function (DUF640) (DUF640)
Phytozome	Horvu	*Hordeum vulgare*	HORVU7Hr1G106960.3		75	2.65e-67	209.149	(1 of 9) PF04852 - Protein of unknown function (DUF640) (DUF640)
Phytozome	Horvu	*Hordeum vulgare*	HORVU2Hr1G060890.2		63	1.38e-57	184.111	(1 of 9) PF04852 - Protein of unknown function (DUF640) (DUF640)
Phytozome	Hyque	*Hydrangea quercifolia*	**Hyque.04G128200.1.p**		91	7.14e-79	234.958	(1 of 1) PTHR31165//PTHR31165:SF6 - FAMILY NOT NAMED // PROTEIN LIGHT-DEPENDENT SHORT HYPOCOTYLS 6
Phytozome	Hyque	*Hydrangea quercifolia*	**Hyque.12G103400.1.p**		68	8.33e-75	223.402	(1 of 1) PTHR31165:SF10 -

Database	Acr	Species	Subject ID	Gene name	Id%	E-value	Score	Description
								PROTEIN LIGHT-DEPENDENT SHORT HYPOCOTYLS 10-RELATED
Phytozome	Hyque	*Hydrangea quercifolia*	**Hyque.01G163500.1.p**		73	8.98e-71	213.386	(1 of 8) PF04852 - Protein of unknown function (DUF640) (DUF640)
Phytozome	Hyque	*Hydrangea quercifolia*	**Hyque.10G106100.1.p**		82	4.42e-70	211.846	(1 of 2) PTHR31165//PTHR31165:SF13 - FAMILY NOT NAMED // PROTEIN LIGHT-DEPENDENT SHORT HYPOCOTYLS 1-RELATED
Phytozome	Hyque	*Hydrangea quercifolia*	**Hyque.12G041300.1.p**		71	5.27e-70	211.46	(1 of 1) PTHR31165//PTHR31165:SF9 - FAMILY NOT NAMED // PROTEIN LIGHT-DEPENDENT SHORT HYPOCOTYLS 7-RELATED
Phytozome	Hyque	*Hydrangea quercifolia*	**Hyque.04G163200.1.p**		80	3.10e-68	207.223	(1 of 8) PF04852 - Protein of unknown function (DUF640) (DUF640)
Phytozome	Hyque	*Hydrangea quercifolia*	**Hyque.04G017200.1.p**		73	4.59e-67	204.527	(1 of 2) PTHR31165:SF13 - PROTEIN LIGHT-DEPENDENT SHORT HYPOCOTYLS 1-RELATED
Phytozome	Hyque	*Hydrangea quercifolia*	**Hyque.01G117800.1.p**		72	9.21e-62	192.971	(1 of 8) PF04852 - Protein of unknown function (DUF640) (DUF640)
Phytozome	Hyque	*Hydrangea quercifolia*	Hyque.16G027200.1.p		38	9.36e-2	338.834	(1 of 5) PTHR24098:SF5 - ACYL-COA N-ACYLTRANSFERASE WITH RING/FYVE/PHD-TYPE

Database	Acr	Species	Subject ID	Gene name	Id%	E-value	Score	Description
								ZINC FINGER DOMAIN-CONTAINING PROTEIN
Phytozome	Litul	*Liriodendron tulipifera*	**Litul.15G035400.1.p**		87	2.10e-77	231.876	(1 of 2) PTHR31165//PTHR31165:SF6 - FAMILY NOT NAMED // PROTEIN LIGHT-DEPENDENT SHORT HYPOCOTYLS 6
Phytozome	Litul	*Liriodendron tulipifera*	**Litul.07G035800.1.p**		90	2.95e-77	231.106	(1 of 2) PTHR31165//PTHR31165:SF6 - FAMILY NOT NAMED // PROTEIN LIGHT-DEPENDENT SHORT HYPOCOTYLS 6
Phytozome	Litul	*Liriodendron tulipifera*	**Litul.08G065900.1.p**		81	1.97e-74	223.402	(1 of 2) PTHR31165:SF13 - PROTEIN LIGHT-DEPENDENT SHORT HYPOCOTYLS 1-RELATED
Phytozome	Litul	*Liriodendron tulipifera*	**Litul.03G192600.1.p**		70	7.36e-72	216.083	(1 of 2) PTHR31165:SF10 - PROTEIN LIGHT-DEPENDENT SHORT HYPOCOTYLS 10-RELATED
Phytozome	Litul	*Liriodendron tulipifera*	**Litul.08G120900.1.p**		72	8.85e-70	211.075	(1 of 2) PTHR31165:SF10 - PROTEIN LIGHT-DEPENDENT SHORT HYPOCOTYLS 10-RELATED
Phytozome	Litul	*Liriodendron tulipifera*	**Litul.03G125300.1.p**		82	1.43e-69	210.69	(1 of 2) PTHR31165//PTHR3116

Database	Acr	Species	Subject ID	Gene name	Id%	E-value	Score	Description
								5:SF13 - FAMILY NOT NAMED // PROTEIN LIGHT-DEPENDENT SHORT HYPOCOTYLS 1-RELATED
Phytozome	Litul	*Liriodendron tulipifera*	**Litul.01G099700.1.p**		80	2.76e-69	211.46	(1 of 7) PF04852 - Protein of unknown function (DUF640) (DUF640)
Phytozome	Maesc	*Manihot esculenta*	**Manes.18G142500.1.p**		90	1.29e-79	238.039	(1 of 2) PTHR31165:SF6 - PROTEIN LIGHT-DEPENDENT SHORT HYPOCOTYLS 6
Phytozome	Maesc	*Manihot esculenta*	**Manes.02G216200.1.p**		86	1.41e-76	229.95	(1 of 2) PTHR31165:SF6 - PROTEIN LIGHT-DEPENDENT SHORT HYPOCOTYLS 6
Phytozome	Maesc	*Manihot esculenta*	**Manes.02G090700.1.p**		75	1.69e-73	219.935	(1 of 2) PTHR31165:SF10 - PROTEIN LIGHT-DEPENDENT SHORT HYPOCOTYLS 10-RELATED
Phytozome	Maesc	*Manihot esculenta*	**Manes.15G094500.1.p**		78	2.00e-72	217.624	(1 of 2) PTHR31165:SF9 - PROTEIN LIGHT-DEPENDENT SHORT HYPOCOTYLS 7-RELATED
Phytozome	Maesc	*Manihot esculenta*	**Manes.03G101700.1.p**		75	7.95e-72	215.698	(1 of 16) PF04852 - Protein of unknown function (DUF640)
Phytozome	Maesc	*Manihot esculenta*	**Manes.16G039700.1.p**		72	7.84e-70	211.075	(1 of 16) PF04852 - Protein of unknown function (DUF640) (DUF640)
Phytozome	Maesc	*Manihot esculenta*	**Manes.15G041500.1.p**		80	1.20e-69	211.075	(1 of 2) PTHR31165:SF13 - PROTEIN LIGHT-DEPENDENT SHORT

Database	Acr	Species	Subject ID	Gene name	Id%	E-value	Score	Description
								HYPOCOTYLS 1-RELATED
Phytozome	Maesc	*Manihot esculenta*	**Manes.14G126500.1.p**		83	1.92e-69	211.846	(1 of 16) PF04852 - Protein of unknown function (DUF640) (DUF640)
Phytozome	Maesc	*Manihot esculenta*	**Manes.06G054300.1.p**		82	2.34e-69	211.46	(1 of 16) PF04852 - Protein of unknown function (DUF640) (DUF640)
Phytozome	Maesc	*Manihot esculenta*	**Manes.03G163600.1.p**		79	4.61e-69	209.92	(1 of 16) PF04852 - Protein of unknown function (DUF640)
Phytozome	Maesc	*Manihot esculenta*	**Manes.17G055900.1.p**		73	1.88e-68	207.608	(1 of 16) PF04852 - Protein of unknown function (DUF640)
Phytozome	Maesc	*Manihot esculenta*	**Manes.09G148200.1.p**		77	1.63e-67	205.297	(1 of 16) PF04852 - Protein of unknown function (DUF640) (DUF640)
Phytozome	Maesc	*Manihot esculenta*	**Manes.13G035100.1.p**		78	1.47e-66	202.986	(1 of 16) PF04852 - Protein of unknown function (DUF640) (DUF640)
Phytozome	Maesc	*Manihot esculenta*	**Manes.12G033200.1.p**		78	5.55e-66	201.445	(1 of 16) PF04852 - Protein of unknown function (DUF640)
Phytozome	Maesc	*Manihot esculenta*	**Manes.08G139300.1.p**		78	2.77e-65	199.519	(1 of 16) PF04852 - Protein of unknown function (DUF640) (DUF640)
Phytozome	Maesc	*Manihot esculenta*	**Manes.01G132000.1.p**		73	1.05e-60	187.578	(1 of 2) PTHR31165:SF10 - PROTEIN LIGHT-DEPENDENT SHORT HYPOCOTYLS 10-RELATED

Database	Acr	Species	Subject ID	Gene name	Id%	E-value	Score	Description
Phytozome	Mapol	*Marchantia polymorpha*	**Mapoly0028s0118.1**	MpLOS1	80.9	5.7E-67	209.9	(1 of 2) PF04852 - Protein of unknown function (DUF640)
Phytozome	Mapol	*Marchantia polymorpha*	**Mapoly0221s0004.1**	MpLOS2	71.1	4.8E-55	180.3	(1 of 2) PF04852 - Protein of unknown function (DUF640)
Phytozome	Metru	*Medicago truncatula*	**Medtr3g031830.1**		76.9	9.2E-65	203.0	(1 of 2) PTHR31165:SF6 - PROTEIN LIGHT-DEPENDENT SHORT HYPOCOTYLS 6
Phytozome	Metru	*Medicago truncatula*	**Medtr2g092950.1**		74.6	6.6E-59	186.0	(1 of 3) PTHR31165:SF10 - PROTEIN LIGHT-DEPENDENT SHORT HYPOCOTYLS 10-RELATED
Phytozome	Metru	*Medicago truncatula*	**Medtr7g115700.1**		76.5	1.1E-61	193.4	(1 of 1) PTHR31165:SF9 - PROTEIN LIGHT-DEPENDENT SHORT HYPOCOTYLS 7-RELATED
Phytozome	Metru	*Medicago truncatula*	**Medtr5g072510.1**		74.1	8.7E-67	208.0	(1 of 1) PTHR31165:SF9 - PROTEIN LIGHT-DEPENDENT SHORT HYPOCOTYLS 7-RELATED
Phytozome	Metru	*Medicago truncatula*	**Medtr1g442860.1**		87.2	7.7E-67	208.0	(1 of 3) PTHR31165:SF10 - PROTEIN LIGHT-DEPENDENT SHORT HYPOCOTYLS 10-RELATED
Phytozome	Metru	*Medicago truncatula*	**Medtr1g069825.1**		83.8	8.5E-68	210.7	(1 of 15) PF04852 - Protein of unknown function (DUF640)
Phytozome	Metru	*Medicago truncatula*	**Medtr1g075990.1**		74.1	6,00E-72	220.3	(1 of 4) PTHR31165:SF13 - PROTEIN LIGHT-

Database	Acr	Species	Subject ID	Gene name	Id%	E-value	Score	Description
								DEPENDENT SHORT HYPOCOTYLS 1-RELATED
Phytozome	Metru	*Medicago truncatula*	**Medtr1g080210.1**		87.2	4.4E-70	214.9	(1 of 15) PF04852 - Protein of unknown function (DUF640)
Phytozome	Metru	*Medicago truncatula*	**Medtr4g079930.1**		70.4	2.6E-58	184.9	(1 of 3) PTHR31165:SF10 - PROTEIN LIGHT-DEPENDENT SHORT HYPOCOTYLS 10-RELATED
Phytozome	Metru	*Medicago truncatula*	**Medtr4g094428.1**		79	1.1E-67	210.7	(1 of 15) PF04852 - Protein of unknown function (DUF640) (DUF640)
Phytozome	Metru	*Medicago truncatula*	**Medtr2g076400.1**		189.1	6.6E-60	189.1	(1 of 1) PTHR31165:SF9 - PROTEIN LIGHT-DEPENDENT SHORT HYPOCOTYLS 7-RELATED
Phytozome	Metru	*Medicago truncatula*	**Medtr2g016110.2**		74.4	7.6E-57	181.4	(1 of 15) PF04852 - Protein of unknown function (DUF640)
Phytozome	Metru	*Medicago truncatula*	**Medtr8g086350.1**		71.2	2.8E-53	172.2	(1 of 3) PTHR31165:SF10 - PROTEIN LIGHT-DEPENDENT SHORT HYPOCOTYLS 10-RELATED
Phytozome	Metru	*Medicago truncatula*	**Medtr7g097030.2**		83.1	2,00E-70	217.2	(1 of 4) PTHR31165:SF13 - PROTEIN LIGHT-DEPENDENT SHORT HYPOCOTYLS 1-RELATED

Database	Acr	Species	Subject ID	Gene name	Id%	E-value	Score	Description
PhycoCosm	Meend	*Mesotaenium endlicherianum SAG 12.97*	**ME000205S03022**		33.72	1.93E-052	473	-
PhycoCosm	Mekra	*Mesotaenium kramstae Lemmermann*	**jgi|Meskra657_3|2979558|fgenesh1_kg.20_#_758_#_TRINITY_DN46326_c0_g1_i1**		25.62	4.29E-055	493	-
Phytozome	Migut	*Mimulus guttatus*	**Migut.H01303.1**		74.1	7.6E-69	212.6	(mgv1a013748m.g) (1 of 9) PF04852 - Protein of unknown function (DUF640) (DUF640)
Phytozome	Migut	*Mimulus guttatus*	**Migut.D00435.1**		84	8.3E-69	211.8	(mgv1a020501m.g) (1 of 2) PTHR31165:SF13 - PROTEIN LIGHT-DEPENDENT SHORT HYPOCOTYLS 1-RELATED
Phytozome	Migut	*Mimulus guttatus*	**Migut.D00854.1**		75	1.6E-64	201.1	(mgv1a021801m.g) (1 of 1) PTHR31165:SF6 - PROTEIN LIGHT-DEPENDENT SHORT HYPOCOTYLS 6
Phytozome	Migut	*Mimulus guttatus*	**Migut.H01433.1**		75.8	1.8E-61	192.2	(mgv1a022623m.g) (1 of 9) PF04852 - Protein of unknown function (DUF640) (DUF640)
Phytozome	Migut	*Mimulus guttatus*	**Migut.M01038.1**		67.8	2.3E-61	192.6	(mgv1a022457m.g) (1 of 9) PF04852 - Protein of unknown function (DUF640) (DUF640)
Phytozome	Migut	*Mimulus guttatus*	**Migut.M00263.1**		69.3	9.9E-60	187.6	(mgv1a021620m.g) (1 of 2) PTHR31165:SF10 - PROTEIN LIGHT-DEPENDENT SHORT HYPOCOTYLS 10-RELATED
Phytozome	Migut	*Mimulus guttatus*	**Migut.M00732.1**		75.2	3.6E-59	187.2	(1 of 1) PTHR31165:SF9 - PROTEIN LIGHT-DEPENDENT SHORT

Database	Acr	Species	Subject ID	Gene name	Id%	E-value	Score	Description
								HYPOCOTYLS 7-RELATED
Phytozome	Migut	*Mimulus guttatus*	**Migut.H02151.1**		70.4	3.7E-56	178.7	(1 of 2) PTHR31165:SF10 - PROTEIN LIGHT-DEPENDENT SHORT HYPOCOTYLS 10-RELATED
Phytozome	Migut	*Mimulus guttatus*	**Migut.N00276.1**		85.7	1.3E-32	116.7	(1 of 2) PTHR31165:SF13 - PROTEIN LIGHT-DEPENDENT SHORT HYPOCOTYLS 1-RELATED
NCI	Nenuc	*Nelumbo nucifera*	**XP_010264562.1**		76.73	3,29E-80	243	PREDICTED: protein LIGHT-DEPENDENT SHORT HYPOCOTYLS 5
NCI	Nenuc	*Nelumbo nucifera*	**XP_010257059.1**		75.30	3,87E-78	238	PREDICTED: protein LIGHT-DEPENDENT SHORT HYPOCOTYLS 6-like
NCI	Nenuc	*Nelumbo nucifera*	**XP_010246796.1**		81.95	1,75E-73	224	PREDICTED: protein LIGHT-DEPENDENT SHORT HYPOCOTYLS 4-like
NCI	Nenuc	*Nelumbo nucifera*	**XP_010262338.1**		81.82	4,55E-73	225	PREDICTED: protein LIGHT-DEPENDENT SHORT HYPOCOTYLS 4-like isoform X1
NCI	Nenuc	*Nelumbo nucifera*	**XP_010262340.1**		81.82	9,67E-73	223	PREDICTED: protein LIGHT-DEPENDENT SHORT HYPOCOTYLS 3-like isoform X2
NCI	Nenuc	*Nelumbo nucifera*	**XP_010279129.1**		75.76	8,75E-71	218	PREDICTED: protein LIGHT-DEPENDENT SHORT HYPOCOTYLS 10-like

Database	Acr	Species	Subject ID	Gene name	Id%	E-value	Score	Description
NCI	Nenuc	*Nelumbo nucifera*	**XP_010277024.1**		74.24	3,81E-69	214	PREDICTED: protein LIGHT-DEPENDENT SHORT HYPOCOTYLS 10-like
SOL Genomics	Niatt	*Nicotiana attenuata*	**NIATv7_g26636.t1**		83.12	2,00E-89	262	Protein LIGHT-DEPENDENT SHORT HYPOCOTYLS 1Length=194
SOL Genomics	Niatt	*Nicotiana attenuata*	**NIATv7_g32746.t1**		74.05	6,00E-81	240	Protein LIGHT-DEPENDENT SHORT HYPOCOTYLS 2Length=189
SOL Genomics	Niatt	*Nicotiana attenuata*	**NIATv7_g00415.t1**		84.44	2,00E-73	221	Protein LIGHT-DEPENDENT SHORT HYPOCOTYLS 4Length=197
SOL Genomics	Niatt	*Nicotiana attenuata*	**NIATv7_g25647.t1**		65.36	6,00E-72	217	Protein LIGHT-DEPENDENT SHORT HYPOCOTYLS 10Length=177
SOL Genomics	Niatt	*Nicotiana attenuata*	**NIATv7_g56946.t1**		65.36	2,00E-71	216	Protein LIGHT-DEPENDENT SHORT HYPOCOTYLS 10Length=181
SOL Genomics	Niatt	*Nicotiana attenuata*	**NIATv7_g09836.t1**		74.42	3,00E-70	213	Protein LIGHT-DEPENDENT SHORT HYPOCOTYLS 10Length=177
SOL Genomics	Niatt	*Nicotiana attenuata*	**NIATv7_g35584.t1**		76.74	4,00E-70	213	Protein LIGHT-DEPENDENT SHORT HYPOCOTYLS 10Length=196
SOL Genomics	Niatt	*Nicotiana attenuata*	**NIATv7_g23960.t1**		77.78	4,00E-70	212	Protein LIGHT-DEPENDENT SHORT HYPOCOTYLS 4Length=178
SOL Genomics	Niatt	*Nicotiana attenuata*	**NIATv7_g26008.t1**		65.58	2,00E-68	209	Protein LIGHT-DEPENDENT SHORT

Database	Acr	Species	Subject ID	Gene name	Id%	E-value	Score	Description
								HYPOCOTYLS 10Length=188
SOL Genomics	Niatt	*Nicotiana attenuata*	**NIATv7_g10870.t1**		75.00	2,00E-67	207	Protein LIGHT-DEPENDENT SHORT HYPOCOTYLS 10Length=207
SOL Genomics	Niatt	*Nicotiana attenuata*	**NIATv7_g27404.t1**		85.60	3,00E-67	205	Protein LIGHT-DEPENDENT SHORT HYPOCOTYLS 3Length=179
SOL Genomics	Niatt	*Nicotiana attenuata*	**NIATv7_g21067.t1**		73.94	9,00E-67	206	Protein G1-like9Length=235
SOL Genomics	Niatt	*Nicotiana attenuata*	**NIATv7_g12718.t1**		70.77	5,00E-66	202	Protein LIGHT-DEPENDENT SHORT HYPOCOTYLS 7Length=189
SOL Genomics	Niatt	*Nicotiana attenuata*	**NIATv7_g27811.t1**		83.20	4,00E-65	200	Protein LIGHT-DEPENDENT SHORT HYPOCOTYLS 3Length=190
SOL Genomics	Niben	*Nicotiana benthamiana*	**Niben101Scf02724g04006.1**		83.55	3,00E-90	264	sp|Q9M836|LSH2_ARATH ***- Protein LIGHT-DEPENDENT SHORT HYPOCOTYLS 2 IPR006936 (ALOG domain) Length=195
SOL Genomics	Niben	*Nicotiana benthamiana*	**Niben101Scf03709g00007.1**		74.72	2,00E-89	263	sp|Q6NNI3|LSH1_ARATH ***- Protein LIGHT-DEPENDENT SHORT HYPOCOTYLS 1 IPR006936 (ALOG domain) Length=195
SOL Genomics	Niben	*Nicotiana benthamiana*	**Niben101Scf09658g00030.1**		77.08	1,00E-79	238	sp|Q9M836|LSH2_ARATH ***- Protein LIGHT-DEPENDENT SHORT HYPOCOTYLS 2 IPR006936 (ALOG domain) Length=196

**Database**	**Acr**	**Species**	**Subject ID**	**Gene name**	**Id%**	**E-value**	**Score**	**Description**
SOL Genomics	Niben	*Nicotiana benthamiana*	**Niben101Scf03435g00008.1**		76.19	1,00E-78	235	sp|Q6NNI3|LSH1_ARATH ***- Protein LIGHT-DEPENDENT SHORT HYPOCOTYLS 1 IPR006936 (ALOG domain) Length=208
SOL Genomics	Niben	*Nicotiana benthamiana*	**Niben101Scf00944g03001.1**		75.89	3,00E-73	221	sp|O82268|LSH3_ARATH ***- Protein LIGHT-DEPENDENT SHORT HYPOCOTYLS 3 IPR006936 (ALOG domain) Length=188
SOL Genomics	Niben	*Nicotiana benthamiana*	**Niben101Scf02873g02011.1**		65.36	1,00E-71	217	sp|Q9S7R3|LSH10_ARATH ***- Protein LIGHT-DEPENDENT SHORT HYPOCOTYLS 10 IPR006936 (ALOG domain) Length=176
SOL Genomics	Niben	*Nicotiana benthamiana*	**Niben101Scf06183g00004.1**		64.74	4,00E-71	215	sp|Q9S7R3|LSH10_ARATH ***- Protein LIGHT-DEPENDENT SHORT HYPOCOTYLS 10 IPR006936 (ALOG domain) Length=176
SOL Genomics	Niben	*Nicotiana benthamiana*	**Niben101Scf14526g02015.1**		76.74	1,00E-70	214	sp|Q9S7R3|LSH10_ARATH ***- Protein LIGHT-DEPENDENT SHORT HYPOCOTYLS 10 IPR006936 (ALOG domain) Length=196
SOL Genomics	Niben	*Nicotiana benthamiana*	**Niben101Scf07352g03001.1**		74.42	6,00E-70	212	sp|Q9S7R3|LSH10_ARATH ***- Protein LIGHT-DEPENDENT SHORT HYPOCOTYLS 10 IPR006936 (ALOG domain) Length=177
SOL Genomics	Niben	*Nicotiana benthamiana*	**Niben101Scf06183g01007.1**		63.46	8,00E-70	212	sp|Q9S7R3|LSH10_ARATH ***- Protein LIGHT-

Database	Acr	Species	Subject ID	Gene name	Id%	E-value	Score	Description
								DEPENDENT SHORT HYPOCOTYLS 10 IPR006936 (ALOG domain) Length=176
SOL Genomics	Niben	*Nicotiana benthamiana*	**Niben101Scf00271g05016.1**		77.60	3,00E-69	211	sp|Q9LMK2|LSH6_ARATH ***- Protein LIGHT-DEPENDENT SHORT HYPOCOTYLS 6 IPR006936 (ALOG domain) Length=178
SOL Genomics	Niben	*Nicotiana benthamiana*	**Niben101Scf08374g04001.1**		73.64	3,00E-69	211	sp|Q9S7R3|LSH10_ARATH ***- Protein LIGHT-DEPENDENT SHORT HYPOCOTYLS 10 IPR006936 (ALOG domain) Length=177
SOL Genomics	Niben	*Nicotiana benthamiana*	**Niben101Scf17872g00009.1**		74.24	3,00E-69	210	sp|Q9S7R3|LSH10_ARATH ***- Protein LIGHT-DEPENDENT SHORT HYPOCOTYLS 10 IPR006936 (ALOG domain) Length=175
SOL Genomics	Niben	*Nicotiana benthamiana*	**Niben101Scf04236g00006.1**		73.28	7,00E-69	210	sp|Q9S7R3|LSH10_ARATH ***- Protein LIGHT-DEPENDENT SHORT HYPOCOTYLS 10 IPR006936 (ALOG domain) Length=192
SOL Genomics	Niben	*Nicotiana benthamiana*	**Niben101Scf05469g03004.1**		73.48	3,00E-68	209	sp|Q9S7R3|LSH10_ARATH ***- Protein LIGHT-DEPENDENT SHORT HYPOCOTYLS 10 IPR006936 (ALOG domain) Length=188
SOL Genomics	Niben	*Nicotiana benthamiana*	**Niben101Scf02525g01005.1**		85.16	2,00E-67	206	sp|Q9LW68|LSH4_ARATH ***- Protein LIGHT-DEPENDENT SHORT HYPOCOTYLS 4

Database	Acr	Species	Subject ID	Gene name	Id%	E-value	Score	Description
								IPR006936 (ALOG domain) Length=175
SOL Genomics	Niben	*Nicotiana benthamiana*	**Niben101Scf02243g04001.1**		85.60	2,00E-67	207	sp|O82268|LSH3_ARATH *-*- Protein LIGHT-DEPENDENT SHORT HYPOCOTYLS 3 IPR006936 (ALOG domain) Length=198
SOL Genomics	Niben	*Nicotiana benthamiana*	**Niben101Scf00501g03004.1**		84.92	4,00E-67	205	sp|Q9LW68|LSH4_ARATH ***- Protein LIGHT-DEPENDENT SHORT HYPOCOTYLS 4 IPR006936 (ALOG domain) Length=171
SOL Genomics	Niben	*Nicotiana benthamiana*	**Niben101Scf12092g00015.1**		85.60	7,00E-67	205	sp|O82268|LSH3_ARATH ***- Protein LIGHT-DEPENDENT SHORT HYPOCOTYLS 3 IPR006936 (ALOG domain) Length=188
SOL Genomics	Niben	*Nicotiana benthamiana*	**Niben101Scf06311g00001.1**		73.94	2,00E-66	206	sp|Q9LMK2|LSH6_ARATH *-*- Protein LIGHT-DEPENDENT SHORT HYPOCOTYLS 6 IPR006936 (ALOG domain) Length=238
SOL Genomics	Niben	*Nicotiana benthamiana*	**Niben101Scf02049g00002.1**		72.87	6,00E-66	203	sp|Q9S7R3|LSH10_ARATH ***- Protein LIGHT-DEPENDENT SHORT HYPOCOTYLS 10 IPR006936 (ALOG domain) Length=196
SOL Genomics	Niben	*Nicotiana benthamiana*	**Niben101Scf04036g05001.1**		70.99	3,00E-65	200	sp|Q9S7R3|LSH10_ARATH ***- Protein LIGHT-DEPENDENT SHORT HYPOCOTYLS 10 IPR006936 (ALOG domain) Length=174

Database	Acr	Species	Subject ID	Gene name	Id%	E-value	Score	Description
SOL Genomics	Niben	*Nicotiana benthamiana*	**Niben101Scf04193g06012.1**		70.33	2,00E-27	101	sp|O82268|LSH3_ARATH *-*- Protein LIGHT-DEPENDENT SHORT HYPOCOTYLS 3 IPR006936 (ALOG domain) Length=97
SOL Genomics	Nisyl	*Nicotiana sylvestris*	**mRNA_41337_cds**		77.78	1,00E-70	216	mRNA_41337 gene_22472|id=AT5G28490.1:evalue=4e-72:annot='Protein of unknown function (DUF640)';id=Solyc04g009980.2.1:evalue=1e-91:annot='Light-dependent short hypocotyls 1'Length=603
SOL Genomics	Nisyl	*Nicotiana sylvestris*	**mRNA_16208_cds**		83.85	1,00E-68	209	mRNA_16208 gene_9494|id=AT2G31160.1:evalue=1e-84:annot='Protein of unknown function (DUF640)';id=Solyc05g055020.2.1:evalue=5e-81:annot='Light-dependent short hypocotyls 1'Length=552
SOL Genomics	Nisyl	*Nicotiana sylvestris*	**mRNA_16209_cds**		83.85	2,00E-68	210	mRNA_16209 gene_9494|id=AT2G31160.1:evalue=2e-84:annot='Protein of unknown function (DUF640)';id=Solyc05g055020.2.1:evalue=2e-80:annot='Light-dependent short hypocotyls 1'Length=600
SOL Genomics	Nisyl	*Nicotiana sylvestris*	**mRNA_11822_cds**		73.94	1,00E-67	209	mRNA_11822 gene_7212|id=AT1G0709

Database	Acr	Species	Subject ID	Gene name	Id%	E-value	Score	Description
								0.1:evalue=4e-94:annot='Protein of unknown function (DUF640)';id=Solyc06g082210.1.1:evalue=6e-102:annot='Light-dependent short hypocotyls 1'Length=711
SOL Genomics	Nisyl	*Nicotiana sylvestris*	**mRNA_70834_cds**		82.40	2,00E-64	199	mRNA_70834 gene_37372|id=AT2G31160.1:evalue=1e-79:annot='Protein of unknown function (DUF640)';id=Solyc05g055020.2.1:evalue=3e-81:annot='Light-dependent short hypocotyls 1'Length=573
SOL Genomics	Nisyl	*Nicotiana sylvestris*	**mRNA_8976_cds**		64.71	4,00E-60	187	mRNA_8976 gene_5636|id=AT2G42610.2:evalue=1e-84:annot='Protein of unknown function (DUF640)';id=Solyc10g008000.1.1:evalue=6e-101:annot='Light-dependent short hypocotyls 1'Length=531
SOL Genomics	Nisyl	*Nicotiana sylvestris*	**mRNA_21822_cds**		75.97	2,00E-59	187	mRNA_21822 gene_12430|id=AT2G42610.2:evalue=2e-76:annot='Protein of unknown function (DUF640)';id=Solyc12g014260.1.1:evalue=1e-101:annot='Light-dependent short hypocotyls 1'Length=591

**Database**	**Acr**	**Species**	**Subject ID**	**Gene name**	**Id%**	**E-value**	**Score**	**Description**
SOL Genomics	Nisyl	*Nicotiana sylvestris*	**mRNA_21821_cds**		75.97	2,00E-59	187	mRNA_21821 gene_12430|id=AT2G42610.2:evalue=2e-76:annot='Protein of unknown function (DUF640)';id=Solyc12g014260.1.1:evalue=1e-101:annot='Light-dependent short hypocotyls 1'Length=591
SOL Genomics	Nisyl	*Nicotiana sylvestris*	**mRNA_8273_cds**		74.42	3,00E-59	186	mRNA_8273 gene_5244|id=AT2G42610.2:evalue=2e-87:annot='Protein of unknown function (DUF640)';id=Solyc10g008000.1.1:evalue=1e-93:annot='Light-dependent short hypocotyls 1'Length=534
SOL Genomics	Nisyl	*Nicotiana sylvestris*	**mRNA_77587_cds**		59.12	7,00E-59	186	mRNA_77587 gene_40990|id=AT2G42610.2:evalue=9e-82:annot='Protein of unknown function (DUF640)';id=Solyc02g076820.2.1:evalue=7e-118:annot='Light-dependent short hypocotyls 1'Length=681
SOL Genomics	Nisyl	*Nicotiana sylvestris*	**mRNA_73157_cds**		74.19	1,00E-55	177	mRNA_73157 gene_38611|id=AT2G42610.2:evalue=2e-81:annot='Protein of unknown function (DUF640)';id=Solyc12g014260.1.1:evalue=8e-98:annot='Light-

Database	Acr	Species	Subject ID	Gene name	Id%	E-value	Score	Description
								dependent short hypocotyls 1'Length=621
SOL Genomics	Nisyl	*Nicotiana sylvestris*	**mRNA_73156_cds**		74.19	1,00E-55	177	mRNA_73156 gene_38611|id=AT2G42610.2:evalue=2e-81:annot='Protein of unknown function (DUF640)';id=Solyc12g014260.1.1:evalue=8e-98:annot='Light-dependent short hypocotyls 1'Length=621
SOL Genomics	Nitab	*Nicotiana tabacum*	**mRNA_132525**		74.43	3,00E-89	263	gene_62021|id=AT5G28490.1:evalue=1e-79:annot='Protein of unknown function (DUF640)';id=Solyc05g055020.2.1:evalue=3e-114:annot='Light-dependent short hypocotyls 1'Length=195
SOL Genomics	Nitab	*Nicotiana tabacum*	**mRNA_153991**		81.53	5,00E-88	260	gene_71963|id=AT5G28490.1:evalue=3e-78:annot='Protein of unknown function (DUF640)';id=Solyc05g055020.2.1:evalue=1e-113:annot='Light-dependent short hypocotyls 1'Length=197
SOL Genomics	Nitab	*Nicotiana tabacum*	**mRNA_105104**		74.36	4,00E-81	243	gene_49352|id=AT5G28490.1:evalue=4e-72:annot='Protein of unknown function (DUF640)';id=Solyc04g009980.2.1:evalue=1e-91:annot='Light-

Database	Acr	Species	Subject ID	Gene name	Id%	E-value	Score	Description
								dependent short hypocotyls 1'Length=200
SOL Genomics	Nitab	*Nicotiana tabacum*	**mRNA_60417**		78.87	7,00E-81	242	gene_28172|id=AT5G28490.1:evalue=3e-72:annot='Protein of unknown function (DUF640)';id=Solyc05g055020.2.1:evalue=1e-89:annot='Light-dependent short hypocotyls 1'Length=200
SOL Genomics	Nitab	*Nicotiana tabacum*	**mRNA_149085**		83.85	4,00E-69	211	gene_69799|id=AT2G31160.1:evalue=1e-84:annot='Protein of unknown function (DUF640)';id=Solyc05g055020.2.1:evalue=5e-81:annot='Light-dependent short hypocotyls 1'Length=183
SOL Genomics	Nitab	*Nicotiana tabacum*	**mRNA_131352**		74.42	5,00E-69	211	gene_61463|id=AT2G42610.2:evalue=2e-87:annot='Protein of unknown function (DUF640)';id=Solyc10g008000.1.1:evalue=1e-93:annot='Light-dependent short hypocotyls 1'Length=177
SOL Genomics	Nitab	*Nicotiana tabacum*	**mRNA_92749**		59.12	7,00E-69	212	gene_43125|id=AT2G42610.2:evalue=9e-82:annot='Protein of unknown function (DUF640)';id=Solyc02g076820.2.1:evalue=7e-118:annot='Light-dependent short hypocotyls 1'Length=226

Database	Acr	Species	Subject ID	Gene name	Id%	E-value	Score	Description
SOL Genomics	Nitab	*Nicotiana tabacum*	**mRNA_126695**		75.97	9,00E-69	211	gene_59274|id=AT2G42610.2:evalue=2e-76:annot='Protein of unknown function (DUF640)';id=Solyc12g014260.1.1:evalue=1e-101:annot='Light-dependent short hypocotyls 1'Length=196
SOL Genomics	Nitab	*Nicotiana tabacum*	**mRNA_149086**		83.85	9,00E-69	211	gene_69799|id=AT2G31160.1:evalue=9e-85:annot='Protein of unknown function (DUF640)';id=Solyc05g055020.2.1:evalue=6e-81:annot='Light-dependent short hypocotyls 1'Length=184
SOL Genomics	Nitab	*Nicotiana tabacum*	**mRNA_106213**		74.42	3,00E-68	209	gene_49855|id=AT2G42610.2:evalue=4e-87:annot='Protein of unknown function (DUF640)';id=Solyc10g008000.1.1:evalue=3e-93:annot='Light-dependent short hypocotyls 1'Length=177
SOL Genomics	Nitab	*Nicotiana tabacum*	**mRNA_186486**		73.33	2,00E-67	207	gene_86891|id=AT2G42610.2:evalue=2e-82:annot='Protein of unknown function (DUF640)';id=Solyc10g007310.1.1:evalue=2e-99:annot='Light-dependent short hypocotyls 1'Length=198
SOL Genomics	Nitab	*Nicotiana tabacum*	**mRNA_79929**		73.94	6,00E-67	208	gene_37151|id=AT1G07090.1:evalue=4e-

Database	Acr	Species	Subject ID	Gene name	Id%	E-value	Score	Description
								94:annot='Protein of unknown function (DUF640)';id=Solyc06g082210.1.1:evalue=6e-102:annot='Light-dependent short hypocotyls 1'Length=236
SOL Genomics	Nitab	*Nicotiana tabacum*	**mRNA_161164**		73.94	2,00E-66	207	gene_75261|id=AT1G07090.1:evalue=4e-93:annot='Protein of unknown function (DUF640)';id=Solyc06g082210.1.1:evalue=2e-101:annot='Light-dependent short hypocotyls 1'Length=237
SOL Genomics	Nitab	*Nicotiana tabacum*	**mRNA_35041**		82.40	2,00E-64	200	gene_16421|id=AT2G31160.1:evalue=1e-79:annot='Protein of unknown function (DUF640)';id=Solyc05g055020.2.1:evalue=3e-81:annot='Light-dependent short hypocotyls 1'Length=190
SOL Genomics	Nitab	*Nicotiana tabacum*	**mRNA_109505**		82.40	3,00E-64	199	gene_51306|id=AT1G07090.1:evalue=2e-79:annot='Protein of unknown function (DUF640)';id=Solyc06g083860.2.1:evalue=1e-81:annot='Light-dependent short hypocotyls 1'Length=196
SOL Genomics	Nitab	*Nicotiana tabacum*	**mRNA_32849**		77.52	5,00E-62	194	gene_15395|id=AT2G42610.2:evalue=4e-78:annot='Protein of unknown function

Database	Acr	Species	Subject ID	Gene name	Id%	E-value	Score	Description
								(DUF640)';id=Solyc12g014260.1.1:evalue=3e-99:annot='Light-dependent short hypocotyls 1'Length=194
SOL Genomics	Nitab	*Nicotiana tabacum*	**mRNA_32848**		77.52	5,00E-62	194	gene_15395|id=AT2G42610.2:evalue=4e-78:annot='Protein of unknown function (DUF640)';id=Solyc12g014260.1.1:evalue=3e-99:annot='Light-dependent short hypocotyls 1'Length=194
SOL Genomics	Nitab	*Nicotiana tabacum*	**mRNA_163645**		68.38	6,00E-56	177	gene_76391|id=AT2G42610.2:evalue=2e-65:annot='Protein of unknown function (DUF640)';id=Solyc07g062470.2.1:evalue=2e-77:annot='Light-dependent short hypocotyls 1'Length=141
SOL Genomics	Nitab	*Nicotiana tabacum*	mRNA_131353		74.42	5,00E-69	211	gene_61463|id=AT2G42610.2:evalue=2e-87:annot='Protein of unknown function (DUF640)';id=Solyc10g008000.1.1:evalue=1e-93:annot='Light-dependent short hypocotyls 1'Length=177
SOL Genomics	Nitab	*Nicotiana tabacum*	mRNA_126697		75.97	9,00E-69	211	gene_59274|id=AT2G42610.2:evalue=2e-76:annot='Protein of unknown function (DUF640)';id=Solyc12g014260.1.1:evalue=1e-

Database	Acr	Species	Subject ID	Gene name	Id%	E-value	Score	Description
								101:annot='Light-dependent short hypocotyls 1'Length=196
SOL Genomics	Nitab	*Nicotiana tabacum*	mRNA_126696		75.97	9,00E-69	211	gene_59274|id=AT2G42610.2:evalue=2e-76:annot='Protein of unknown function (DUF640)';id=Solyc12g014260.1.1:evalue=1e-101:annot='Light-dependent short hypocotyls 1'Length=196
SOL Genomics	Nitab	*Nicotiana tabacum*	mRNA_106214		74.42	3,00E-68	209	gene_49855|id=AT2G42610.2:evalue=4e-87:annot='Protein of unknown function (DUF640)';id=Solyc10g008000.1.1:evalue=3e-93:annot='Light-dependent short hypocotyls 1'Length=177
SOL Genomics	Nitab	*Nicotiana tabacum*	mRNA_8662		75.19	2,00E-67	207	gene_4079|id=AT2G42610.2:evalue=2e-82:annot='Protein of unknown function (DUF640)';id=Solyc02g076820.2.1:evalue=1e-114:annot='Light-dependent short hypocotyls 1'Length=185
SOL Genomics	Nitab	*Nicotiana tabacum*	mRNA_8661		75.19	2,00E-67	207	gene_4079|id=AT2G42610.2:evalue=2e-82:annot='Protein of unknown function (DUF640)';id=Solyc02g076820.2.1:evalue=1e-114:annot='Light-

Database	Acr	Species	Subject ID	Gene name	Id%	E-value	Score	Description
								dependent short hypocotyls 1'Length=185
SOL Genomics	Nitab	*Nicotiana tabacum*	mRNA_8660		75.19	2,00E-67	207	gene_4079|id=AT2G42610.2:evalue=2e-82:annot='Protein of unknown function (DUF640)';id=Solyc02g076820.2.1:evalue=1e-114:annot='Light-dependent short hypocotyls 1'Length=185
SOL Genomics	Nitab	*Nicotiana tabacum*	mRNA_186487		73.33	2,00E-67	207	gene_86891|id=AT2G42610.2:evalue=2e-82:annot='Protein of unknown function (DUF640)';id=Solyc10g007310.1.1:evalue=2e-99:annot='Light-dependent short hypocotyls 1'Length=198
SOL Genomics	Nitom	*Nicotiana tomentosiformis*	**mRNA_16036_cds**		74.43	1,00E-89	263	mRNA_16036 gene_10327|id=AT5G28490.1:evalue=1e-79:annot='Protein of unknown function (DUF640)';id=Solyc05g055020.2.1:evalue=2e-114:annot='Light-dependent short hypocotyls 1'Length=195
SOL Genomics	Nitom	*Nicotiana tomentosiformis*	**mRNA_33321_cds**		78.87	3,00E-81	242	mRNA_33321 gene_19203|id=AT5G28490.1:evalue=3e-72:annot='Protein of unknown function (DUF640)';id=Solyc05g055020.2.1:evalue=1e-89:annot='Light-

Database	Acr	Species	Subject ID	Gene name	Id%	E-value	Score	Description
								dependent short hypocotyls 1'Length=200
SOL Genomics	Nitom	*Nicotiana tomentosiformis*	**mRNA_52664_cds**		66.01	3,00E-72	218	mRNA_52664 gene_28981|id=AT2G42610.2:evalue=1e-86:annot='Protein of unknown function (DUF640)';id=Solyc10g008000.1.1:evalue=6e-104:annot='Light-dependent short hypocotyls 1'Length=176
SOL Genomics	Nitom	*Nicotiana tomentosiformis*	**mRNA_21168_cds**		74.42	1,00E-68	209	mRNA_21168 gene_12918|id=AT2G42610.2:evalue=4e-87:annot='Protein of unknown function (DUF640)';id=Solyc10g008000.1.1:evalue=3e-93:annot='Light-dependent short hypocotyls 1'Length=177
SOL Genomics	Nitom	*Nicotiana tomentosiformis*	**mRNA_253_cds**		75.19	6,00E-68	208	mRNA_253 gene_148|id=AT2G42610.2:evalue=1e-82:annot='Protein of unknown function (DUF640)';id=Solyc02g076820.2.1:evalue=3e-119:annot='Light-dependent short hypocotyls 1'Length=187
SOL Genomics	Nitom	*Nicotiana tomentosiformis*	**mRNA_46652_cds**		73.33	1,00E-67	207	mRNA_46652 gene_25975|id=AT2G42610.2:evalue=2e-82:annot='Protein of unknown function (DUF640)';id=Solyc10g0

Database	Acr	Species	Subject ID	Gene name	Id%	E-value	Score	Description
								07310.1.1:evalue=2e-99:annot='Light-dependent short hypocotyls 1'Length=198
SOL Genomics	Nitom	*Nicotiana tomentosiformis*	**mRNA_46651_cds**		73.33	1,00E-67	207	mRNA_46651 gene_25975|id=AT2G42610.2:evalue=2e-82:annot='Protein of unknown function (DUF640)';id=Solyc10g007310.1.1:evalue=2e-99:annot='Light-dependent short hypocotyls 1'Length=198
SOL Genomics	Nitom	*Nicotiana tomentosiformis*	**mRNA_46649_cds**		73.33	1,00E-67	207	mRNA_46649 gene_25975|id=AT2G42610.2:evalue=2e-82:annot='Protein of unknown function (DUF640)';id=Solyc10g007310.1.1:evalue=2e-99:annot='Light-dependent short hypocotyls 1'Length=198
SOL Genomics	Nitom	*Nicotiana tomentosiformis*	**mRNA_7291_cds**		73.94	2,00E-66	206	mRNA_7291 gene_4166|id=AT1G07090.1:evalue=2e-92:annot='Protein of unknown function (DUF640)';id=Solyc06g082210.1.1:evalue=6e-99:annot='Light-dependent short hypocotyls 1'Length=240
SOL Genomics	Nitom	*Nicotiana tomentosiformis*	**mRNA_46653_cds**		73.33	2,00E-65	203	mRNA_46653 gene_25975|id=AT2G42610.2:evalue=9e-80:annot='Protein of

Database	Acr	Species	Subject ID	Gene name	Id%	E-value	Score	Description
								unknown function (DUF640)';id=Solyc10g007310.1.1:evalue=2e-93:annot='Light-dependent short hypocotyls 1'Length=229
SOL Genomics	Nitom	*Nicotiana tomentosiformis*	**mRNA_75784_cds**		82.40	1,00E-64	200	mRNA_75784 gene_40962|id=AT1G07090.1:evalue=2e-79:annot='Protein of unknown function (DUF640)';id=Solyc06g083860.2.1:evalue=4e-80:annot='Light-dependent short hypocotyls 1'Length=203
SOL Genomics	Nitom	*Nicotiana tomentosiformis*	**mRNA_75782_cds**		82.40	2,00E-64	199	mRNA_75782 gene_40962|id=AT1G07090.1:evalue=2e-79:annot='Protein of unknown function (DUF640)';id=Solyc06g083860.2.1:evalue=3e-80:annot='Light-dependent short hypocotyls 1'Length=198
SOL Genomics	Nitom	*Nicotiana tomentosiformis*	**mRNA_75783_cds**		73.57	3,00E-61	191	mRNA_75783 gene_40962|id=AT1G07090.1:evalue=5e-76:annot='Protein of unknown function (DUF640)';id=Solyc02g069510.1.1:evalue=1e-76:annot='Light-dependent short hypocotyls 1'Length=213

**Database**	**Acr**	**Species**	**Subject ID**	**Gene name**	**Id%**	**E-value**	**Score**	**Description**
SOL Genomics	Nitom	*Nicotiana tomentosiformis*	**mRNA_46650_cds**		75.00	3,00E-58	183	mRNA_46650 gene_25975|id=AT2G42610.2:evalue=9e-72:annot='Protein of unknown function (DUF640)';id=Solyc12g014260.1.1:evalue=3e-83:annot='Light-dependent short hypocotyls 1'Length=198
Marpol	Nimir	*Nitella mirabilis*	**gb|GBST01051269.1**		68.33	7.34 × 10-55	248	TSA: Nitella mirabilis comp111411_c18_seq1 transcribed RNA sequence
Phytozome	Nycol	*Nymphaea colorata*	**Nycol.E00852.1.p**		87	1.43e-84	248.44	(1 of 2) PTHR31165:SF6 - PROTEIN LIGHT-DEPENDENT SHORT HYPOCOTYLS 6
Phytozome	Nycol	*Nymphaea colorata*	**Nycol.J01334.1.p**		88	5.33e-77	229.565	(1 of 2) PTHR31165:SF6 - PROTEIN LIGHT-DEPENDENT SHORT HYPOCOTYLS 6
Phytozome	Nycol	*Nymphaea colorata*	**Nycol.D01824.1.p**		83	1.28e-73	222.631	(1 of 2) PTHR31165:SF13 - PROTEIN LIGHT-DEPENDENT SHORT HYPOCOTYLS 1-RELATED
Phytozome	Nycol	*Nymphaea colorata*	**Nycol.N00286.1.p**		81	1.92e-71	217.624	(1 of 5) PF04852 - Protein of unknown function (DUF640) (DUF640)
Phytozome	Nycol	*Nymphaea colorata*	**Nycol.L00670.1.p**		82	1.04e-70	214.542	(1 of 2) PTHR31165//PTHR31165:SF13 - FAMILY NOT NAMED // PROTEIN LIGHT-DEPENDENT SHORT HYPOCOTYLS

Database	Acr	Species	Subject ID	Gene name	Id%	E-value	Score	Description
Phytozome	Oleur	*Olea europaea*	**Oeu064668.1**		83	2.26e-78	233.802	(1 of 3) PTHR31165//PTHR31165:SF6 - FAMILY NOT NAMED // PROTEIN LIGHT-DEPENDENT SHORT HYPOCOTYLS 6
Phytozome	Oleur	*Olea europaea*	**Oeu052697.1**		81	2.27e-76	229.18	(1 of 15) PF04852 - Protein of unknown function (DUF640) (DUF640)
Phytozome	Oleur	*Olea europaea*	**Oeu005347.2**		73	3.34e-76	228.024	(1 of 3) PTHR31165//PTHR31165:SF6 - FAMILY NOT NAMED // PROTEIN LIGHT-DEPENDENT SHORT HYPOCOTYLS 6
Phytozome	Oleur	*Olea europaea*	**Oeu053354.1**		85	6.90e-76	228.024	(1 of 3) PTHR31165//PTHR31165:SF6 - FAMILY NOT NAMED // PROTEIN LIGHT-DEPENDENT SHORT HYPOCOTYLS 6
Phytozome	Oleur	*Olea europaea*	**Oeu059153.1**		71	5.88e-74	221.476	(1 of 4) PTHR31165//PTHR31165:SF13 - FAMILY NOT NAMED // PROTEIN LIGHT-DEPENDENT SHORT HYPOCOTYLS 1-RELATED
Phytozome	Oleur	*Olea europaea*	**Oeu061356.1**		75	8.47e-73	218.394	(1 of 2) PTHR31165//PTHR31165:SF9 - FAMILY NOT NAMED // PROTEIN LIGHT-DEPENDENT

Database	Acr	Species	Subject ID	Gene name	Id%	E-value	Score	Description
								SHORT HYPOCOTYLS 7-RELATED
Phytozome	Oleur	*Olea europaea*	**Oeu031033.1**		74	1.38e-70	213.001	(1 of 2) PTHR31165//PTHR31165:SF9 - FAMILY NOT NAMED // PROTEIN LIGHT-DEPENDENT SHORT HYPOCOTYLS 7-RELATED
Phytozome	Oleur	*Olea europaea*	**Oeu039564.2**		75	1.71e-70	213.001	(1 of 4) PTHR31165//PTHR31165:SF13 - FAMILY NOT NAMED // PROTEIN LIGHT-DEPENDENT SHORT HYPOCOTYLS 1-RELATED
Phytozome	Oleur	*Olea europaea*	**Oeu055235.1**		79	2.30e-70	212.616	(1 of 4) PTHR31165//PTHR31165:SF13 - FAMILY NOT NAMED // PROTEIN LIGHT-DEPENDENT SHORT HYPOCOTYLS 1-RELATED
Phytozome	Oleur	*Olea europaea*	**Oeu053229.2**		72	7.52e-70	210.69	(1 of 2) PTHR31165//PTHR31165:SF10 - FAMILY NOT NAMED // PROTEIN LIGHT-DEPENDENT SHORT HYPOCOTYLS 10-RELATED
Phytozome	Oleur	*Olea europaea*	**Oeu001083.1**		73	1.65e-69	210.305	(1 of 15) PF04852 - Protein of unknown function (DUF640) (DUF640)
Phytozome	Oleur	*Olea europaea*	**Oeu034633.1**		71	6.92e-69	209.149	(1 of 15) PF04852 - Protein of unknown function (DUF640) (DUF640)

Database	Acr	Species	Subject ID	Gene name	Id%	E-value	Score	Description
Phytozome	Oleur	*Olea europaea*	**Oeu056121.2**		75	8.10e-64	196.052	(1 of 4) PTHR31165//PTHR31165:SF13 - FAMILY NOT NAMED // PROTEIN LIGHT-DEPENDENT SHORT HYPOCOTYLS 1-RELATED
Phytozome	Oleur	*Olea europaea*	**Oeu058992.1**		73	5.18e-62	191.045	(1 of 2) PTHR31165//PTHR31165:SF10 - FAMILY NOT NAMED // PROTEIN LIGHT-DEPENDENT SHORT HYPOCOTYLS 10-RELATED
Phytozome	Oleur	*Olea europaea*	Oeu025858.1		50	1.21e-34	120.168	(1 of 15) PF04852 - Protein of unknown function (DUF640) (DUF640)
Phytozome	Oleur	*Olea europaea*	Oeu016159.1		27	1.19e-2	365.798	(1 of 2) K02184 - formin 2 (FMN2)
Phytozome	Orsat	*Oryza sativa*	**LOC_Os05g28040.1**	OsG1L9	81.7	2.9E-68	213.8	DUF640 domain containing protein, putative, expressed
Phytozome	Orsat	*Oryza sativa*	**LOC_Os01g61310.1**	OsG1L7	80.2	4.6E-68	210.7	DUF640 domain containing protein, putative, expressed
Phytozome	Orsat	*Oryza sativa*	**LOC_Os02g41460.1**	OsG1L3	80.9	2.6E-67	208.8	DUF640 domain containing protein, putative, expressed
Phytozome	Orsat	*Oryza sativa*	**LOC_Os02g56610.1**	OsG1L6 (TH1/BH1/AFD1)	82.4	6.6E-67	209.1	DUF640 domain containing protein, putative, expressed
Phytozome	Orsat	*Oryza sativa*	**LOC_Os02g07030.1**	OsG1L1	71	2.6E-66	208.4	DUF640 domain containing protein, putative, expressed
Phytozome	Orsat	*Oryza sativa*	**LOC_Os06g46030.1**	OsG1L2	81.3	3.3E-66	208.4	DUF640 domain containing protein, putative, expressed

Database	Acr	Species	Subject ID	Gene name	Id%	E-value	Score	Description
Phytozome	Orsat	*Oryza sativa*	**LOC_Os10g33780.1**	OsG1L5 (TAWAWA1)	85.7	1.4E-65	204.1	DUF640 domain containing protein, putative, expressed
Phytozome	Orsat	*Oryza sativa*	**LOC_Os04g43580.1**	OsG1L4	82.4	2.2E-65	203.8	DUF640 domain containing protein, putative, expressed
Phytozome	Orsat	*Oryza sativa*	**LOC_Os05g39500.1**	OsG1L8	77.9	3.7E-65	204.1	DUF640 domain containing protein, putative, expressed
Phytozome	Orsat	*Oryza sativa*	**LOC_Os07g04670.1**	OsG1	55.5	1.2E-41	144.4	DUF640 domain containing protein, putative, expressed
Phytozome	Orsat	*Oryza sativa*	LOC_Os08g14970.1	OsG1L13	78	8.1E-26	100.5	DUF640 domain containing protein, putative, expressed
Phytozome	Orsat	*Oryza sativa*	LOC_Os01g54180.1	OsG1L10	72.1	2.8E-25	100.5	DUF640 domain containing protein, putative, expressed
Phytozome	Orsat	*Oryza sativa*	LOC_Os05g27120.1	OsG1L11	67.2	1.2E-21	89.4	DUF640 domain containing protein, putative, expressed
Phytozome	Orsat	*Oryza sativa*	LOC_Os08g09660.1	OsG1L12	77.8	8,00E-18	77.0	DUF640 domain containing protein, putative, expressed
Phytozome	Pavag	*Paspalum vaginatum*	**Pavag09G098800.1.p**		87	4.51e-73	223.787	(1 of 1) PTHR31165//PTHR31165:SF6 - FAMILY NOT NAMED // PROTEIN LIGHT-DEPENDENT SHORT HYPOCOTYLS 6
Phytozome	Pavag	*Paspalum vaginatum*	**Pavag04G202000.1.p**		81	2.31e-72	218.394	(1 of 10) PF04852 - Protein of unknown function (DUF640) (DUF640)
Phytozome	Pavag	*Paspalum vaginatum*	**Pavag03G323400.1.p**		83	4.86e-71	215.312	(1 of 10) PF04852 - Protein of unknown

Database	Acr	Species	Subject ID	Gene name	Id%	E-value	Score	Description
								function (DUF640) (DUF640)
Phytozome	Pavag	*Paspalum vaginatum*	**Pavag09G174100.1.p**		85	2.60e-70	214.542	(1 of 10) PF04852 - Protein of unknown function (DUF640) (DUF640)
Phytozome	Pavag	*Paspalum vaginatum*	**Pavag06G159000.1.p**		78	7.41e-70	211.846	(1 of 10) PF04852 - Protein of unknown function (DUF640) (DUF640)
Phytozome	Pavag	*Paspalum vaginatum*	**Pavag04G313700.1.p**		81	2.85e-68	209.92	(1 of 10) PF04852 - Protein of unknown function (DUF640) (DUF640)
Phytozome	Pavag	*Paspalum vaginatum*	**Pavag01G204000.1.p**		79	4.41e-68	207.994	(1 of 10) PF04852 - Protein of unknown function (DUF640) (DUF640)
Phytozome	Pavag	*Paspalum vaginatum*	**Pavag10G224900.1.p**		80	5.75e-67	207.223	(1 of 10) PF04852 - Protein of unknown function (DUF640) (DUF640)
Phytozome	Pavag	*Paspalum vaginatum*	**Pavag04G049000.1.p**		77	1.41e-64	200.29	(1 of 10) PF04852 - Protein of unknown function (DUF640) (DUF640)
Phytozome	Pavag	*Paspalum vaginatum*	**Pavag02G030200.1.p**		59	2.89e-51	165.622	(1 of 10) PF04852 - Protein of unknown function (DUF640) (DUF640)
SOL Genomics	Peaxi	*Petunia axillaris*	**Peaxi162Scf00015g03113.1**	PaLSH1	76.61	2,00E-80	239	Protein LIGHT-DEPENDENT SHORT HYPOCOTYLS 1Length=194
SOL Genomics	Peaxi	*Petunia axillaris*	**Peaxi162Scf00390g00086.1**	PaLSH5	66.88	1,00E-76	231	Protein of unknown function (DUF640)Length=228

Database	Acr	Species	Subject ID	Gene name	Id%	E-value	Score	Description
SOL Genomics	Peaxi	*Petunia axillaris*	**Peaxi162Scf01265g00022.1**	PaLSH4	86.92	2,00E-71	216	Protein of unknown function (DUF640)Length=188
SOL Genomics	Peaxi	*Petunia axillaris*	**Peaxi162Scf00692g00062.1**	PaLSH7a	74.24	7,00E-70	212	Protein LIGHT-DEPENDENT SHORT HYPOCOTYLS 10Length=193
SOL Genomics	Peaxi	*Petunia axillaris*	**Peaxi162Scf00469g00032.1**	PaLSH10b	74.42	8,00E-70	212	Protein LIGHT-DEPENDENT SHORT HYPOCOTYLS 10Length=177
SOL Genomics	Peaxi	*Petunia axillaris*	**Peaxi162Scf00666g00042.1**	PaLSH10a	68.92	1,00E-69	211	Protein LIGHT-DEPENDENT SHORT HYPOCOTYLS 10Length=179
SOL Genomics	Peaxi	*Petunia axillaris*	**Peaxi162Scf00134g00151.1**	PaLSH3b	84.62	2,00E-69	211	Protein of unknown function (DUF640)Length=191
SOL Genomics	Peaxi	*Petunia axillaris*	**Peaxi162Scf01106g00015.1**	PaLSH10c	74.07	9,00E-69	209	Protein LIGHT-DEPENDENT SHORT HYPOCOTYLS 10Length=171
SOL Genomics	Peaxi	*Petunia axillaris*	**Peaxi162Scf00013g01121.1**	PaLSH3a	79.58	1,00E-68	209	Protein of unknown function (DUF640)Length=198
SOL Genomics	Peaxi	*Petunia axillaris*	**Peaxi162Scf00195g00310.1**	PaLSH2	75.37	3,00E-66	202	Protein of unknown function (DUF640)Length=172
SOL Genomics	Peaxi	*Petunia axillaris*	**Peaxi162Scf00111g01424.1**	PaLSH7b	72.66	1,00E-64	198	Protein LIGHT-DEPENDENT SHORT HYPOCOTYLS 10Length=184
NCBI	Pehyb	*Petunia hybrida*	**QCY72054.1**		76.61%	4,00E-80	239	light-dependent short hypocotyls 1 [Petunia x hybrida]

Database	Acr	Species	Subject ID	Gene name	Id%	E-value	Score	Description
NCBI	Pehyb	*Petunia hybrida*	**QCY72059.1**		73.94%	5,00E-77	233	light-dependent short hypocotyls 5 [Petunia x hybrida]
NCBI	Pehyb	*Petunia hybrida*	**QCY72058.1**		86.92%	4,00E-71	216	light-dependent short hypocotyls 4 [Petunia x hybrida]
NCBI	Pehyb	*Petunia hybrida*	**QCY72063.1**		74.42%	1,00E-69	212	light-dependent short hypocotyls 10b [Petunia x hybrida]
NCBI	Pehyb	*Petunia hybrida*	**QCY72062.1**		68.92%	1,00E-69	212	light-dependent short hypocotyls 10a [Petunia x hybrida]
NCBI	Pehyb	*Petunia hybrida*	**QCY72060.1**		74.24%	2,00E-69	212	light-dependent short hypocotyls 7a [Petunia x hybrida]
NCBI	Pehyb	*Petunia hybrida*	**QCY72057.1**		84.62%	6,00E-69	211	light-dependent short hypocotyls 3b [Petunia x hybrida]
NCBI	Pehyb	*Petunia hybrida*	**QCY72064.1**		74.07%	7,00E-69	210	light-dependent short hypocotyls 10c [Petunia x hybrida]
NCBI	Pehyb	*Petunia hybrida*	**QCY72056.1**		79.58%	5,00E-71	209	light-dependent short hypocotyls 3a [Petunia x hybrida]
NCBI	Pehyb	*Petunia hybrida*	**QCY72055.1**		75.37%	1,00E-68	202	light-dependent short hypocotyls 2 [Petunia x hybrida]
NCBI	Pehyb	*Petunia hybrida*	**QCY72061.1**		72.66%	2,00E-67	199	light-dependent short hypocotyls 7b [Petunia x hybrida]
SOL Genomics	Peinf	*Petunia inflata*	**Peinf101Scf00056g09007.1**	PiLSH1	76.61	3,00E-80	239	Protein LIGHT-DEPENDENT SHORT HYPOCOTYLS 1Length=196
SOL Genomics	Peinf	*Petunia inflata*	**Peinf101Scf00672g08001.1**	PiLSH4	86.92	2,00E-71	216	Protein of unknown function (DUF640)Length=190

Database	Acr	Species	Subject ID	Gene name	Id%	E-value	Score	Description
SOL Genomics	Peinf	*Petunia inflata*	**Peinf101Scf00132g13001.1**	PiLSH7a	68.97	4,00E-70	213	Protein LIGHT-DEPENDENT SHORT HYPOCOTYLS 10Length=194
SOL Genomics	Peinf	*Petunia inflata*	**Peinf101Scf01267g00004.1**	PiLSH10b	74.42	2,00E-69	211	Protein LIGHT-DEPENDENT SHORT HYPOCOTYLS 10Length=177
SOL Genomics	Peinf	*Petunia inflata*	**Peinf101Scf00879g22002.1**	PiLSH3b	84.62	2,00E-69	211	Protein of unknown function (DUF640)Length=191
SOL Genomics	Peinf	*Petunia inflata*	**Peinf101Scf00305g01002.1**	PiLSH0a	68.92	3,00E-69	210	Protein LIGHT-DEPENDENT SHORT HYPOCOTYLS 10Length=179
SOL Genomics	Peinf	*Petunia inflata*	**Peinf101Scf18795g00004.1**	PiLSH3a	76.55	5,00E-69	210	Protein of unknown function (DUF640)Length=195
SOL Genomics	Peinf	*Petunia inflata*	**Peinf101Scf00543g17002.1**	PiLSH10c	74.07	9,00E-69	209	Protein LIGHT-DEPENDENT SHORT HYPOCOTYLS 10Length=178
SOL Genomics	Peinf	*Petunia inflata*	**Peinf101Scf02200g00008.1**	PiLSH5	68.12	3,00E-67	205	Protein of unknown function (DUF640)Length=161
SOL Genomics	Peinf	*Petunia inflata*	**Peinf101Scf00793g07002.1**	PiLSH2	70.95	1,00E-66	203	Protein of unknown function (DUF640)Length=160
SOL Genomics	Peinf	*Petunia inflata*	**Peinf101Scf00665g03003.1**	PiLSH7b	72.66	9,00E-65	199	Protein LIGHT-DEPENDENT SHORT HYPOCOTYLS 10Length=184
SOL Genomics	Peinf	*Petunia inflata*	**Peinf101Scf08825g00007.1**		77.42	3,00E-51	162	Protein of unknown function (DUF640)Length=100

Database	Acr	Species	Subject ID	Gene name	Id%	E-value	Score	Description
SOL Genomics	Peinf	*Petunia inflata*	**Peinf101Scf08825g00006.1**		77.42	5,00E-51	161	Protein of unknown function (DUF640)Length=94
Phytozome	Phvul	*Phaseolus vulgaris*	**Phvul.005G066200.1.p**		78.4	1.2E-61	193.0	(1 of 2) PTHR31165:SF9 - PROTEIN LIGHT-DEPENDENT SHORT HYPOCOTYLS 7-RELATED
Phytozome	Phvul	*Phaseolus vulgaris*	**Phvul.005G095100.1.p**		73.6	3.1E-58	184.1	(1 of 2) PTHR31165:SF10 - PROTEIN LIGHT-DEPENDENT SHORT HYPOCOTYLS 10-RELATED
Phytozome	Phvul	*Phaseolus vulgaris*	**Phvul.011G162700.1.p**		75.2	2,00E-58	185.3	(1 of 2) PTHR31165:SF9 - PROTEIN LIGHT-DEPENDENT SHORT HYPOCOTYLS 7-RELATED
Phytozome	Phvul	*Phaseolus vulgaris*	**Phvul.011G130300.1.p**		73.1	1.4E-58	185.3	(1 of 2) PTHR31165:SF10 - PROTEIN LIGHT-DEPENDENT SHORT HYPOCOTYLS 10-RELATED
Phytozome	Phvul	*Phaseolus vulgaris*	**Phvul.L001960.1.p**		72.8	2.6E-75	228.0	(1 of 16) PF04852 - Protein of unknown function (DUF640) (DUF640)
Phytozome	Phvul	*Phaseolus vulgaris*	**Phvul.L001675.1.p**		72.8	2.6E-75	228.0	(1 of 16) PF04852 - Protein of unknown function (DUF640)
Phytozome	Phvul	*Phaseolus vulgaris*	**Phvul.008G258200.1.p**		78.6	4,00E-63	198.4	(1 of 2) PTHR31165:SF6 - PROTEIN LIGHT-DEPENDENT SHORT HYPOCOTYLS 6
Phytozome	Phvul	*Phaseolus vulgaris*	**Phvul.007G108200.1.p**		87	1.2E-71	219.2	(1 of 2) PTHR31165:SF13 -

Database	Acr	Species	Subject ID	Gene name	Id%	E-value	Score	Description
								PROTEIN LIGHT-DEPENDENT SHORT HYPOCOTYLS 1-RELATED
Phytozome	Phvul	*Phaseolus vulgaris*	**Phvul.007G274100.1.p**		86.9	4.1E-70	215.7	(1 of 2) PTHR31165:SF13 - PROTEIN LIGHT-DEPENDENT SHORT HYPOCOTYLS 1-RELATED
Phytozome	Phvul	*Phaseolus vulgaris*	**Phvul.007G192200.1.p**		85.2	7.9E-67	207.6	(1 of 16) PF04852 - Protein of unknown function (DUF640)
Phytozome	Phvul	*Phaseolus vulgaris*	**Phvul.001G161400.1.p**		86.4	3.1E-66	205.7	(1 of 16) PF04852 - Protein of unknown function (DUF640)
Phytozome	Phvul	*Phaseolus vulgaris*	**Phvul.006G105500.1.p**		84.2	2.5E-70	215.3	(1 of 16) PF04852 - Protein of unknown function (DUF640)
Phytozome	Phvul	*Phaseolus vulgaris*	**Phvul.006G176100.1.p**		71.6	4.7E-58	184.1	(1 of 16) PF04852 - Protein of unknown function (DUF640)
Phytozome	Phvul	*Phaseolus vulgaris*	**Phvul.006G072500.1.p**		76	4.2E-58	185.3	(1 of 2) PTHR31165:SF6 - PROTEIN LIGHT-DEPENDENT SHORT HYPOCOTYLS 6
Phytozome	Phvul	*Phaseolus vulgaris*	**Phvul.002G238600.1.p**		70.7	9.8E-57	180.6	(1 of 16) PF04852 - Protein of unknown function (DUF640)
Phytozome	Phvul	*Phaseolus vulgaris*	**Phvul.002G202200.1.p**		78.4	1,00E-67	210.3	(1 of 16) PF04852 - Protein of unknown function (DUF640)
Phytozome	Phpat	*Physcomitrella patens*	**Pp3c8_6310V3.1.p**		71.4	8,00E-66	208.4	(1 of 4) PF04852 - Protein of unknown function (DUF640) (DUF640)
Phytozome	Phpat	*Physcomitrella patens*	**Pp3c20_17990V3.1.p**		80.2	1.4E-65	208.0	(1 of 4) PF04852 - Protein of unknown function (DUF640) (DUF640)

**Database**	**Acr**	**Species**	**Subject ID**	**Gene name**	**Id%**	**E-value**	**Score**	**Description**
Phytozome	Phpat	*Physcomitrella patens*	**Pp3c24_8490V3.1.p**		80.5	7.2E-65	206.1	(1 of 4) PF04852 - Protein of unknown function (DUF640) (DUF640)
Phytozome	Phpat	*Physcomitrella patens*	**Pp3c23_9660V3.1.p**		78.6	9.9E-64	203.0	(1 of 4) PF04852 - Protein of unknown function (DUF640) (DUF640)
congenie	Piabi	*Picea abiens*	**MA_12926**		50.24	4.54e-5	125.15	-
congenie	Piabi	*Picea abiens*	MA_211369		58.62	2.80e-7	81.71	-
congenie	Piabi	*Picea abiens*	MA_139833		58.48	9.34e-14	158.35	-
congenie	Pitae	*Pinus taeda*	**MA_12926g0010**		52.14	3.10e-12	145.04	-
Phytozome	Poami	*Portulaca amilis*	**FUN_007669-T1**		91	3.67e-83	246.514	(1 of 1) PTHR31165:SF6 - PROTEIN LIGHT-DEPENDENT SHORT HYPOCOTYLS 6
Phytozome	Poami	*Portulaca amilis*	**FUN_046698-T1**		73	8.81e-69	209.534	(1 of 1) PTHR31165//PTHR31165:SF9 - FAMILY NOT NAMED // PROTEIN LIGHT-DEPENDENT SHORT HYPOCOTYLS 7-RELATED
Phytozome	Poami	*Portulaca amilis*	**FUN_051281-T1**		78	8.52e-68	206.453	(1 of 4) PTHR31165:SF13 - PROTEIN LIGHT-DEPENDENT SHORT HYPOCOTYLS 1-RELATED
Phytozome	Poami	*Portulaca amilis*	**FUN_036794-T1**		68	1.10e-66	204.142	(1 of 2) PTHR31165//PTHR31165:SF10 - FAMILY NOT NAMED // PROTEIN LIGHT-DEPENDENT SHORT HYPOCOTYLS 10-RELATED
Phytozome	Poami	*Portulaca amilis*	**FUN_000203-T1**		66	1.53e-66	202.986	(1 of 4) IPR006936//IPR023109 -

Database	Acr	Species	Subject ID	Gene name	Id%	E-value	Score	Description
								ALOG domain // Integrase/recombinase, N-terminal
Phytozome	Poami	*Portulaca amilis*	**FUN_051274-T1**		76	4.11e-66	202.216	(1 of 4) PTHR31165:SF13 - PROTEIN LIGHT-DEPENDENT SHORT HYPOCOTYLS 1-RELATED
Phytozome	Poami	*Portulaca amilis*	**FUN_002707-T1**		76	4.52e-66	201.83	(1 of 4) PTHR31165//PTHR31165:SF13 - FAMILY NOT NAMED // PROTEIN LIGHT-DEPENDENT SHORT HYPOCOTYLS 1-RELATED
Phytozome	Poami	*Portulaca amilis*	**FUN_037129-T1**		75	2.27e-63	195.667	(1 of 2) PTHR31165//PTHR31165:SF10 - FAMILY NOT NAMED // PROTEIN LIGHT-DEPENDENT SHORT HYPOCOTYLS 10-RELATED
Phytozome	Poami	*Portulaca amilis*	FUN_044761-T1		67	1.33e-25	951.301	(1 of 4) PTHR31165:SF13 - PROTEIN LIGHT-DEPENDENT SHORT HYPOCOTYLS 1-RELATED
Phytozome	Prper	*Prunus persica*	**Prupe.8G222300.1.p**		84.6	1.4E-69	214.9	(1 of 2) PTHR31165:SF13 - PROTEIN LIGHT-DEPENDENT SHORT HYPOCOTYLS 1-RELATED
Phytozome	Prper	*Prunus persica*	**Prupe.6G349800.1.p**		72.2	1.3E-77	235.3	(1 of 8) PF04852 - Protein of unknown function (DUF640) (DUF640)

Database	Acr	Species	Subject ID	Gene name	Id%	E-value	Score	Description
Phytozome	Prper	*Prunus persica*	**Prupe.6G051600.1.p**		77.7	5.3E-65	203.8	(1 of 1) PTHR31165:SF6 - PROTEIN LIGHT-DEPENDENT SHORT HYPOCOTYLS 6
Phytozome	Prper	*Prunus persica*	**Prupe.4G101300.1.p**		79.1	1,00E-66	209.1	(1 of 8) PF04852 - Protein of unknown function (DUF640) (DUF640)
Phytozome	Prper	*Prunus persica*	**Prupe.4G061700.1.p**		71.2	7.9E-57	181.4	(1 of 8) PF04852 - Protein of unknown function (DUF640) (DUF640)
Phytozome	Prper	*Prunus persica*	**Prupe.4G165300.1.p**		75.2	1.4E-58	185.7	(1 of 1) PTHR31165:SF9 - PROTEIN LIGHT-DEPENDENT SHORT HYPOCOTYLS 7-RELATED
Phytozome	Prper	*Prunus persica*	**Prupe.3G118100.1.p**		75.2	4.3E-59	186.8	(1 of 1) PTHR31165:SF10 - PROTEIN LIGHT-DEPENDENT SHORT HYPOCOTYLS 10-RELATED
Phytozome	Prper	*Prunus persica*	**Prupe.1G040300.1.p**		80.7	2.3E-70	216.9	(1 of 2) PTHR31165:SF13 - PROTEIN LIGHT-DEPENDENT SHORT HYPOCOTYLS 1-RELATED
Phytozome	Semoe	*Selaginella moellendorffii*	**36560**		77.3	3.5E-70	213.4	(1 of 2) PF04852 - Protein of unknown function (DUF640) (DUF640)
Phytozome	Semoe	*Selaginella moellendorffii*	**68566**		79.4	3.7E-68	208.8	(1 of 2) PF04852 - Protein of unknown function (DUF640) (DUF640)
NCBI	Sochi	*Solanum chilense*	**TMW91528.1**		83.45%	8,00E-88	259	hypothetical protein EJD97_014241 [Solanum chilense]

Database	Acr	Species	Subject ID	Gene name	Id%	E-value	Score	Description
NCBI	Sochi	*Solanum chilense*	**TMX02388.1**		63.22%	1,00E-81	243	hypothetical protein EJD97_021684 [Solanum chilense]
NCBI	Sochi	*Solanum chilense*	**TMX05780.1**		74.82%	3,00E-75	226	hypothetical protein EJD97_001815 [Solanum chilense]
NCBI	Sochi	*Solanum chilense*	**TMW90767.1**		85.93%	2,00E-72	220	hypothetical protein EJD97_015288 [Solanum chilense]
NCBI	Sochi	*Solanum chilense*	**TMW81845.1**		85.38%	3,00E-70	214	hypothetical protein EJD97_007625 [Solanum chilense]
NCBI	Sochi	*Solanum chilense*	**TMW92288.1**		71.63%	2,00E-71	213	hypothetical protein EJD97_013234 [Solanum chilense]
NCBI	Sochi	*Solanum chilense*	**TMW96855.1**		67.55%	7,00E-69	211	hypothetical protein EJD97_006697 [Solanum chilense]
NCBI	Sochi	*Solanum chilense*	**TMW89251.1**		71.97%	4,00E-70	209	hypothetical protein EJD97_017450 [Solanum chilense]
NCBI	Sochi	*Solanum chilense*	**TMW82134.1**		74.62%	6,00E-70	210	hypothetical protein EJD97_006715 [Solanum chilense]
NCBI	Sochi	*Solanum chilense*	**TMW83025.1**		74.29%	4,00E-69	209	hypothetical protein EJD97_003233 [Solanum chilense]
NCBI	Sochi	*Solanum chilense*	**TMW88518.1**		85.60%	1,00E-68	207	hypothetical protein EJD97_018443 [Solanum chilense]
NCBI	Sochi	*Solanum chilense*	**TMW91335.1**		72.09%	2,00E-67	203	hypothetical protein EJD97_014468 [Solanum chilense]
NCBI	Socom	*Solanum commersonii*	**KAG5607765.1**		84.72%	1,00E-88	259	hypothetical protein H5410_029257 [Solanum commersonii]

Database	Acr	Species	Subject ID	Gene name	Id%	E-value	Score	Description
NCBI	Socom	*Solanum commersonii*	**KAG5608871.1**		62.50%	4,00E-82	242	hypothetical protein H5410_020152 [Solanum commersonii]
NCBI	Socom	*Solanum commersonii*	**KAG5588181.1**		76.69%	4,00E-77	229	hypothetical protein H5410_048615 [Solanum commersonii]
NCBI	Socom	*Solanum commersonii*	**KAG5621982.1**		74.47%	8,00E-77	228	hypothetical protein H5410_007200 [Solanum commersonii]
NCBI	Socom	*Solanum commersonii*	**KAG5587333.1**		81.69%	1,00E-71	214	hypothetical protein H5410_047767 [Solanum commersonii]
NCBI	Socom	*Solanum commersonii*	**KAG5597240.1**		75.19%	1,00E-70	211	hypothetical protein H5410_038472 [Solanum commersonii]
NCBI	Socom	*Solanum commersonii*	**KAG5569132.1**		75.38%	5,00E-70	211	hypothetical protein H5410_058898 [Solanum commersonii]
NCBI	Socom	*Solanum commersonii*	**KAG5622405.1**		74.62%	7,00E-70	211	hypothetical protein H5410_007623 [Solanum commersonii]
NCBI	Socom	*Solanum commersonii*	**KAG5579135.1**		71.21%	1,00E-69	210	hypothetical protein H5410_049762 [Solanum commersonii]
NCBI	Socom	*Solanum commersonii*	**KAG5579042.1**		76.80%	2,00E-69	210	hypothetical protein H5410_049669 [Solanum commersonii]
NCBI	Socom	*Solanum commersonii*	**KAG5603102.1**		74.29%	2,00E-68	208	hypothetical protein H5410_034472 [Solanum commersonii]
NCBI	Socom	*Solanum commersonii*	**KAG5603288.1**		84.80%	8,00E-68	206	hypothetical protein H5410_034658 [Solanum commersonii]
NCBI	Socom	*Solanum commersonii*	**KAG5597566.1**		71.32%	2,00E-66	202	hypothetical protein H5410_038798 [Solanum commersonii]

Database	Acr	Species	Subject ID	Gene name	Id%	E-value	Score	Description
SOL Genomics	Solyc	*Solanum lycopersicum*	**Solyc05g055020.4.1**	SolyLSH1a	83.45	2,00E-89	260	unnamed protein productLength=211
SOL Genomics	Solyc	*Solanum lycopersicum*	**Solyc09g025280.1.1**	SolyLSH3b	85.38	4,00E-72	215	unnamed protein productLength=171
SOL Genomics	Solyc	*Solanum lycopersicum*	**Solyc06g082210.1.1**	SolyLSH5	74.29	1,00E-69	211	unnamed protein productLength=222
SOL Genomics	Solyc	*Solanum lycopersicum*	**Solyc04g009980.4.1**	SolyLSH1b	62.36	2,00E-82	241	unnamed protein productLength=180
SOL Genomics	Solyc	*Solanum lycopersicum*	**Solyc06g083860.2.1**	SolyLSH3a	85.60	3,00E-68	207	unnamed protein productLength=218
SOL Genomics	Solyc	*Solanum lycopersicum*	**Solyc02g069510.1.1**	SolyLSH2	76.47	1,00E-76	227	unnamed protein productLength=181
SOL Genomics	Solyc	*Solanum lycopersicum*	**Solyc12g014260.1.1**	SolyLSH7c	76.15	9,00E-71	213	unnamed protein productLength=205
SOL Genomics	Solyc	*Solanum lycopersicum*	**Solyc07g150147.1.1**	SolyLSH10b	74.62	8,00E-71	213	unnamed protein productLength=196
SOL Genomics	Solyc	*Solanum lycopersicum*	**Solyc10g007310.1.1**	SolyLSH7a	74.62	7,00E-70	211	unnamed protein productLength=213
SOL Genomics	Solyc	*Solanum lycopersicum*	**Solyc10g008000.1.1**	SolyLSH10a	71.97	3,00E-70	211	unnamed protein productLength=178
SOL Genomics	Solyc	*Solanum lycopersicum*	**Solyc02g076820.4.1**	SolyLSH10c	75.19	9,00E-69	208	unnamed protein productLength=227
SOL Genomics	Solyc	*Solanum lycopersicum*	**Solyc07g062470.4.1**	SolyLSH7b	64.39	8,00E-61	187	unnamed protein productLength=193
SOL Genomics	Solyc	*Solanum lycopersicum*	Solyc09g090180.1.1	TMF	85.93	1,00E-73	220	unnamed protein productLength=208
SOL Genomics	Somel	*Solanum melongena*	**Sme2.5_02156.1_g00005.1**		76.47	4,00E-74	222	unnamed protein productLength=177
SOL Genomics	Somel	*Solanum melongena*	**Sme2.5_00323.1_g00010.1**		69.72	4,00E-70	213	unnamed protein productLength=178
SOL Genomics	Somel	*Solanum melongena*	**Sme2.5_18064.1_g00001.1**		87.20	2,00E-69	211	unnamed protein productLength=184
SOL Genomics	Somel	*Solanum melongena*	**Sme2.5_01584.1_g00001.1**		72.26	1,00E-68	209	unnamed protein productLength=175
SOL Genomics	Somel	*Solanum melongena*	**Sme2.5_07722.1_g00004.1**		74.42	2,00E-68	209	unnamed protein productLength=200

Database	Acr	Species	Subject ID	Gene name	Id%	E-value	Score	Description
SOL Genomics	Somel	*Solanum melongena*	**Sme2.5_02824.1_g00002.1**		76.61	1,00E-66	204	unnamed protein productLength=201
SOL Genomics	Somel	*Solanum melongena*	**Sme2.5_00295.1_g00002.1**		73.94	2,00E-66	205	unnamed protein productLength=236
SOL Genomics	Somel	*Solanum melongena*	**Sme2.5_06530.1_g00003.1**		69.12	2,00E-65	200	unnamed protein productLength=182
SOL Genomics	Somel	*Solanum melongena*	**Sme2.5_00014.1_g00031.1**		80.30	2,00E-64	198	unnamed protein productLength=187
SOL Genomics	Somel	*Solanum melongena*	**Sme2.5_02000.1_g00010.1**		74.00	1,00E-50	161	unnamed protein productLength=131
SOL Genomics	Somel	*Solanum melongena*	**Sme2.5_02418.1_g00008.1**		78.05	4,00E-42	140	unnamed protein productLength=169
SOL Genomics	Somel	*Solanum melongena*	Sme2.5_00396.1_g00005.1		77.78	4,00E-83	246	unnamed protein productLength=206
SOL Genomics	Sopen	*Solanum pennellii*	**Sopen05g033480**		84.14	3,00E-88	260	Protein of unknown function (DUF640)Length=211
SOL Genomics	Sopen	*Solanum pennellii*	**Sopen02g018650**		76.47	6,00E-75	225	Protein of unknown function (DUF640)Length=180
SOL Genomics	Sopen	*Solanum pennellii*	**Sopen09g033090**		85.71	5,00E-73	221	Protein of unknown function (DUF640)Length=206
SOL Genomics	Sopen	*Solanum pennellii*	**Sopen09g028240**		85.38	1,00E-70	214	Protein of unknown function (DUF640)Length=172
SOL Genomics	Sopen	*Solanum pennellii*	**Sopen12g006050**		76.92	2,00E-70	215	Protein of unknown function (DUF640)Length=203
SOL Genomics	Sopen	*Solanum pennellii*	**Sopen07g027140**		71.63	8,00E-70	212	Protein of unknown function (DUF640)Length=167
SOL Genomics	Sopen	*Solanum pennellii*	**Sopen10g003120**		69.39	6,00E-69	211	Protein of unknown function (DUF640)Length=211

Database	Acr	Species	Subject ID	Gene name	Id%	E-value	Score	Description
SOL Genomics	Sopen	*Solanum pennellii*	**Sopen06g033590**		74.29	4,00E-68	210	Protein of unknown function (DUF640)Length=226
SOL Genomics	Sopen	*Solanum pennellii*	**Sopen02g021720**		75.19	4,00E-68	209	Protein of unknown function (DUF640)Length=191
SOL Genomics	Sopen	*Solanum pennellii*	**Sopen06g035260**		85.60	3,00E-67	207	Protein of unknown function (DUF640)Length=216
SOL Genomics	Sopen	*Solanum pennellii*	**Sopen07g030710**		71.43	7,00E-66	202	Protein of unknown function (DUF640)Length=186
SOL Genomics	Sopen	*Solanum pennellii*	**Sopen10g003870**		70.45	7,00E-59	184	Protein of unknown function (DUF640)Length=178
SOL Genomics	Sopen	*Solanum pennellii*	**Sopen04g005110**		57.84	8,00E-39	130	Protein of unknown function (DUF640)Length=100
SOL Genomics	Sopim	*Solanum pimpinellifollium*	**Sopim05g055020**		83.45	4,00E-88	259	Light-dependent short hypocotyls 1 (AHRD V1 ***- D7M6V0_ARALY); contains Interpro domain(s) IPR006936 Protein of unknown function DUF640 Length=211
SOL Genomics	Sopim	*Solanum pimpinellifollium*	**Sopim04g009980**		62.36	8,00E-81	239	Light-dependent short hypocotyls 1 (AHRD V1 ***- D7M6V0_ARALY); contains Interpro domain(s) IPR006936 Protein of unknown function DUF640 Length=180
SOL Genomics	Sopim	*Solanum pimpinellifollium*	**Sopim02g069510**		76.47	3,00E-75	225	Light-dependent short hypocotyls 1 (AHRD V1 ***- D7M6V0_ARALY); contains Interpro

Database	Acr	Species	Subject ID	Gene name	Id%	E-value	Score	Description
								domain(s) IPR006936 Protein of unknown function DUF640 Length=181
SOL Genomics	Sopim	*Solanum pimpinellifolium*	**Sopim09g090180**		85.93	6,00E-73	221	Light-dependent short hypocotyls 1 (AHRD V1 ***- D7M6V0_ARALY); contains Interpro domain(s) IPR006936 Protein of unknown function DUF640 Length=208
SOL Genomics	Sopim	*Solanum pimpinellifolium*	**Sopim09g025280**		85.38	2,00E-71	215	Light-dependent short hypocotyls 1 (AHRD V1 ***- D7M6V0_ARALY); contains Interpro domain(s) IPR006936 Protein of unknown function DUF640 Length=171
SOL Genomics	Sopim	*Solanum pimpinellifolium*	**Sopim12g014260**		76.15	1,00E-69	212	Light-dependent short hypocotyls 1 (AHRD V1 ***- D7M6V0_ARALY); contains Interpro domain(s) IPR006936 Protein of unknown function DUF640 Length=205
SOL Genomics	Sopim	*Solanum pimpinellifolium*	**Sopim10g008000**		71.97	4,00E-69	210	Light-dependent short hypocotyls 1 (AHRD V1 ***- D7M6V0_ARALY); contains Interpro domain(s) IPR006936 Protein of unknown function DUF640 Length=178

Database	Acr	Species	Subject ID	Gene name	Id%	E-value	Score	Description
SOL Genomics	Sopim	*Solanum pimpinellifollium*	**Sopim06g082210**		74.29	6,00E-69	211	Light-dependent short hypocotyls 1 (AHRD V1 ***- D7M6V0_ARALY); contains Interpro domain(s) IPR006936 Protein of unknown function DUF640 Length=222
SOL Genomics	Sopim	*Solanum pimpinellifollium*	**Sopim10g007310**		74.62	7,00E-69	210	Light-dependent short hypocotyls 1 (AHRD V1 *-*- D7M6V0_ARALY); contains Interpro domain(s) IPR006936 Protein of unknown function DUF640 Length=213
SOL Genomics	Sopim	*Solanum pimpinellifollium*	**Sopim02g076820**		75.19	2,00E-68	209	Light-dependent short hypocotyls 1 (AHRD V1 ***- D7M6V0_ARALY); contains Interpro domain(s) IPR006936 Protein of unknown function DUF640 Length=192
SOL Genomics	Sopim	*Solanum pimpinellifollium*	**Sopim06g083860**		85.60	2,00E-67	207	Light-dependent short hypocotyls 1 (AHRD V1 *-*- D7M6V0_ARALY); contains Interpro domain(s) IPR006936 Protein of unknown function DUF640 Length=218
SOL Genomics	Sopim	*Solanum pimpinellifollium*	**Sopim07g062470**		64.39	1,00E-59	184	Light-dependent short hypocotyls 1 (AHRD V1 ***- D7M6V0_ARALY); contains Interpro domain(s) IPR006936 Protein of unknown

**Database**	**Acr**	**Species**	**Subject ID**	**Gene name**	**Id%**	**E-value**	**Score**	**Description**
								function DUF640 Length=147
NCBI	Soste	*Solanum stenotomum*	**XP_049412069.1**		85.71%	2,00E-86	253	protein LIGHT-DEPENDENT SHORT HYPOCOTYLS 5 [Solanum stenotomum]
NCBI	Soste	*Solanum stenotomum*	**XP_049383836.1**		78.95%	1,00E-78	232	protein LIGHT-DEPENDENT SHORT HYPOCOTYLS 4-like [Solanum stenotomum]
NCBI	Soste	*Solanum stenotomum*	**XP_049408130.1**		80.29%	1,00E-77	231	protein LIGHT-DEPENDENT SHORT HYPOCOTYLS 4 [Solanum stenotomum]
NCBI	Soste	*Solanum stenotomum*	**XP_049414926.1**		65.58%	6,00E-74	220	protein LIGHT-DEPENDENT SHORT HYPOCOTYLS 10-like [Solanum stenotomum]
NCBI	Soste	*Solanum stenotomum*	**XP_049404186.1**		61.59%	9,00E-74	221	protein LIGHT-DEPENDENT SHORT HYPOCOTYLS 1-like [Solanum stenotomum]
NCBI	Soste	*Solanum stenotomum*	**XP_049406686.1**		76.30%	7,00E-72	216	protein LIGHT-DEPENDENT SHORT HYPOCOTYLS 3-like [Solanum stenotomum]
NCBI	Soste	*Solanum stenotomum*	**XP_049391016.1**		59.51%	6,00E-71	213	protein LIGHT-DEPENDENT SHORT HYPOCOTYLS 1-like [Solanum stenotomum]
NCBI	Soste	*Solanum stenotomum*	**XP_049414161.1**		69.01%	2,00E-70	212	protein LIGHT-DEPENDENT SHORT HYPOCOTYLS 10-like [Solanum stenotomum]
NCBI	Soste	*Solanum stenotomum*	**XP_049401865.1**		66.23%	4,00E-70	212	protein LIGHT-DEPENDENT SHORT HYPOCOTYLS 10-like [Solanum stenotomum]

Database	Acr	Species	Subject ID	Gene name	Id%	E-value	Score	Description
NCBI	Soste	*Solanum stenotomum*	**XP_049402554.1**		58.29%	9,00E-70	210	protein LIGHT-DEPENDENT SHORT HYPOCOTYLS 10-like [Solanum stenotomum]
NCBI	Soste	*Solanum stenotomum*	**XP_049385482.1**		66.67%	2,00E-69	209	protein LIGHT-DEPENDENT SHORT HYPOCOTYLS 10-like [Solanum stenotomum]
NCBI	Soste	*Solanum stenotomum*	**XP_049409865.1**		78.40%	3,00E-69	209	protein LIGHT-DEPENDENT SHORT HYPOCOTYLS 4-like [Solanum stenotomum]
NCBI	Soste	*Solanum stenotomum*	**XP_049404205.1**		72.09%	5,00E-69	209	protein LIGHT-DEPENDENT SHORT HYPOCOTYLS 10-like [Solanum stenotomum]
SOL Genomics	Sotub	*Solanum tuberosum*	**Sotub05g028090.1.1**		84.03	5,00E-88	257	Light-dependent short hypocotyls 1 (AHRD V1 ***- D7M6V0_ARALY); contains Interpro domain(s) IPR006936 Protein of unknown function DUF640Length=209
SOL Genomics	Sotub	*Solanum tuberosum*	**Sotub09g025780.1.1**		79.11	1,00E-77	229	Light-dependent short hypocotyls 1 (AHRD V1 ***- D7M6V0_ARALY); contains Interpro domain(s) IPR006936 Protein of unknown function DUF640Length=175
SOL Genomics	Sotub	*Solanum tuberosum*	**Sotub06g034320.1.1**		85.38	2,00E-71	215	Light-dependent short hypocotyls 1 (AHRD V1 ***- D7M6V0_ARALY); contains Interpro domain(s) IPR006936 Protein of unknown

Database	Acr	Species	Subject ID	Gene name	Id%	E-value	Score	Description
								function DUF640Length=207
SOL Genomics	Sotub	*Solanum tuberosum*	**Sotub09g019540.1.1**		84.21	3,00E-72	215	Light-dependent short hypocotyls 1 (AHRD V1 ***- D7M6V0_ARALY); contains Interpro domain(s) IPR006936 Protein of unknown function DUF640Length=164
SOL Genomics	Sotub	*Solanum tuberosum*	**Sotub06g032980.1.1**		70.39	8,00E-70	211	Light-dependent short hypocotyls 1 (AHRD V1 ***- D7M6V0_ARALY); contains Interpro domain(s) IPR006936 Protein of unknown function DUF640Length=223
SOL Genomics	Sotub	*Solanum tuberosum*	**Sotub04g009970.1.1**		60.87	2,00E-81	239	Light-dependent short hypocotyls 1 (AHRD V1 ***- D7M6V0_ARALY); contains Interpro domain(s) IPR006936 Protein of unknown function DUF640Length=183
SOL Genomics	Sotub	*Solanum tuberosum*	**Sotub02g015740.1.1**		75.18	1,00E-77	229	Light-dependent short hypocotyls 1 (AHRD V1 ***- D7M6V0_ARALY); contains Interpro domain(s) IPR006936 Protein of unknown function DUF640Length=175
SOL Genomics	Sotub	*Solanum tuberosum*	**Sotub12g005130.1.1**		76.15	4,00E-71	214	Light-dependent short hypocotyls 1 (AHRD V1 ***- D7M6V0_ARALY); contains Interpro

Database	Acr	Species	Subject ID	Gene name	Id%	E-value	Score	Description
								domain(s) IPR006936 Protein of unknown function DUF640Length=204
SOL Genomics	Sotub	*Solanum tuberosum*	**Sotub07g022110.1.1**		72.14	5,00E-71	212	Light-dependent short hypocotyls 1 (AHRD V1 ***- D7M6V0_ARALY); contains Interpro domain(s) IPR006936 Protein of unknown function DUF640Length=173
SOL Genomics	Sotub	*Solanum tuberosum*	**Sotub07g024870.1.1**		71.32	3,00E-66	201	Light-dependent short hypocotyls 1 (AHRD V1 ***- D7M6V0_ARALY); contains Interpro domain(s) IPR006936 Protein of unknown function DUF640Length=191
SOL Genomics	Sotub	*Solanum tuberosum*	**Sotub02g019400.1.1**		75.38	1,00E-70	212	Light-dependent short hypocotyls 1 (AHRD V1 ***- D7M6V0_ARALY); contains Interpro domain(s) IPR006936 Protein of unknown function DUF640Length=191
SOL Genomics	Sotub	*Solanum tuberosum*	**Sotub10g007890.1.1**		69.23	2,00E-70	212	Light-dependent short hypocotyls 1 (AHRD V1 ***- D7M6V0_ARALY); contains Interpro domain(s) IPR006936 Protein of unknown function DUF640Length=207
SOL Genomics	Sotub	*Solanum tuberosum*	**Sotub10g008420.1.1**		70.45	7,00E-70	209	Light-dependent short hypocotyls 1 (AHRD V1

Database	Acr	Species	Subject ID	Gene name	Id%	E-value	Score	Description
								***- D7M6V0_ARALY); contains Interpro domain(s) IPR006936 Protein of unknown function DUF640Length=178
SOL Genomics	Sotub	*Solanum tuberosum*	Sotub10g008430.1.1		70.45	7,00E-70	209	Light-dependent short hypocotyls 1 (AHRD V1 ***- D7M6V0_ARALY); contains Interpro domain(s) IPR006936 Protein of unknown function DUF640Length=178
Phytozome	Sobic	*Sorghum bicolor*	**Sobic.006G147500.1**		77.8	3.9E-69	213.0	(Sb06g022610) similar to OSJNBb0072M01.12 protein
Phytozome	Sobic	*Sorghum bicolor*	**Sobic.001G219400.1**		83.5	5.6E-69	213.4	(Sb01g019290) similar to Putative uncharacterized protein OSJNBa0055P24.1
Phytozome	Sobic	*Sorghum bicolor*	**Sobic.004G219600.1**		81.6	4.3E-68	210.7	(Sb04g026450) similar to Os02g0623400 protein
Phytozome	Sobic	*Sorghum bicolor*	**Sobic.004G052800.1**		82.4	2.3E-67	210.7	(Sb04g004470) similar to Putative uncharacterized protein
Phytozome	Sobic	*Sorghum bicolor*	**Sobic.003G342600.2**		79.4	2.9E-67	208.8	(Sb03g038670) similar to Putative uncharacterized protein
Phytozome	Sobic	*Sorghum bicolor*	**Sobic.004G338400.1**		82.3	5.2E-66	206.8	(Sb04g036620) similar to Putative uncharacterized protein
Phytozome	Sobic	*Sorghum bicolor*	**Sobic.003G421300.1**		82.4	7.7E-66	207.6	(Sb03g045430) (1 of 12) PF04852 - Protein of unknown function (DUF640)

Database	Acr	Species	Subject ID	Gene name	Id%	E-value	Score	Description
Phytozome	Sobic	*Sorghum bicolor*	**Sobic.010G225100.1**		82.4	7.7E-66	207.6	(1 of 12) PF04852 - Protein of unknown function (DUF640)
Phytozome	Sobic	*Sorghum bicolor*	**Sobic.009G102600.1**		77.9	2.4E-65	207.6	(Sb09g016440) similar to Putative uncharacterized protein
Phytozome	Sobic	*Sorghum bicolor*	**Sobic.009G170100.1**		81.1	1.1E-64	203.8	(Sb09g023120) similar to Putative uncharacterized protein
Phytozome	Sobic	*Sorghum bicolor*	**Sobic.005G094266.1**		69.9	1.4E-58	185.3	(1 of 2) PTHR31165:SF6 - PROTEIN LIGHT-DEPENDENT SHORT HYPOCOTYLS 6
Phytozome	Sobic	*Sorghum bicolor*	**Sobic.002G029700.1**	SbG1	52.8	1.2E-42	147.5	(Sb02g002650) similar to Putative uncharacterized protein OJ1417_E01.118
Phytozome	Spfal	*Sphagnum fallax*	**Sphfalx0011s0170.1**		75.9	1.2E-65	204.9	(1 of 5) PF04852 - Protein of unknown function (DUF640) (DUF640)
Phytozome	Spfal	*Sphagnum fallax*	**Sphfalx0049s0047.1**		68.5	9.8E-65	202.2	(1 of 5) PF04852 - Protein of unknown function (DUF640) (DUF640)
Phytozome	Spfal	*Sphagnum fallax*	**Sphfalx0116s0084.1**		76.7	2.2E-64	201.8	(1 of 5) PF04852 - Protein of unknown function (DUF640) (DUF640)
Phytozome	Spfal	*Sphagnum fallax*	**Sphfalx0222s0016.1**		77.1	2.6E-64	205.3	(1 of 5) PF04852 - Protein of unknown function (DUF640) (DUF640)
Phytozome	Spfal	*Sphagnum fallax*	**Sphfalx0005s0338.1**		75.6	3,00E-64	200.7	(1 of 5) PF04852 - Protein of unknown function (DUF640) (DUF640)
Phytozome	Spmag	*Sphagnum magellanicum*	**Sphmag18G043300.1.p**		80	9.21e-77	230.335	(1 of 5) PF04852 - Protein of unknown function (DUF640) (DUF640)
Phytozome	Spmag	*Sphagnum magellanicum*	**Sphmag08G006300.1.p**		78	4.37e-67	205.682	(1 of 5) PF04852 - Protein of unknown function (DUF640) (DUF640)

Database	Acr	Species	Subject ID	Gene name	Id%	E-value	Score	Description
Phytozome	Spmag	*Sphagnum magellanicum*	**Sphmag05G126700.1.p**		78	2.65e-74	223.787	(1 of 5) PF04852 - Protein of unknown function (DUF640) (DUF640)
Phytozome	Spmag	*Sphagnum magellanicum*	**Sphmag03G062200.1.p**		79	1.37e-69	212.231	(1 of 5) PF04852 - Protein of unknown function (DUF640) (DUF640)
Phytozome	Spmag	*Sphagnum magellanicum*	**Sphmag02G037800.1.p**		78	6.35e-67	204.912	(1 of 5) PF04852 - Protein of unknown function (DUF640) (DUF640)
PhycoCosm	Spmus	*Spirogloea muscicola*	**jgi|Spimu1|13|SM000001S04405**		75.41	4,70E-57	265	
PhycoCosm	Spmus	*Spirogloea muscicola*	**jgi|Spimu1|11189|SM000023S07704**		75.41	4,70E-57	265	
PhycoCosm	Spmus	*Spirogloea muscicola*	**jgi|Spimu1|15861|SM000034S12670**		75.41	4,70E-57	265	
	Sppra	*Spirogyra pratensis*	GBSM01019623_1					
Phytozome	Thpli	*Thuja plicata*	**Thupl.29382294s0001.1.p**		75	7.27e-82	243.432	(1 of 3) PF04852 - Protein of unknown function (DUF640) (DUF640)
Phytozome	Thpli	*Thuja plicata*	**Thupl.29382466s0011.1.p**		80	7.62e-71	214.927	(1 of 3) PF04852 - Protein of unknown function (DUF640) (DUF640)
Phytozome	Thpli	*Thuja plicata*	**Thupl.29378893s0002.1.p**		82	4.70e-67	205.682	(1 of 3) PF04852 - Protein of unknown function (DUF640) (DUF640)
Phytozome	Trpra	*Trifolium pratense*	Tp57577_TGAC_v2_mRNA14397		74.4	4.1E-58	184.1	(1 of 1) PTHR31165:SF9 - PROTEIN LIGHT-DEPENDENT SHORT HYPOCOTYLS 7-RELATED
Phytozome	Trpra	*Trifolium pratense*	Tp57577_TGAC_v2_mRNA24596		77.3	1.8E-67	209.1	(1 of 14) PF04852 - Protein of unknown function (DUF640) (DUF640)
Phytozome	Trpra	*Trifolium pratense*	Tp57577_TGAC_v2_mRNA11916		75.2	1.1E-61	192.6	(1 of 3) PTHR31165:SF10 -

Database	Acr	Species	Subject ID	Gene name	Id%	E-value	Score	Description
								PROTEIN LIGHT-DEPENDENT SHORT HYPOCOTYLS 10-RELATED
Phytozome	Trpra	*Trifolium pratense*	Tp57577_TGAC_v2_mRNA35933		72.9	6.4E-65	205.7	(1 of 2) PTHR31165:SF6 - PROTEIN LIGHT-DEPENDENT SHORT HYPOCOTYLS 6
Phytozome	Trpra	*Trifolium pratense*	Tp57577_TGAC_v2_mRNA26043		88	5.1E-68	210.3	(1 of 3) PTHR31165:SF13 - PROTEIN LIGHT-DEPENDENT SHORT HYPOCOTYLS 1-RELATED
Phytozome	Trpra	*Trifolium pratense*	Tp57577_TGAC_v2_mRNA12167		68.1	1.5E-57	182.6	(1 of 14) PF04852 - Protein of unknown function (DUF640) (DUF640)
Phytozome	Trpra	*Trifolium pratense*	Tp57577_TGAC_v2_mRNA14788		83.8	2.2E-71	218.8	(1 of 3) PTHR31165:SF13 - PROTEIN LIGHT-DEPENDENT SHORT HYPOCOTYLS 1-RELATED
Phytozome	Trpra	*Trifolium pratense*	Tp57577_TGAC_v2_mRNA39651		83.1	2.4E-67	209.1	(1 of 14) PF04852 - Protein of unknown function (DUF640) (DUF640)
Phytozome	Trpra	*Trifolium pratense*	Tp57577_TGAC_v2_mRNA29264		66.2	1.4E-55	176.8	(1 of 3) PTHR31165:SF10 - PROTEIN LIGHT-DEPENDENT SHORT HYPOCOTYLS 10-RELATED
Phytozome	Trpra	*Trifolium pratense*	Tp57577_TGAC_v2_mRNA14571		31.3	9.2E-2	32.7	(1 of 4) PTHR31190:SF21 - ETHYLENE-RESPONSIVE

Database	Acr	Species	Subject ID	Gene name	Id%	E-value	Score	Description
								TRANSCRIPTION FACTOR 13
Phytozome	Trpra	*Trifolium pratense*	Tp57577_TGAC_v2_mRNA7237		68.4	7.8E-61	190.7	(1 of 14) PF04852 - Protein of unknown function (DUF640) (DUF640)
Phytozome	Trpra	*Trifolium pratense*	Tp57577_TGAC_v2_mRNA23093		86.8	4.2E-72	219.9	(1 of 14) PF04852 - Protein of unknown function (DUF640) (DUF640)
Phytozome	Trpra	*Trifolium pratense*	Tp57577_TGAC_v2_mRNA1495		88.9	2.7E-69	213.0	(1 of 3) PTHR31165:SF13 - PROTEIN LIGHT-DEPENDENT SHORT HYPOCOTYLS 1-RELATED
Phytozome	Trpra	*Trifolium pratense*	Tp57577_TGAC_v2_mRNA33996		76.9	3.3E-64	200.7	(1 of 2) PTHR31165:SF6 - PROTEIN LIGHT-DEPENDENT SHORT HYPOCOTYLS 6
Phytozome	Trpra	*Trifolium pratense*	Tp57577_TGAC_v2_mRNA25252		72	7.7E-57	181.0	(1 of 3) PTHR31165:SF10 - PROTEIN LIGHT-DEPENDENT SHORT HYPOCOTYLS 10-RELATED
Phytozome	Vadar	*Vaccinium darrowii*	**Vadar_g41849.t1**		74	2.74e-82	243.047	(1 of 4) PTHR31165//PTHR31165:SF6 - FAMILY NOT NAMED // PROTEIN LIGHT-DEPENDENT SHORT HYPOCOTYLS 6
Phytozome	Vadar	*Vaccinium darrowii*	**Vadar_g6489.t1**		78	3.78e-77	230.335	(1 of 4) PTHR31165:SF6 - PROTEIN LIGHT-DEPENDENT SHORT HYPOCOTYLS 6

Database	Acr	Species	Subject ID	Gene name	Id%	E-value	Score	Description
Phytozome	Vadar	*Vaccinium darrowii*	**Vadar_g8949.t1**		71	1.34e-73	219.935	(1 of 1) PTHR31165//PTHR31165:SF10 - FAMILY NOT NAMED // PROTEIN LIGHT-DEPENDENT SHORT HYPOCOTYLS 10-RELATED
Phytozome	Vadar	*Vaccinium darrowii*	**Vadar_g40955.t1**		83	6.32e-68	207.994	(1 of 15) PF04852 - Protein of unknown function (DUF640) (DUF640)
Phytozome	Vadar	*Vaccinium darrowii*	**Vadar_g6818.t1**		78	6.89e-68	206.068	(1 of 15) PF04852 - Protein of unknown function (DUF640) (DUF640)
Phytozome	Vadar	*Vaccinium darrowii*	**Vadar_g31844.t1**		78	8.33e-68	205.682	(1 of 5) PTHR31165//PTHR31165:SF13 - FAMILY NOT NAMED // PROTEIN LIGHT-DEPENDENT SHORT HYPOCOTYLS 1-RELATED
Phytozome	Vadar	*Vaccinium darrowii*	**Vadar_g31853.t1**		78	8.33e-68	205.682	(1 of 5) PTHR31165:SF13 - PROTEIN LIGHT-DEPENDENT SHORT HYPOCOTYLS 1-RELATED
Phytozome	Vadar	*Vaccinium darrowii*	**Vadar_g31850.t1**		77	3.77e-67	203.756	(1 of 5) PTHR31165//PTHR31165:SF13 - FAMILY NOT NAMED // PROTEIN LIGHT-DEPENDENT SHORT HYPOCOTYLS 1-RELATED
Phytozome	Vadar	*Vaccinium darrowii*	**Vadar_g31852.t1**		77	3.77e-67	203.756	(1 of 5) PTHR31165//PTHR31165:SF13 - FAMILY NOT

Database	Acr	Species	Subject ID	Gene name	Id%	E-value	Score	Description
								NAMED // PROTEIN LIGHT-DEPENDENT SHORT HYPOCOTYLS 1-RELATED
Phytozome	Vadar	*Vaccinium darrowii*	**Vadar_g12997.t1**		68	1.67e-66	201.83	(1 of 2) IPR006936//IPR009097 - ALOG domain // RNA ligase/cyclic nucleotide phosphodiesterase
Phytozome	Vadar	*Vaccinium darrowii*	**Vadar_g8068.t1**		67	1.25e-64	196.823	(1 of 1) PTHR31165:SF9 - PROTEIN LIGHT-DEPENDENT SHORT HYPOCOTYLS 7-RELATED
Phytozome	Vadar	*Vaccinium darrowii*	**Vadar_g36225.t1**		71	8.45e-64	194.897	(1 of 5) PTHR31165:SF13 - PROTEIN LIGHT-DEPENDENT SHORT HYPOCOTYLS 1-RELATED
Phytozome	Vadar	*Vaccinium darrowii*	**Vadar_g42213.t1**		79	1.03e-61	190.66	(1 of 15) PF04852 - Protein of unknown function (DUF640) (DUF640)
Phytozome	Vadar	*Vaccinium darrowii*	Vadar_g35986.t1		60	6.33e-21	828.037	(1 of 4) PTHR31165//PTHR31165:SF6 - FAMILY NOT NAMED // PROTEIN LIGHT-DEPENDENT SHORT HYPOCOTYLS 6
Phytozome	Vadar	*Vaccinium darrowii*	Vadar_g42008.t1		60	6.33e-21	828.037	(1 of 4) PTHR31165//PTHR31165:SF6 - FAMILY NOT NAMED // PROTEIN LIGHT-DEPENDENT SHORT HYPOCOTYLS 6

**Database**	**Acr**	**Species**	**Subject ID**	**Gene name**	**Id%**	**E-value**	**Score**	**Description**
Phytozome	Zemay	*Zea mays*	**Zm00008a016736_T01**		81.9	1.9E-55	178.3	(1 of 19) PF04852 - Protein of unknown function (DUF640)
Phytozome	Zemay	*Zea mays*	**Zm00008a022360_T01**		81.7	1.8E-55	178.7	(1 of 19) PF04852 - Protein of unknown function (DUF640)
Phytozome	Zemay	*Zea mays*	**Zm00008a034931_T01**		83.5	1.1E-55	179.5	(1 of 19) PF04852 - Protein of unknown function (DUF640)
Phytozome	Zemay	*Zea mays*	**Zm00008a020995_T01**		78.6	2.6E-61	194.5	(1 of 19) PF04852 - Protein of unknown function (DUF640)
Phytozome	Zemay	*Zea mays*	**Zm00008a032984_T01**		77.7	2.9E-63	198.0	(1 of 19) PF04852 - Protein of unknown function (DUF640)
Phytozome	Zemay	*Zea mays*	**Zm00008a004172_T01**		81.2	2.9E-64	200.7	(1 of 19) PF04852 - Protein of unknown function (DUF640)
Phytozome	Zemay	*Zea mays*	**Zm00008a025661_T01**		77.9	1,00E-64	204.9	(1 of 19) PF04852 - Protein of unknown function (DUF640)
Phytozome	Zemay	*Zea mays*	**Zm00008a017392_T01**		82.3	8.8E-66	205.7	(1 of 19) PF04852 - Protein of unknown function (DUF640)
Phytozome	Zemay	*Zea mays*	**Zm00008a023299_T01**		82.3	2.8E-66	208.0	(1 of 19) PF04852 - Protein of unknown function (DUF640)
Phytozome	Zemay	*Zea mays*	**Zm00008a013434_T01**		77.8	4.6E-68	210.3	(1 of 19) PF04852 - Protein of unknown function (DUF640)
Phytozome	Zemay	*Zea mays*	**Zm00008a024573_T01**		82.4	8.4E-66	210.3	(1 of 19) PF04852 - Protein of unknown function (DUF640)
Phytozome	Zemay	*Zea mays*	Zm00008a025985_T01		75.2	4.3E-61	194.1	(1 of 19) PF04852 - Protein of unknown function (DUF640)

**Database**	**Acr**	**Species**	**Subject ID**	**Gene name**	**Id%**	**E-value**	**Score**	**Description**
Phytozome	Zemay	*Zea mays*	Zm00008a038244_T01		64.6	2.6E-57	181.8	(1 of 19) PF04852 - Protein of unknown function (DUF640)
Phytozome	Zemay	*Zea mays*	Zm00008a031466_T01		80.6	1.7E-47	159.5	(1 of 2) PTHR31165:SF6 - PROTEIN LIGHT-DEPENDENT SHORT HYPOCOTYLS 6
Phytozome	Zemay	*Zea mays*	Zm00008a031532_T01		77	1,00E-24	96.7	(1 of 19) PF04852 - Protein of unknown function (DUF640)
Phytozome	Zomar	*Zostera marina*	**Zosma114g00940.1**		81	1.02e-79	237.269	(1 of 9) PF04852 - Protein of unknown function (DUF640) (DUF640)
Phytozome	Zomar	*Zostera marina*	**Zosma175g00330.1**		83	3.04e-77	230.72	(1 of 1) PTHR31165:SF6 - PROTEIN LIGHT-DEPENDENT SHORT HYPOCOTYLS 6
Phytozome	Zomar	*Zostera marina*	**Zosma14g01840.1**		83	1.31e-75	225.713	Protein of unknown function (DUF640)
Phytozome	Zomar	*Zostera marina*	**Zosma30g01180.1**		82	8.25e-73	221.09	Protein of unknown function (DUF640)
Phytozome	Zomar	*Zostera marina*	**Zosma16g00570.1**		81	6.54e-72	218.009	Protein of unknown function (DUF640)
Phytozome	Zomar	*Zostera marina*	**Zosma110g00270.1**		78	1.07e-70	214.157	Protein of unknown function (DUF640)
Phytozome	Zomar	*Zostera marina*	**Zosma117g00780.1**		79	2.93e-70	213.386	Protein of unknown function (DUF640)
Phytozome	Zomar	*Zostera marina*	**Zosma445g00080.1**		79	2.15e-69	209.149	Protein of unknown function (DUF640)
Phytozome	Zomar	*Zostera marina*	**Zosma506g00030.1**		77	1.88e-67	203.756	Protein of unknown function (DUF640)
PhycoCosm	Zycyl	*Zygnema cf. cylindricum* SAG 698-1a	**jgi|Zygcyl6981a_1|4961|Zci1a_43187.1**		36.78	1.06E-051	468	Zygcyl6981a_1_GeneCatalog_proteins_20230203.aa
PhycoCosm	Zycyl	*Zygnema cf. cylindricum* SAG 698-1a	**jgi|Zygcyl6981a_1|43728|Zci1a_16452.1**		44.91	1.16E-039	380	Zygcyl6981a_1_GeneCatalog_proteins_20230203.aa

**Database**	**Acr**	**Species**	**Subject ID**	**Gene name**	**Id%**	**E-value**	**Score**	**Description**
PhycoCosm	Zycir	*Zygnema circumcarinatum* SAG 698-1b	**jgi|Zygcir6981b_1|15080|Zci_06105.1**		30.12	1.48E-055	496	Zygcir6981b_1_GeneCatalog_proteins_20230201.aa
