# Supplementary material for: Genome-wide identification and evolutionary view of ALOG gene family in Solanaceae
Source: Genet Mol Biol. 2023 Dec 1;46(3 Suppl 1):e20230142. doi: 10.1590/1415-4757-GMB-2023-0142 (PMC10695626; doi:10.1590/1415-4757-GMB-2023-0142)
Supplement: Figure S3 - [file 1415-4757-GMB-46-3-s1-e20230142-s6.pdf]

Genetics and Molecular Biology

https://doi.org/10.1590/1678-4685-GMB-2023-0142

Supplementary Material to “Genome-wide identification and evolutionary view of ALOG gene family in Solanaceae”

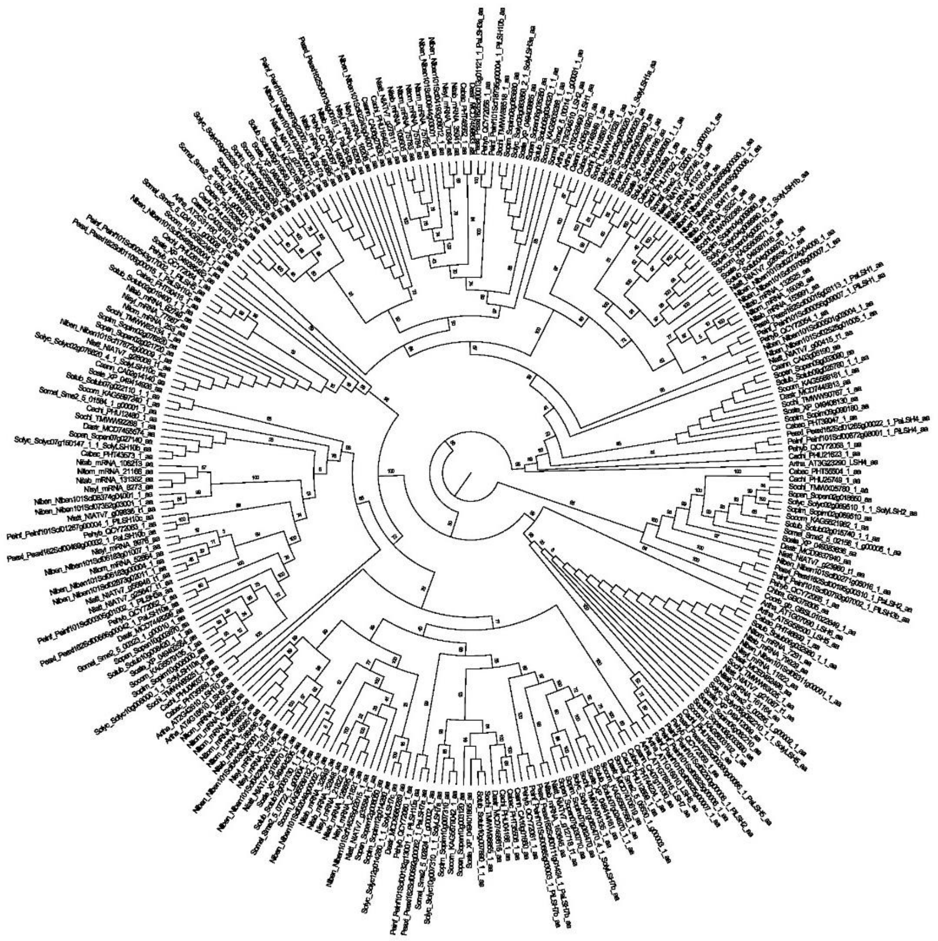

Figure S3 - Phylogenetic tree of ALOG gene family in Solanaceae showing the branches support.
